# High functionality of DNA barcodes and revealed cases of cryptic diversity in Korean curved-horn moths (Lepidoptera: Gelechioidea)

**DOI:** 10.1038/s41598-020-63385-x

**Published:** 2020-04-10

**Authors:** Sora Kim, Yerim Lee, Marko Mutanen, Jinbae Seung, Seunghwan Lee

**Affiliations:** 1 Laboratory of Insect Biosystematics, Department of Agricultural Biotechnology, Research Institute of Agriculture and Life Sciences, Seoul National University, Seoul, 08826 Republic of Korea; 2 Ecology and Genetics Research Unit, PO Box 3000, FI-90014, University of Oulu, Oulu, Finland

**Keywords:** Molecular biology, Entomology

## Abstract

Curved-horn moths or gelechioid moths (Lepidoptera: Gelechioidea) represent one of the most diverse lepidopteran groups. Due to the large number of species, generally small size of adults and subtle morphological differences, their confident identification requires tenacious and long-term dedication on their diversity. Over the past decade, DNA barcoding has repeatedly been used to elucidate boundaries of species in many large and difficult groups. Here, we conducted a test of DNA barcoding with the diverse fauna of Korean Gelechioidea with very little prior information of COI gene region from the area. Altogether 509 specimens representing 154 morphospecies were included in the study. The species assignments of all three tested species delimitation methods (ABGD, bPTP and PTP) were consistent with morphological identifications for 117 species (75.97%). A threshold of 2.5% genetic divergence was observed to differentiate the morphological species efficiently. Careful morphological examination of morphospecies exceeding 2.5% intraspecific variability prove cryptic diversity in three species (*Neoblastobasis biceratala, Evippe albidoesella* and *Promalactis atriplagata*). One morphospecies, *Promalactis odaiensis*, showed high intraspecific divergence while consisted of only a single MOTU. Overall, DNA barcoding was shown to provide a powerful tool to discriminate species of Korean Gelechioidea and reveal cases of cryptic diversity.

## Introduction

Curved-horn moths (Gelechioidea) is among the most species-rich superfamilies of the insect order Lepidoptera, containing 15–21 families^1–3^. They are generally regarded as ‘micro-moths’, and their life histories are greatly diverse as they occupy wide range of both terrestrial and aquatic habitats. The larvae may be external and internal feeders and larvae include concealers, case-bearers, twirlers, gall-makers and miners of vascular plants, mosses, lichens, seeds, dead plant materials and dung. Some species are scavengers or even predators^4^. The superfamily has a worldwide distribution, comprising more than 18,000 described species^2^. Typically, their adults have well-developed labial palps gently bent upward, giving an appearance of ‘curved horn’. Species include many of agricultural, forestry and quarantine pests causing widespread damage around the world. The larvae of many species, such as *Phthorimaea operculella* (potato tuber moth), *Scrobipalpa aptatella* (tabacco stem borer) and *Endrosis sarcitrella* (white-shouldered house moth) cause serious damage to crops and stored grains by mining the leaves or burrowing into the seeds or fruits. Because of their usually tiny body size, indistinct appearance and cryptic behavior of adults, their identification to the species level is often difficult based on morphology.

Over the past decade, DNA barcoding has developed to serve as an efficient troubleshooter in precise and fast identification of species, discoveries of cryptic species and surveys of biodiversity in a wide variety animal taxa and some other eukaryote groups^5–16^. In particular, DNA barcoding has contributed a lot to the inventories of diversity of the Lepidoptera (butterflies and moths), one of the megadiverse insect groups comprising more than 157,000 described species. Most studies of Lepidoptera have focused on West Palearctic^17–21^, Neartic^22^ and Neotropical faunas^23–25^, while rich Asian fauna has remained poorly investigated^26–28^.

Korean Peninsula is located at a temperate zone in Eastern Eurasia, extending southwards for about 1,100 km from continental Asia into the Pacific Ocean. It surrounded by the East Sea to the East and the Yellow Sea to the west, by connecting the two bodies of water^29^. Its nature is characterized by relatively warm climate, strong seasonal changes and rich biological diversity, with approximately 50,000 biological species including over 2,300 endemic species. Also, as about 80% of the land area consists of mountains, altitudinal changes are noticeable.

We aimed to test the utility of DNA barcoding in species identification on a large dataset from 154 morphospecies of Gelechioidea occurring in Korea. The Gelechioidea was selected to serve as a model group as the species are usually dull-coloured and small, their identification is time-consuming, they include a number of morphologically complex groups and they are likely to include cryptic diversity. Comprehensive DNA barcode reference library of them is expected to be highly useful in their identification in the future. We hope our barcoding study on Korean Gelechioidea could serve as a model for large-scale systematic investigations of microlepidopteran diversity in Asia, which so far has remained poorly studied with molecular tools. Additionally, we aimed to focus on detecting potential cases of cryptic species and testing the efficacy of DNA barcoding with difficult species complexes. Finally, we applied several algorithm-based species delimitation methods and searched for the optimal divergence threshold value for species delimitation.

## Material and Methods

### Specimen collection and morphospecies identification

**Figure 1:**
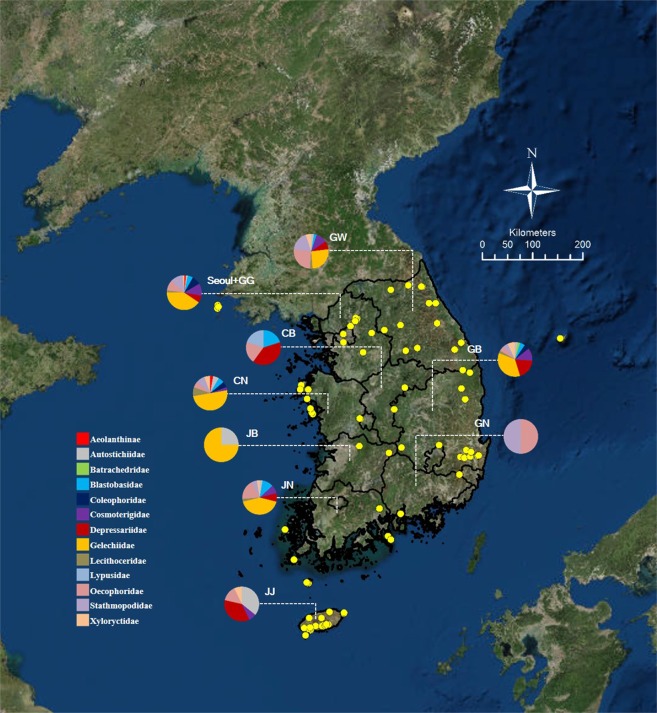
Map with 85 localities for 509 specimens during 2009–2017. Pie charts show the proportion of each family at respective locations. The abbreviations used here for administrative districts in Korea are as follows: GG, Gyeonggi-do; GW, Gangweon-do; CB, Chungcheongbuk-do; CN, Chungcheongnam-do; JB, Jeonlabuk-do; JN, Jeonlanam-do; GB, Gyeongsangbuk-do; GN, Gyeongsangnam-do; JJ, Jeju-do. (A map was prepared in ArcGIS 10.1.(www.esri.com).).

Altogether 509 individuals were collected from 85 locations of 10 administrative districts in Korea during 2009–2017 (Fig. 1, Supplementary Table S1 online). The collection was performed mostly using light collecting (mercury vapor lamp, 220 V/ 400 W) or bucket light trap (black light lamp, 20 W). Before DNA isolation, all specimens were mounted, examined and photographed for identification under microscope (DM 4000B, Leica Microsystems, Wetzlar, Germany) with a software application, 18.3 Three Shot Color (Diagnostic Instruments, Sterling Heights, MI, USA). Slides of genitalia of vouchers of all species for identification were made by the first author. The classification follows recent phylogenetic works^3,30^. All specimens with vouchers are deposited in the College of Agriculture and Life Sciences, Seoul National University (CALS SNU) and Korea National Arboretum (KNA), Republic of Korea.

### DNA extraction, polymerase chain reaction and sequencing

Genomic DNA was extracted by grinding up usually legs or head or whole body except abdomen of dried specimens using DNeasy Blood and Tissue kit (QIAGEN, Hilden, Germany) according to the manufacturer’s protocols. The primer pair LCO1490 and HCO2198^31^ amplified the standard 667 bp invertebrate barcode near the 5’ end of the mtCOI gene. Amplification was performed in a PCR reaction mix (AccuPower PCR PreMix (Bioneer, Daejeon, Republic of Korea) for a volume of 20 µL containing DNA polymerase, 250 µm of dNTP for each sample, a tracking dye and reaction buffer with 1.5 mM MgCl_2._ The thermal cycling program consisted of initial denaturation at 95 °C for 2 min, followed by 40 cycles of denaturation at 95 °C for 30 s, annealing at 45–55 °C for 30 s, extension at 72 °C for 1 min, and a final extension at 72 °C for 10 min. PCR products were checked in 1.2% agarose gels and purified by QIAquick PCR purification kit (QIAGEN, Hilden, Germany) following the manufacturer’s protocol. Purified samples were sequenced at BIONICS, Inc. (Seongdong-Gu, Seoul, Republic of Korea).

### Sequence analysis and genetic divergence and haplotyping

A total of 509 sequences for 154 morphospecies were generated as novel data in the present study (Supplementary Table S1 online). Raw sequences were assembled and edited using SeqMan^TM^II (version 5.01, 2001; DNA-star^TM^). During the alignment, severely contaminated or very short sequences were excluded to minimize the risk of any kind of confusion and errors. Sequence data were combined using SequenceMatrix windows ver. 1.7.8^32^. The sequences were deposited in GenBank (MK210635 to MK211143) (Supplementary Table S1 online). We implemented Kimura-2 parameter (K2P) model to calculate intra and interspecific pairwise genetic distances because it is computationally fast and, represents the most widely used as a substitution model (www.bold.org). Haplotype data were generated in DnaSP5.10^33^ to identify the unique haplotypes.

### Barcode tree analysis, species delimitation

Neighbor-Joining (NJ) and Maximum Likelihood (ML) analyses were implemented to test the reciprocal monophyletic criteria for species delimitation. The NJ tree was constructed using MEGA 7.0^34^ under K2P model. ML analysis tree was carried out in the CIPRES supercomputing portal with RAxML-HPCv.8 on XSEDE tool^35^.

To estimate the number of molecular operational taxonomic units (MOTUs) from the Gelechioidea dataset, we performed three species delimitation methods, Automatic Barcode Gap Discovery (ABGD)^36^, Poisson-Tree-Processes (PTP)^37^ and Bayesian implementation of the PTP (bPTP).

ABGD analysis for MOTU detection was conducted under JC69, K2P and p-distance substitution models. The ABGD analyzed data based on genetic distance for MOTUs picking and conducted on the web interface (http://wwwabi.snv.jussieu.fr/public/abgd/), with default setting, by K2P, Jukes-Cantor (JC69) and p distance model with relative gap width (X = 1.5). *P* value indicates partition with prior maximal distance. The PTP is a coalescent-based species delimitation method only requires a phylogenetic input tree, and the bPTP is an updated version of the PTP by adding Bayesian support (BS) values to delimited species on the input tree. It uses coalescence theory and assumes that intra- and interspecific substitutions follow two distinct Poisson processes and that intraspecific substitutions are significantly fewer than interspecific substitutions^38,39^. For both analyses, a ML tree was generated as input trees. The web server at (http://species.h-its.org/) was used to run the analyses. Moreover, to investigate a threshold for evaluating the number of MOTUs within Gelechioidea, we examined the maximum intraspecific distance within each of the 154 morphospecies with multiple samples.

## Results

### Morphological identification

Based on the morphological examination, the 509 specimens analyzed in this study were assigned to 154 morphospecies in 64 genera and 13 higher taxa (see Supplementary Table S2 online). The following 12 families were represented: Autostichidae, Batrachedridae, Blastobasidae, Coleophoridae, Cosmopterigidae, Depressariidae, Gelechiidae, Lecithoceridae, Lypusidae, Oecophoridae, Stathmopodidae and Xyloryctidae. Additionally, subfamily Aeolanthinae of unknown family, was also included.

### DNA barcode based identification

Haplotype data analysis revealed 332 distinct haplotypes (see Supplementary Fig. S1 online). Mean K2P genetic divergence (MGD) across all specimens was 0.1340. The MGD increased hierarchically from within species (mean = 0.0077), to within congeners (mean = 0.0963), and within families (mean = 0.1164) (Table 1). K2P genetic divergence values between species within families sampled by more than one species (Autostichidae, Blastobasidae, Coleophoridae, Cosmopterigidae, Depressariidae, Gelechiidae, Lecithoceridae, Oecophoridae, Stathmopodidae and Xyloryctidae) are provided in Table 2. Batrachedridae, Lypusidae and Aeolanthinae are not included here as they each were represented by only one species. Overall, the K2P genetic divergence among the congeneric species was on average approximately 12 times greater than that among individuals of the same species.

**Table 1:** Mean K2P genetic distance in accordance with different taxonomic levels within Gelechioidea.

Comparisons	K2P Mean Genetic Distance (MGD) %
overall	13.40
within species	0.77
within congeners	9.63
within families	11.64

**Table 2:** K2P genetic divergence between species within each family. Batrachedridae, Lypusidae and Aeolanthinae are not included here as they each are represented by one species only.

Comparisons (10)	Mean (%)	Min.(%)	Max. (%)
Autostichiidae	9.06	0.00	11.50
Blastobasidae	9.85	6.41	13.26
Coleophoridae	8.18	4.33	12.56
Cosmopterigidae	11.44	2.77	14.54
Depressariidae	12.47	2.73	21.54
Gelechiidae	12.30	0.00	19.09
Lecithoceridae	16.41	9.14	20.12
Oecophoridae	11.89	5.93	16.18
Stathmopodidae	13.16	3.39	17.51
Xyloryctidae	11.61	9.77	14.01

### MOTUs estimation

**Figure 2:**
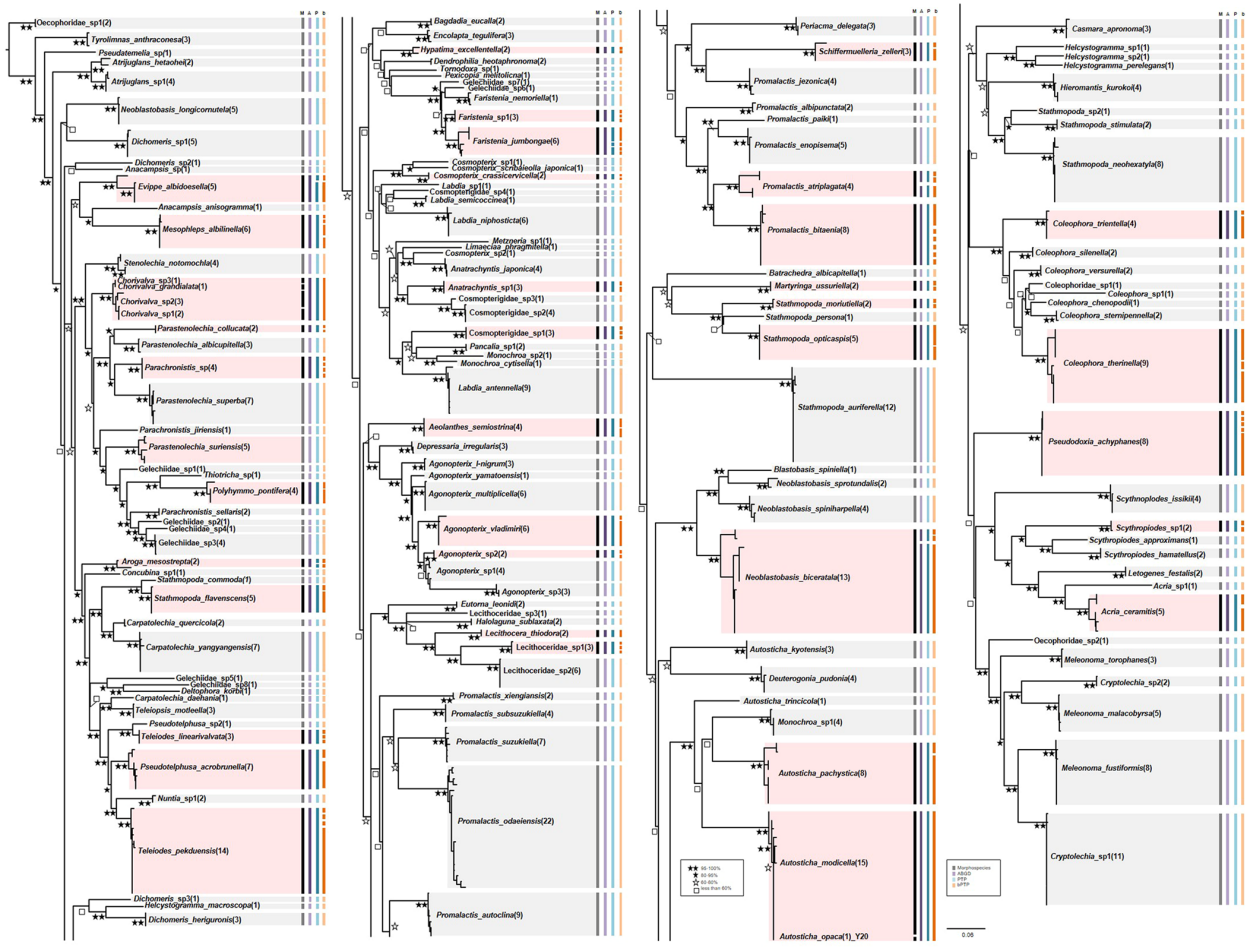
Maximum likelihood tree (ML) on the COI dataset including 509 individuals, with the results of three different species delimitation approaches in addition to morphology (see legend).

The species delimitation methods of ABGD, PTP and bPTP yielded 152, 156 and 213 MOTUs, respectively (Fig. 2). In the ML tree, color bars indicate delineated MOTUs by different methods (Fig. 2). Altogether 117 MOTUs were recovered by each three method, equaling to 75.97% overlap with the morphological delineation of species (Fig. 2). For original ML tree, see Supplementary Fig. S2 online.

ABGD analysis under each JC69, K2P and p-distance substitution model produced 120 MOTUs with *P* = 0.021544, and 152 MOTUs with *P* = 0.0010–0.0215, 0.0010–0.0215 and 0.0129–0.0010, respectively (see Supplementary Table S3 online). Three morphospecies, *Neoblastobasis biceratala*, *Promalactis atriplagata* and *Evippe albidoesella*, were each divided into two MOTUs by ABGD. Conversely, in three cases were 2–4 morphospecies observed to share a single MOTU: *Autosticha modicella* and *A. opaca*; *Agonopterix* sp1 and *Agonopterix* sp2; *Chorivalva unisaccula*, *Chorivalva* sp1, *Chorivalva* sp2 and *C. grandialata*.

PTP analysis resulted in 156 MOTUs in the dataset. Compared with ABGD, additional MOTUs were observed in six species, *Evippe albidoesella*, *Aroga mesostrepta*, *Promalactis atriplagata*, *Faristenia jumbongae*, *Neoblastobasis biceratala* and *Atrijuglans hetaohei*, whereas *Autosticha modicella* and *A. opaca* as well as a morphospecies quartet *Chorivalva unisaccula*, *Chorivalva* sp1, *Chorivalva* sp2 and *C. grandialata*, were each recovered under a single MOTU by PTP.

bPTP discovered 213 MOTUs, which is a clearly higher number than that of other species delimitation methods. Additional MOTU was found in 37 morphospecies (see Supplementary Table S4 online). In common to the two other methods, three species, *Neoblastobasis biceratala*, *Evippe albidoesella* and *Promalactis atriplagata*, were each split into multiple MOTUs. Like in PTP, the morphospecies pair *Autosticha modicella* and *A. opaca* and the morphospecies quartet *Chorivalva unisaccula*, *Chorivalva* sp1 and *Chorivalva* sp2 and *C. grandialata* were each assigned under a single MOTU.

### Detection of cryptic species

Altogether 37 morphospecies were detected to consist of more than one MOTU by at least one delimitation method. These morphospecies were initially considered potentially to include cryptic species. Since bPTP tends to split MOTUs much more readily than the other methods, only the splits detected by at least two out of three delimitation methods were subjected to further morphological investigation for cryptic diversity. Six morphospecies, *Neoblastobasis biceratala*, *Aroga mesostrepta*, *Evippe albidoesella*, *Faristenia jumbongae*, *Promalactis atriplagata* and *Atrijuglans hetaohei*, fulfilled these conditions.

**Figure 3:**
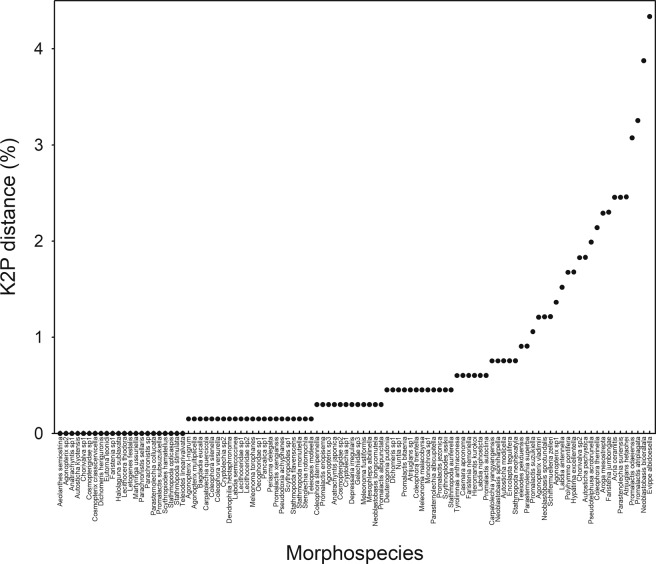
Maximum intraspecific divergences (%) based on Kimura-2-parameter (K2P) for 509 morphospecies.

In the results of the analysis to investigate a threshold for evaluating the number of MOTUs within Gelechioidea, the maximum intraspecific divergence was less than 2.5% in all except four (2.5%) morphospecies (Fig. 3). Of six species to be focused for cryptic diversity, *A. mesostrepta*, *F. jumbongae* and *A. hetaohei*, showed less than 2.5% maximum intraspecific divergence (2.29%, 2.30% and 2.46% respectively), whereas *N. biceratala*, *E. albidoesella* and *P. atriplagata*, showed clearly higher values of maximum intraspecific divergence (3.88%, 4.33% and 3.25% respectively). Next, we present the results of the latter three species in light of subsequent in-depth morphological examination.

#### Species of over 2.5% maximum intraspecific divergence with multiple MOTUs

**Figure 4:**
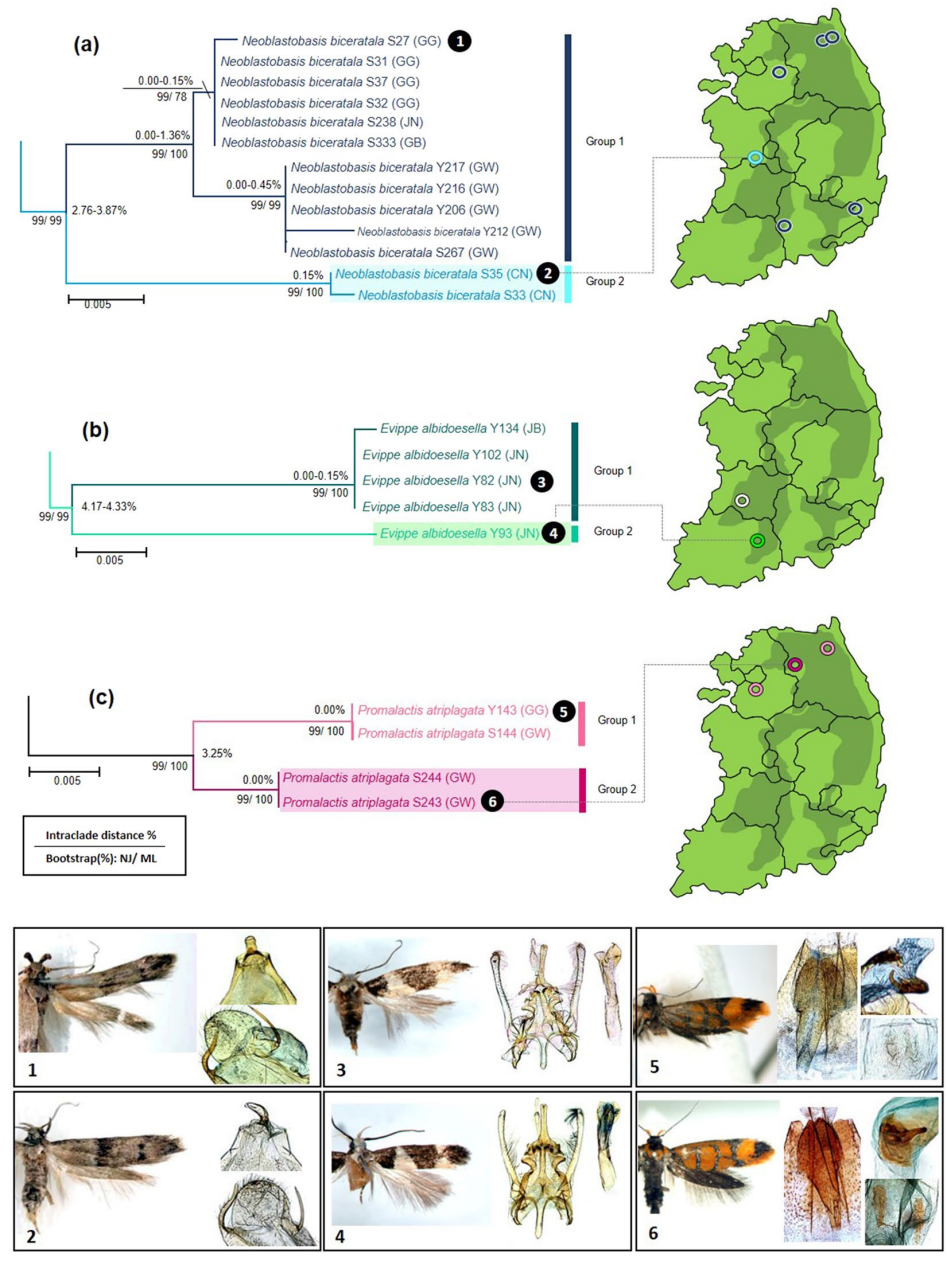
Neighbor-joining tree of COI gene of three morphospecies having higher intraspecific divergence (**a**) *Neoblastobasis biceratala* (Blastobasidae); (**b**) *Evippe albidoesella* (Gelechiidae); (**c**) *Promalactis atriplagata* (Oecophoridae). Numbers on the branches are intraclade distances and numbers under the branches are bootstrap percentages (%) from NJ and ML.

**Figure 5:**
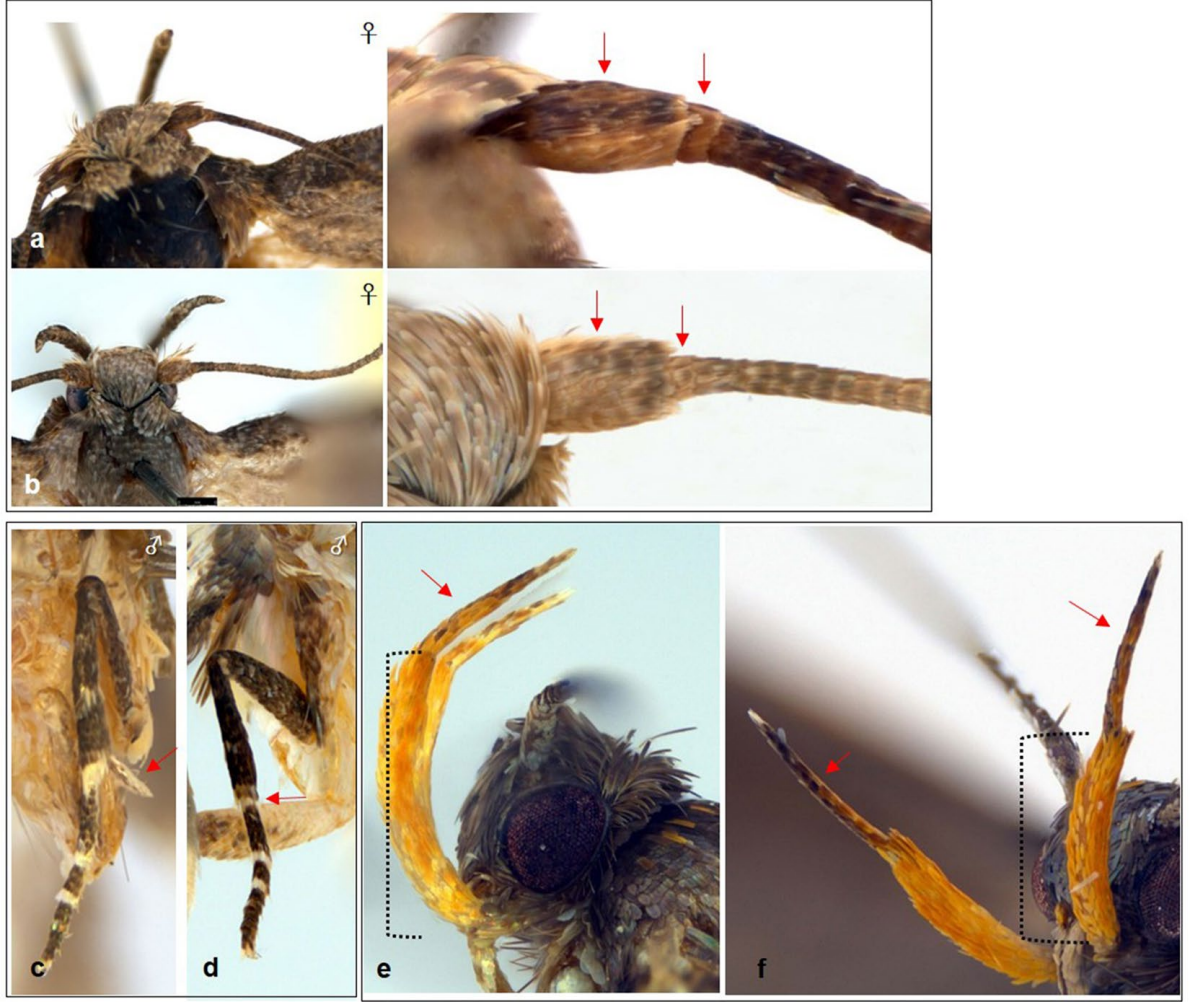
Morphological differences of putative cryptic species. (**a**) *N. biceratala* with group 1 (S333); (**b**) *N. biceratala* with group 2 (S35); (**c**) *E. albidoesella* with group 1(Y82); (**d**) *E. albidoesella* with group 2 (Y93); (**e**) *P. atriplagata* with group 1(Y143); (**f**) *P. atriplagata* with group 2 (S243). The dotted line indicates the 2^nd^ segment of labial palpus.

The 13 analyzed specimens of *N. biceratala* (Blastobasidae) formed three distinct lineages in ML (Fig. 2) and NJ trees (Fig. 4a). The intraclade divergence was less than 2% in each cluster (0.00–0.15, 0.00–0.45 and 0.15, respectively) (Fig. 4a). The group 1 was divided into two subgroups showing a minimum divergence of 2.76 to 3.87% to the group 2, while the subgroups of the group 1 differed only by 0.00–1.36 from each other. The specimens of the group 2 of *N. biceratala* were collected from a very different ecological environment. Specimens of the group 1 were collected from high-altitude mountain areas (Mt. Jungmi, Mt. Nam and Youngdae forest), whereas those of group 2 were collected from low-altitude area, including urban environments (Gongju-si). According to a taxonomic revision of the *Neoblastobasis*^40^, *N. biceratala*, is distinguished from congeneric species by the presence of a developed dorsal plate in valvae of the male genitalia. Those plates were well-observed in both two groups (Figs. 4a, 1–2). However, a minor morphological difference between the two groups was detected in female adults. The scape of antennae and the 1^st^ segment of flagellum are broadly dilated and protruding respectively in the group 1, while the scape of antennae is shorter and the 1^st^ segment of flagellum is not protruding in the group 2 (Fig. 5a-b).

*E. albidoesella* (Gelechiidae) was represented by five specimens collected from two locations. The specimens from these two locations were assigned to different MOTUs and formed two distinct clades in ML (Fig. 2) and NJ trees (Fig. 4c). The second clade was represented by a singular *E. albidoesella*_Y93. The divergence between two clades ranged from 4.17 to 4.33, representing the highest intraspecific divergence within the entire dataset (Fig. 3). In a taxonomic key of genus *Evippe*^41^, *E. albidoesella*, is differentiated by the presence of a white triangular traverse fascia in the forewings and a round apex of cucullus of male genitalia. Those characters were detected in both groups (Figs. 4b, 3–4). Additionally, a distinct difference between the clades was observed: the midleg in the group 1 has a single spur on mid-tibia posteriorly, whereas that is absent in the group 2 (Fig. 5c-d).

**Figure 6:**
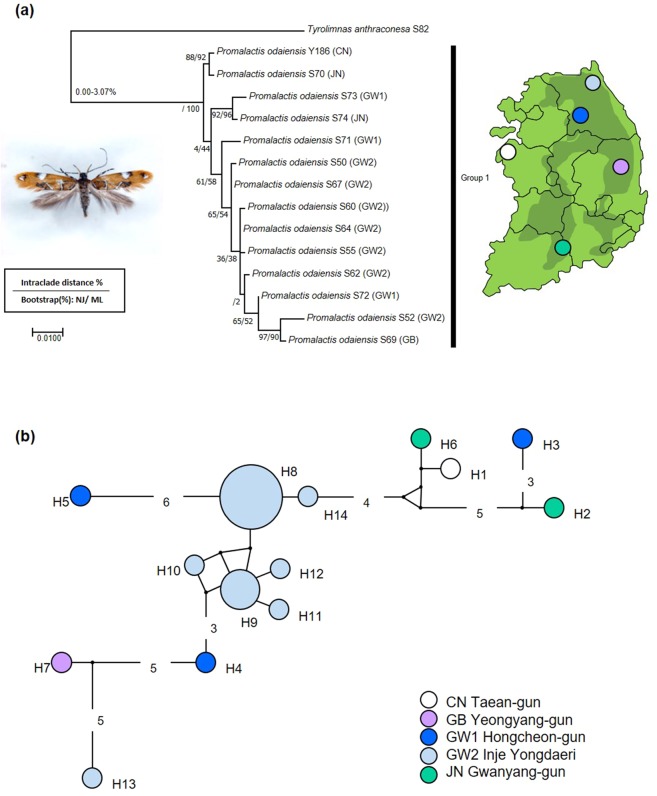
Maximum likelihood tree of COI gene of 14 haplotypes having higher intraspecific divergence and a single MOTU. (**a**) 14 haplotypes of *Promalactis odaiensis* (Oecophoridae). Numbers on the branches are intraclade distances and numbers under the branches are bootstrap percentages (%) from NJ and ML;(**b**) median joining network about 14 haplotypes of 22 COI sequences of *P. odaiensis*. The pie size is proportional to the haplotype frequency (each color indicate the localities).

*P. atriplagata* (Oecophoridae) with four analyzed specimens showed a putative cryptic species as being represented by two MOTUs in the all delimitation methods (Table 3). These splits were also supported by ML and NJ. The interclade divergences between the two groups was 3.25%, whereas no intraclade variability was observed. Moreover, the two specimens of the group 1 were collected from mountain region with natural forests, Mt. Jeombong and Mt. Taehwa, whereas the two specimens of the group 2 were collected from a lowland urban site. *P. atriplagata*, is distinguished from congeneric species by its distinct wing pattern and genitalic characters^42^. Those taxonomic key characters, which are a distinct fuscous apical marking in the wing pattern and the elongated and coiled ductus bursae of female genitalia, were well observed in both groups (Figs. 4c, 5–6). According to an original description and the subsequent revisions^42,43^, the 3^rd^ segment of labial palpus is 2/3 length of the 2^nd^ segment with yellowish orange outer surfaces. These features were found in the specimens of group 1 (Fig. 5e) but, were not present in the group 2. The 3^rd^ segment of labial palpus is almost same length as the 2^nd^ segment and is covered dark brown scales with white apical tips (Fig. 5f).

**Table 3:** Species having higher intraspecific distance and multiple MOTUs.

Family	Species	Max. gen. divergence (%)	MOTUs		
			ABGD	PTP	bPTP
Blastobasidae	*Neoblastobasis biceratala*	3.88	2	2	3
Gelechiidae	*Evippe albidoesella*	4.33	2	2	2
Oecophoridae	*Promalactis atriplagata*	3.25	2	2	2

#### Species of over 2.5% maximum intraspecific divergence with a single MOTU

*Promalactis odaiensis* with maximum intraspecific divergence as high as 3.07% showed only a single MOTU in the all delimitation methods (Table 4). In total, 14 distinct haplotypes (H1-H14), roughly assigned to 5 regions, were observed (Figs. 6a, 6b). No haplotype sharing was present between the regions (Fig. 6b). We could not find any morphological differences between the specimens.

**Table 4:** Species having higher intraspecific divergence but only a single MOTU.

Family	Species	Max. gen. divergence (%)	MOTUs		
			ABGD	PTP	bPTP
Oecophoridae	*Promalactis odaiensis*	3.07	1	1	1

#### Cases with low genetic divergence between morphospecies

**Figure 7:**
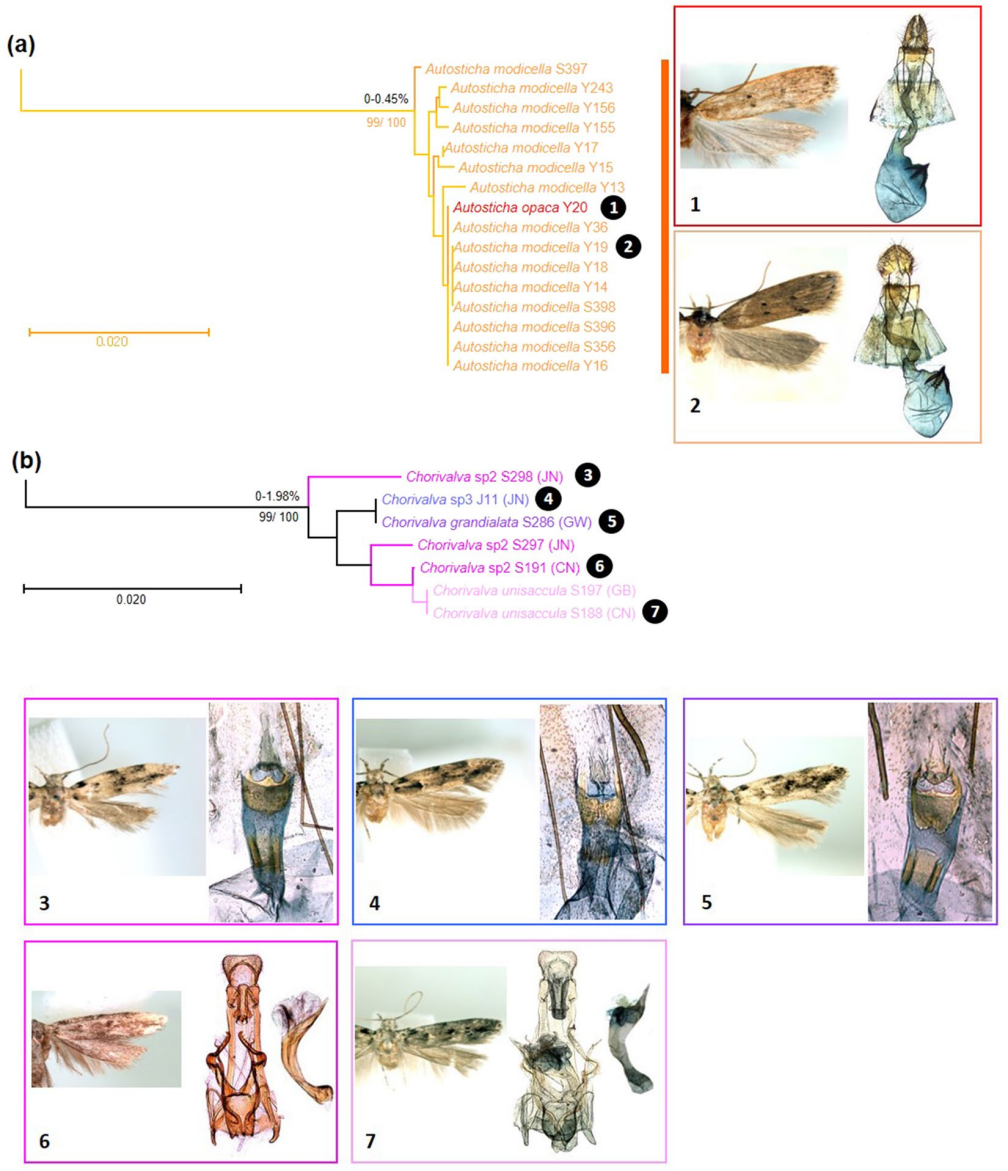
Neighbor-joining tree of COI gene of two groups showing low genetic divergences between morphospecies pair. (**a**) *Autosticha modicella* and *A. opaca*; (**b**) *Chorivalva* complex species. Numbers on the branches are intraclade distances and numbers under the branches are bootstrap percentages (%) from NJ and ML.

Two morphospecies, *Autosticha opaca* and *A. modicella* (Autostichiidae), showed low genetic divergence to each other, ranging from 0.0% to 0.45% (Table 5). In NJ and ML trees (Fig. 7a), the single specimen of *A. opaca* was phylogenetically nested within *A. modicella*. Three species delimitation methods, ABGD, PTP and bPTP, provided the same result.

**Table 5:** Species having low genetic divergence between morphospecies pair.

Family	Species 1	Species 2	Gen. divergence (%)
Autostichiidae	*Autosticha opaca*	*Autosticha modicella*	0–0.45
Gelechiidae	*Chorivalva grandialata*	*Chorivalva unisaccula*	1.21
Gelechiidae	*Chorivalva grandialata*	*Chorivalva* sp.2	1.06–1.98
Gelechiidae	*Chorivalva grandialata*	*Chorivalva* sp.3	0
Gelechiidae	*Chorivalva unisaccula*	*Chorivalva* sp.2	0.15–1.67
Gelechiidae	*Chorivalva unisaccula*	*Chorivalva* sp.3	1.21
Gelechiidae	*Chorivalva* sp.2	*Chorivalva* sp.3	1.06–1.98

Four morphospecies, *Chorivalva grandialata*, *C. unisaccula*, *C*. sp2 and *C*. sp3, altogether including seven specimens (Gelechiidae), were intermixed in NJ and ML trees (Fig. 7b) with low genetic divergence (0.0–1.98%) between the morphospecies. All species delimitation methods assigned these four morphospecies into a single MOTU.

## Discussion

In this study, we tested the utility of DNA barcode in species identification within the Korean Gelechioidea. Our results showed that the species delimitation methods of ABGD, PTP and bPTP yielded 152, 156 and 213 MOTUs, respectively. Number of MOTUs discovered by bPTP was clearly higher than in the other two species delimitation methods. The bPTP method tends to be more sensitive to split sequences into more MOTU than most other delimitation methods. Largely resulting from this difference, only 117 (75.97%) of 154 morphospecies could be identified by each delimitation method. We also investigated the optimal delimitation threshold value for evaluating the number of MOTUs based on maximum intraspecific distance among all 154 morphospecies. The results suggest that the value 2.5% could serve as an efficient proxy for preliminary species delimitation within Gelechioidea. Putative cryptic diversity was detected in three morphospecies (*Neoblastobasis biceratala*, *Evippe albidoesella* and *Promalactis atriplagata*). Each of them is characterized by high intraspecific variability and multiple MOTUs. Ecological differences were also observed in two cases. In *N. biceratala* and *P. atriplagata*, the groups collected from urban sites showed morphological differences to the specimens collected from mountain areas. It can be assumed that geographic isolation with high substitution rate in COI could have led to allopatric speciation without significant morphological differentiation. Of them, *Evippe* is very small and Holarctic genus^41^, most species distributed within Asian area. Only two species, *E. albidoesella* and *E. syrictis*, have been recorded from Korea, of which *E. albidoesella* has frequently been collected and is known from most areas of the Korean Peninsula. Additional taxonomic scrutiny and denser sampling is necessary to further elucidate the taxonomic relationship of these taxa.

Unexpectedly, *Promalactis odaiensis* was observed having high intraspecific variability in COI, albeit under a single MOTU. In the haplotype results, 14 populations were categorized into 5 regions in Korea with the genetic divergence ranged from 0.00 to 3.07%. Given that the *P. odaiensis* is endemic to eastern Asian area^42,44^, the species may show higher genetic diversity in the COI gene. We could suggest that cryptic species should be determined through an integrative analysis comparing the morphology and MOTUs estimation, when the intraspecific maximum genetic divergence exceeds 2.5% in the Gelechioidea.

Our study revealed also cases of mitonuclear discordance, i.e. contradiction between morphology and DNA barcoding. One such group, *Autosticha opaca* and *A. modicella*, showed low intra-genetic divergence and a single MOTU between species in all methods, while being distinguished from each other by morphology. In the taxonomic revision of the group^45^, *A. opaca* could not be distinguished from *A. modicella* by external appearance alone, but only by a very small difference in the genitalia. We could verify this as we observed differences in length and width of ostium bursae and ductus bursae between the two morphospecies (Figs. 7a, 1–2).

Another group of potential cryptic diversity is *Chorivalva* complex of species. *Chorivalva* is a small and a little-known genus of Gelechiidae that is widely distributed in the East Palearctic region. Only three species, distinguished by genital characters, have been recorded^41^. Applying the available taxonomic key, specimens *Chorivalva* were divided into four morphospecies, *Chorivalva unisaccula*, *C. grandialata*, *C*. sp2 and *C*. sp3. (Figs. 7b, 3–7). However, the three delimitation methods, ABGD, PTP and bPTP, all assigned all of them within a single MOTU with low overall divergence. The mitonuclear discordance observed in *Chorivalva* and *Autosticha* could have resulted from incomplete lineage sorting of ancestral mitochondrial DNA polymorphisms, introgression of mitochondrial DNA, possibly mediated by *Wolbachia* infection^46^. Alternatively, the detected differences represent intraspecific variability. Multiple molecular markers, including nuclear ones, and more comprehensive sampling will likely be required to resolve this incongruence between morphology and DNA barcoding.

Some genera of Gelechioidea (e.g. *Parastenolechia*, *Parachronistis*) appear paraphyletic in our trees. Traditionally, the generic classifications of these genera have been inferred based on their morphological similarity, without any rigorous phylogenetic analyses. Our results suggest that many taxonomic discrepancies at species and genus levels should to be re-assessed. It is likely that adoption of multiple genetic markers would likely reveal inconsistencies both in species delimitations and generic classifications.

In conclusion, with this study we demonstrated the usefulness of COI barcode data in efficient species identification in Gelechioidea. Moreover, a functional threshold for tentative species determination within the superfamily was proposed. We demonstrated that DNA barcoding provides an efficient way to detect morphologically cryptic species. Comprehensive DNA barcode reference libraries would also facilitate accurate identification of immature stages of pests, which are many in Gelechioidea.

**Accession Codes:** From MK210635 to MK211143 for COI sequences.

## Supplementary information

Supplementary dataset.
