# Supplementary material for: High functionality of DNA barcodes and revealed cases of cryptic diversity in Korean curved-horn moths (Lepidoptera: Gelechioidea)
Source: Sci Rep. 2020 Apr 10;10:6208. doi: 10.1038/s41598-020-63385-x (PMC7148304; doi:10.1038/s41598-020-63385-x)
Supplement: Supplementary file 1 — Supplementary dataset. [file 41598_2020_63385_MOESM1_ESM.pdf]

High functionality of DNA barcodes and revealed cases of cryptic diversity in Korean curved-horn moths (Lepidoptera: Gelechioidea)

Sora Kim[1], Yerim Lee[1], Marko Mutanen[2], Jinbae Seung[1] & Seunghwan Lee[1]

[1]Laboratory of Insect Biosystematics, Department of Agricultural Biotechnology, Research Institute of Agriculture and Life Sciences, Seoul National University, Seoul, 08826, Republic of Korea.

[2]Ecology and Genetics Research Unit, PO Box 3000, FI-90014, University of Oulu, Oulu, Finland

*Correspondence and requests for materials should be addressed to S.K. (email: s.kim.microlepi@gmail.com) or S.L. (seung@snu.ac.kr)

Table S1. A total of 509 sequences for 154 morphospecies were generated as novel data in the present study including Genbank Accession Number and GPS information.

No.	Family/Subfamily	Scientific name	Locality	GPS			Collector	Date	Genbank accession No.
Y138	Depressariidae	*Acria ceramitis*	Daepo-dong, Seogwipo-si, Is. Jeju, Korea	33.307359	(33° 18' 26.49" N)	126.4546002	S.Y.Park, J.S.Lim	130904	MK211139
S321	Depressariidae	*Acria ceramitis*	Korea National Arboretum, Jikdong-ri, Soheul-eup, Pocheon-si, Gyeonggi-do, Korea	37.7568443	(37° 45' 24.64" N)	127.1678772	S.J.Roh, Y.M.Shin	170720	MK211143
S324	Depressariidae	*Acria ceramitis*	Mt. Toham, Hwangyong-dong, Gyeongju-si, Gyeongsangbuk-do, Korea	35.8232501	(35° 49' 23.70" N)	129.3685615	Park, Shin, Nam, Kim	160901	MK211140
S322	Depressariidae	*Acria ceramitis*	Trail B, Jincheon-ri, Beakryeong-myeon, Ongjin-gun, Incheon-si, Gyeonggi-do, Korea	37.9749722	(37° 58' 29.90" N)	124.7043481	Park, Shin, Nam	150706	MK211142
S323	Depressariidae	*Acria ceramitis*	Trail B, Jincheon-ri, Beakryeong-myeon, Ongjin-gun, Incheon-si, Gyeonggi-do, Korea	37.9749722	(37° 58' 29.90" N)	124.7043481	Park, Shin, Nam	150909	MK211141
Y146	Depressariidae	*Acria* sp1	Chudong-ri, Hajang-myeon, Samcheok-si, Gangwon-do, Korea	37.344109	(37° 20' 38.79" N)	128.940787	Park, Lim, Kim	90831	MK211138
Y148	Aeolanthinae	*Aeolanthes semiostrina*	Mt. Daeso, Songhyeon-ri, Sowon-myeon, Taean-gun, Chungcheongnam-do, Korea	36.7731411	(36° 46' 23.31" N)	126.1794459	Park, Shin, Kim	150610	MK211137
Y149	Aeolanthinae	*Aeolanthes semiostrina*	Mt. Daeso, Songhyeon-ri, Sowon-myeon, Taean-gun, Chungcheongnam-do, Korea	36.7731411	(36° 46' 23.31" N)	126.1794459	Park, Shin, Kim	150610	MK211136
Y150	Aeolanthinae	*Aeolanthes semiostrina*	Trail B, Jincheon-ri, Beakryeong-myeon, Ongjin-gun, Incheon-si, Gyeonggi-do, Korea	37.9749722	(37° 58' 29.90" N)	124.7043481	Park, Shin, Nam	150706	MK211135
Y151	Aeolanthinae	*Aeolanthes semiostrina*	Trail B, Jincheon-ri, Beakryeong-myeon, Ongjin-gun, Incheon-si, Gyeonggi-do, Korea	37.9749722	(37° 58' 29.90" N)	124.7043481	Park, Shin, Nam	150706	MK211134
S21	Depressariidae	*Agonopterix l-nigrum*	Jungsan-ri, Daedeok-myeon, Gimcheon-si, Gyeongsangbuk-do, Korea	35.9358889	(35° 56' 9.20" N)	127.9907773	S.R.Kim	120723	MK211133
S352	Depressariidae	*Agonopterix l-nigrum*	Mt. Geumo, Yeulim-ri, Dolsan-eup, Yeosu-gun, Jeollanam-do, Korea	34.5921879	(34° 35' 31.88" N)	127.8015618	Lim, Choi, Lee, Roh	170612	MK211132
S358	Depressariidae	*Agonopterix l-nigrum*	Mt. Geumo, Yeulim-ri, Dolsan-eup, Yeosu-gun, Jeollanam-do, Korea	34.5921879	(34° 35' 31.88" N)	127.8015618	Lim, Choi, Lee, Roh	170612	MK211131
S18	Depressariidae	*Agonopterix multiplicella*	Ara-dong, Jeju-si, Is. Jeju, Korea	33.4261448	(33° 25' 34.12" N)	126.5586626	Park, Kim, Lee	140526	MK211130
Y44	Depressariidae	*Agonopterix multiplicella*	Ara-dong, Jeju-si, Is. Jeju, Korea	33.4261448	(33° 25' 34.12" N)	126.5586626	Park, Kim, Lee	140911	MK211126
Y45	Depressariidae	*Agonopterix multiplicella*	Ara-dong, Jeju-si, Is. Jeju, Korea	33.4261448	(33° 25' 34.12" N)	126.5586626	Park, Kim, Lee	140911	MK211125
S19	Depressariidae	*Agonopterix multiplicella*	Hannam-ri, Namwon-eup, Seogwipo-si, Is. Jeju, Korea	33.3318179	(33° 19' 54.54" N)	126.6805574	Park, Kim, Lee	140526	MK211129
S20	Depressariidae	*Agonopterix multiplicella*	Hannam-ri, Namwon-eup, Seogwipo-si, Is. Jeju, Korea	33.3318179	(33° 19' 54.54" N)	126.6805574	Park, Kim, Lee	140526	MK211128
Y38	Depressariidae	*Agonopterix multiplicella*	Harye-ri, Namwon-eup, Seogwipo-si, Is. Jeju, Korea	33.3252568	(33° 19' 30.92" N)	126.6020681	Park, Kim, Lee	140526	MK211127
Y42	Depressariidae	*Agonopterix* sp1	Harye-ri, Namwon-eup, Seogwipo-si, Is. Jeju, Korea	33.3252568	(33° 19' 30.92" N)	126.6020681	Park, Kim, Lee	140611	MK211121
S13	Depressariidae	*Agonopterix* sp1	Muk-ri, Chuja-myeon, Is. Jeju, Korea	33.9500756	(33° 57' 0.27" N)	126.3083811	S.Y.Park, J.S.Lim	131017	MK211124
S15	Depressariidae	*Agonopterix* sp1	Muk-ri, Chuja-myeon, Is. Jeju, Korea	33.9500756	(33° 57' 0.27" N)	126.3083811	S.Y.Park, J.S.Lim	131017	MK211122
S14	Depressariidae	*Agonopterix* sp1	Sangchang-ri, Andeok-myeon, Seogwipo-si, Is. Jeju, Korea	33.2882178	(33° 17' 17.58" N)	126.3588619	Park, Kim, Lee	140410	MK211123
Y160	Depressariidae	*Agonopterix* sp2	Trail g, Jincheon-ri, Beakryeong-myeon, Ongjin-gun, Incheon-si, Gyeonggi-do, Korea	37.9522354	(37° 57' 8.05" N)	124.718596	Park, Shin, Kim	150513	MK211120
Y161	Depressariidae	*Agonopterix* sp2	Trail g, Jincheon-ri, Beakryeong-myeon, Ongjin-gun, Incheon-si, Gyeonggi-do, Korea	37.9522354	(37° 57' 8.05" N)	124.718596	Park, Shin, Kim	150513	MK211119
S325	Depressariidae	*Agonopterix* sp3	Mt. Geumo, Yeulim-ri, Dolsan-eup, Yeosu-gun, Jeollanam-do, Korea	34.5921879	(34° 35' 31.88" N)	127.8015618	Lim, Choi, Lee, Roh	170612	MK211118
S326	Depressariidae	*Agonopterix* sp3	Mt. Geumo, Yeulim-ri, Dolsan-eup, Yeosu-gun, Jeollanam-do, Korea	34.5921879	(34° 35' 31.88" N)	127.8015618	Lim, Choi, Lee, Roh	170612	MK211117
S327	Depressariidae	*Agonopterix* sp3	Mt. Geumo, Yeulim-ri, Dolsan-eup, Yeosu-gun, Jeollanam-do, Korea	34.5921879	(34° 35' 31.88" N)	127.8015618	Lim, Choi, Lee, Roh	170612	MK211116
S10	Depressariidae	*Agonopterix vladimiri*	Gapa-ri, Daejeong-eup, Seogwipo-si, Is. Jeju, Korea	33.1697665	(33° 10' 11.16" N)	126.2740071	S.Y.Park, J.S.Lim	140730	MK211115
S7	Depressariidae	*Agonopterix vladimiri*	Gapa-ri, Daejeong-eup, Seogwipo-si, Is. Jeju, Korea	33.1697665	(33° 10' 11.16" N)	126.2740071	S.Y.Park, J.S.Lim	140730	MK211112
S8	Depressariidae	*Agonopterix vladimiri*	Gapa-ri, Daejeong-eup, Seogwipo-si, Is. Jeju, Korea	33.1697665	(33° 10' 11.16" N)	126.2740071	S.Y.Park, J.S.Lim	140730	MK211111
S6	Depressariidae	*Agonopterix vladimiri*	Gapa-ri, Daejeong-eup, Seogwipo-si, Is. Jeju, Korea	33.1697665	(33° 10' 11.16" N)	126.2740071	S.Y.Park, J.S.Lim	150729	MK211113
S9	Depressariidae	*Agonopterix vladimiri*	Gapa-ri, Daejeong-eup, Seogwipo-si, Is. Jeju, Korea	33.1697665	(33° 10' 11.16" N)	126.2740071	S.Y.Park, J.S.Lim	150729	MK211110
S11	Depressariidae	*Agonopterix vladimiri*	Yeonpyeong-ri, Udo-myeon, Jeju-si, Is. Jeju, Korea	33.4978923	(33° 29' 52.41" N)	126.9640902	Park, Lim, Oh, Go	130826	MK211114
Y162	Depressariidae	*Agonopterix yamatoensis*	Sogwang-ri, Geumgangsong-myeon, Uljin-gun, Gyeongsangbuk-do, Korea	37.0157781	(37° 0' 56.80" N)	129.2136744	Nam, Kim, Kim, Park	160706	MK211109
S411	Gelechiidae	*Anacampsis anisogramma*	Korea National Arboretum, Jikdong-ri, Soheul-eup, Pocheon-si, Gyeonggi-do, Korea	37.7568443	(37° 45' 24.64" N)	127.1678772	S.J.Roh, Y.M.Shin	170807	MK211108
S224	Gelechiidae	*Anacampsis* sp	Cheongpodae Beach, Woncheong-ri, Nam-myeon, Taean-gun, Chungcheongnam-do, Korea	36.6402712	(36° 38' 24.98" N)	126.3014771	Park, Shin, Kim	150827	MK211107
Y196	Cosmopterigidae	*Anatrachyntis japonica*	Hwadong-salt Farm-Opposite, Nampo-ri, Beakryeong-myeon, Ongjin-gun, Incheon-si, Gyeonggi-do, Korea	37.9269189	(37° 55' 36.91" N)	124.69937	Park, Shin, Nam	150907	MK211104
Y195	Cosmopterigidae	*Anatrachyntis japonica*	Seungeon-ri, Anmyeong-eup, Taean-gun, Chungcheongnam-do, Korea	36.4999659	(36° 29' 59.88" N)	126.3621608	Park, Shin, Kim, Nam	150826	MK211105
Y197	Cosmopterigidae	*Anatrachyntis japonica*	Seungeon-ri, Anmyeong-eup, Taean-gun, Chungcheongnam-do, Korea	36.4999659	(36° 29' 59.88" N)	126.3621608	Park, Shin, Kim, Nam	150826	MK211103
S418	Cosmopterigidae	*Anatrachyntis japonica*	Yonggang-dong, Gyeongju-si, Gyeongsangbuk-do, Korea	35.8697361	(35° 52' 11.05" N)	129.2367455	Park, Shin, Nam, Kim	160613	MK211106
Y198	Cosmopterigidae	*Anatrachyntis* sp1	Hwadong-salt Farm-Opposite, Nampo-ri, Beakryeong-myeon, Ongjin-gun, Incheon-si, Gyeonggi-do, Korea	37.9269189	(37° 55' 36.91" N)	124.69937	Park, Shin, Nam	150907	MK211101
Y199	Cosmopterigidae	*Anatrachyntis* sp1	Hwadong-salt Farm-Opposite, Nampo-ri, Beakryeong-myeon, Ongjin-gun, Incheon-si, Gyeonggi-do, Korea	37.9269189	(37° 55' 36.91" N)	124.69937	Park, Shin, Nam	150907	MK211100
Y201	Cosmopterigidae	*Anatrachyntis* sp1	Hwadong-salt Farm-Opposite, Nampo-ri, Beakryeong-myeon, Ongjin-gun, Incheon-si, Gyeonggi-do, Korea	37.9269189	(37° 55' 36.91" N)	124.69937	Park, Shin, Nam	150907	MK211102
S229	Gelechiidae	*Aroga mesostrepta*	Mt. Daeso, Songhyeon-ri, Sowon-myeon, Taean-gun, Chungcheongnam-do, Korea	36.7731411	(36° 46' 23.31" N)	126.1794459	Park, Shin, Kim	150610	MK211099
Y202	Gelechiidae	*Aroga mesostrepta*	Mt. Daeso, Songhyeon-ri, Sowon-myeon, Taean-gun, Chungcheongnam-do, Korea	36.7731411	(36° 46' 23.31" N)	126.1794459	Park, Shin, Kim	150610	MK211098
S245	Stathmopodidae	*Atrijuglans hetaohei*	Mt. Taehwa, Sangrim-ri, Docheok-myeon, Gwangju-si, Gyeonggi-do, Korea	37.3057197	(37° 18' 20.59" N)	127.3028244	S.R.Kim	130615	MK210681
S152	Stathmopodidae	*Atrijuglans hetaohei*	Taeha-ri, Seo-myeon, Ulleung-gun, Gyeongsangbuk-do, Korea	37.5050062	(37° 30' 18.02" N)	130.8277999	Park, Shin, Nam	140807	MK210682
S337	Stathmopodidae	*Atrijuglans* sp1	Korea National Arboretum, Jikdong-ri, Soheul-eup, Pocheon-si, Gyeonggi-do, Korea	37.7568443	(37° 45' 24.64" N)	127.1678772	S.J.Roh, Y.M.Shin	170807	MK211097
S338	Stathmopodidae	*Atrijuglans* sp1	Korea National Arboretum, Jikdong-ri, Soheul-eup, Pocheon-si, Gyeonggi-do, Korea	37.7568443	(37° 45' 24.64" N)	127.1678772	S.J.Roh, Y.M.Shin	170807	MK211096
S339	Stathmopodidae	*Atrijuglans* sp1	Korea National Arboretum, Jikdong-ri, Soheul-eup, Pocheon-si, Gyeonggi-do, Korea	37.7568443	(37° 45' 24.64" N)	127.1678772	S.J.Roh, Y.M.Shin	170807	MK211095
S340	Stathmopodidae	*Atrijuglans* sp1	Korea National Arboretum, Jikdong-ri, Soheul-eup, Pocheon-si, Gyeonggi-do, Korea	37.7568443	(37° 45' 24.64" N)	127.1678772	S.J.Roh, Y.M.Shin	170807	MK211094
Y3	Autostichiidae	*Autosticha kyotensis*	Gamsan-ri, Andeok-myeon, Seogwipo-si, Is. Jeju, Korea	33.2544023	(33° 15' 15.85" N)	126.3604401	Park, Kim, Lee	120726	MK211092
Y2	Autostichiidae	*Autosticha kyotensis*	Gamsan-ri, Andeok-myeon, Seogwipo-si, Is. Jeju, Korea	33.2544023	(33° 15' 15.85" N)	126.3604401	Park, Kim, Lee	140724	MK211093
Y4	Autostichiidae	*Autosticha kyotensis*	Hwasun-ri, Andeok-myeon, Seogwipo-si, Is. Jeju, Korea	33.2656814	(33° 15' 56.45" N)	126.3340093	Park, Kim, Lee	140611	MK211091
Y16	Autostichiidae	*Autosticha modicella*	Changcheon-ri, Andeok-myeon, Seogwipo-si, Is. Jeju, Korea	33.2474936	(33° 14' 50.98" N)	126.3655928	Park, Kim, Lee	140630	MK211090
Y18	Autostichiidae	*Autosticha modicella*	Changcheon-ri, Andeok-myeon, Seogwipo-si, Is. Jeju, Korea	33.2474936	(33° 14' 50.98" N)	126.3655928	Park, Kim, Lee	140630	MK211079
S397	Autostichiidae	*Autosticha modicella*	Geumbong-ri, Dolsan-eup, Yeosu-gun, Jeollanam-do, Korea	34.6431937	(34° 38' 35.50" N)	127.7493644	Lim, Choi, Lee, Roh	170612	MK211087
Y19	Autostichiidae	*Autosticha modicella*	Harye-ri, Namwon-eup, Seogwipo-si, Is. Jeju, Korea	33.3252568	(33° 19' 30.92" N)	126.6020681	Park, Kim, Lee	140710	MK211078
Y15	Autostichiidae	*Autosticha modicella*	Hwasun-ri, Andeok-myeon, Seogwipo-si, Is. Jeju, Korea	33.2656814	(33° 15' 56.45" N)	126.3340093	Park, Kim, Lee	140630	MK211083
S398	Autostichiidae	*Autosticha modicella*	Mt. Daeso, Songhyeon-ri, Sowon-myeon, Taean-gun, Chungcheongnam-do, Korea	36.7731411	(36° 46' 23.31" N)	126.1794459	Park, Shin, Kim	150610	MK211086
Y13	Autostichiidae	*Autosticha modicella*	Mt. Deokyoo, Samgong-ri, Seolcheon-myeon, Muju-gun, Jeollabuk-do, Korea	35.860082	(35° 51' 36.30" N)	127.76631	Y.M.Park, H.K.Lee	100819	MK211085
Y156	Autostichiidae	*Autosticha modicella*	Mt. Dondae, Yecho-ri, Chuja-myeon, Is. Jeju, Korea	33.9454787	(33° 56' 43.72" N)	126.3250536	S.Y.Park, J.S.Lim	130701	MK211081
Y243	Autostichiidae	*Autosticha modicella*	Mureung-ri, Daejeong-eup, Seogwipo-si, Is. Jeju, Korea	33.278215	(33° 16' 41.57" N)	126.25147	Park, Kim, Lee	170521	MK211077
Y17	Autostichiidae	*Autosticha modicella*	Sanghyo-dong, Seogwipo-si, Is. Jeju, Korea	33.3012848	(33° 18' 4.63" N)	126.5816049	Park, Lim, Oh, Go	130901	MK211080
Y14	Autostichiidae	*Autosticha modicella*	Yeonpyeong-ri, Udo-myeon, Jeju-si, Is. Jeju, Korea	33.4935619	(33° 29' 36.82" N)	126.9587687	Y.M. Park	100907	MK211084
Y36	Autostichiidae	*Autosticha modicella*	Yeonpyeong-ri, Udo-myeon, Jeju-si, Is. Jeju, Korea	33.4935619	(33° 29' 36.82" N)	126.9587687	Y.M. Park	100907	MK211076
S396	Autostichiidae	*Autosticha modicella*	Yonggang-dong, Gyeongju-si, Gyeongsangbuk-do, Korea	35.8697361	(35° 52' 11.05" N)	129.2367455	Park, Shin, Nam, Kim	160613	MK211088
Y155	Autostichiidae	*Autosticha modicella*	Youngheung-ri, Chuja-myeon, Is. Jeju, Korea	33.9580095	(33° 57' 28.83" N)	126.2966651	Park, Lim, Oh, Go	130830	MK211082

S356	Autostichiidae	*Autosticha modicella*	Yuklim lake, Jikdong-ri, Soheul-eup, Pocheon-si, Gyeonggi-do, Korea	37.748548	(37° 44' 54.77" N)	127.1651502	J.S. Lim	170627	MK211089
Y20	Autostichiidae	*Autosticha opaca*	Hannam-ri, Namwon-eup, Seogwipo-si, Is. Jeju, Korea	33.3318179	(33° 19' 54.54" N)	126.6805574	Park, Kim, Lee	140710	MK211075
J41	Autostichiidae	*Autosticha pachystica*	Ara-dong, Jeju-si, Is. Jeju, Korea	33.4261448	(33° 25' 34.12" N)	126.5586626	Park, Kim, Lee	140710	MK211073
Y177	Autostichiidae	*Autosticha pachystica*	Byungnae-ri Daegwallyeong-myeon, Pyeongchang-gun, Gangwon-do, Korea	37.7174852	(37° 43' 2.95" N)	128.6275487	Y.R. Lee	130814	MK211071
J39	Autostichiidae	*Autosticha pachystica*	Gamsan-ri, Andeok-myeon, Seogwipo-si, Is. Jeju, Korea	33.2544023	(33° 15' 15.85" N)	126.3604401	Park, Kim, Lee	140724	MK211074
Y21	Autostichiidae	*Autosticha pachystica*	Napeup-ri, Aewol-eup, Jeju-si, Is. Jeju, Korea	33.4279367	(33° 25' 40.57" N)	126.3402467	Park, Kim, Lee	140724	MK211070
J48	Autostichiidae	*Autosticha pachystica*	Seungeon-ri, Anmyeong-eup, Taean-gun, Chungcheongnam-do, Korea	36.4999659	(36° 29' 59.88" N)	126.3621608	Park, Shin, Kim, Nam	150826	MK211072
Y229	Autostichiidae	*Autosticha pachystica*	Seungeon-ri, Anmyeong-eup, Taean-gun, Chungcheongnam-do, Korea	36.4999659	(36° 29' 59.88" N)	126.3621608	Park, Shin, Kim, Nam	150826	MK211069
Y230	Autostichiidae	*Autosticha pachystica*	Seungeon-ri, Anmyeong-eup, Taean-gun, Chungcheongnam-do, Korea	36.4999659	(36° 29' 59.88" N)	126.3621608	Park, Shin, Kim, Nam	150826	MK211068
Y233	Autostichiidae	*Autosticha pachystica*	Seungeon-ri, Anmyeong-eup, Taean-gun, Chungcheongnam-do, Korea	36.4999659	(36° 29' 59.88" N)	126.3621608	Park, Shin, Kim, Nam	150826	MK211067
Y1	Autostichiidae	*Autosticha trincicola*	Harye-ri, Namwon-eup, Seogwipo-si, Is. Jeju, Korea	33.3252568	(33° 19' 30.92" N)	126.6020681	Park, Kim, Lee	140710	MK211066
S184	Gelechiidae	*Bagdadia eucalla*	Mt. Gyeryongsan, Hakbong-ri, Banpo-myeon, Gongju-si, Chungcheongnam-do, Korea	36.353291	(36° 21' 11.85" N)	127.2559724	S.R.Kim	120623	MK211065
S192	Gelechiidae	*Bagdadia eucalla*	Mt. Gyeryongsan, Hakbong-ri, Banpo-myeon, Gongju-si, Chungcheongnam-do, Korea	36.353291	(36° 21' 11.85" N)	127.2559724	S.R.Kim	120624	MK211064
S390	Batrachedridae	*Batrachedra albicapitella*	Mt. Danseok, Hwacheon-ri, Geoncheon-eup, Gyeongju-si, Gyeongsangbuk-do, Korea	35.788845	(35° 47' 19.84" N)	129.114107	Park, Shin, Kim, Nam	160615	MK211063
Y112	Blastobasidae	*Blastobasis spiniella*	Chusan-ri, Okryong-myeon, Gwangyang-si, Jeollanam-do, Korea	35.0491591	(35° 2' 56.97" N)	127.59797	Y.R. Lee	130726	MK211062
Y130	Gelechiidae	*Carpatolechia daehania*	Mt. Palgong, Dongsan-ri, Bugye-myeon, Gunwi-gun, Gyeongsangbuk-do, Korea	35.9726393	(35° 58' 21.50" N)	128.6636718	Park, Shin, Kim, Nam	140701	MK211061
Y163	Gelechiidae	*Carpatolechia quercicola*	Yuklim lake, Jikdong-ri, Soheul-eup, Pocheon-si, Gyeonggi-do, Korea	37.748548	(37° 44' 54.77" N)	127.1651502	Park, Nan, Shin, Kim, Son	150528	MK211060
Y164	Gelechiidae	*Carpatolechia quercicola*	Yuklim lake, Jikdong-ri, Soheul-eup, Pocheon-si, Gyeonggi-do, Korea	37.748548	(37° 44' 54.77" N)	127.1651502	Park, Nan, Shin, Kim, Son	150528	MK211059
S375	Gelechiidae	*Carpatolechia yangyangensis*	Mt. Gyeryongsan, Hakbong-ri, Banpo-myeon, Gongju-si, Chungcheongnam-do, Korea	36.3573002	(36° 21' 26.28" N)	127.2423253	S.R.Kim	120724	MK211054
J12	Gelechiidae	*Carpatolechia yangyangensis*	Mt. Gyeryongsan, Hakbong-ri, Banpo-myeon, Gongju-si, Chungcheongnam-do, Korea	36.3573002	(36° 21' 26.28" N)	127.2423253	S.R.Kim	120724	MK211058
J13	Gelechiidae	*Carpatolechia yangyangensis*	Mt. Gyeryongsan, Hakbong-ri, Banpo-myeon, Gongju-si, Chungcheongnam-do, Korea	36.3573002	(36° 21' 26.28" N)	127.2423253	S.R.Kim	120724	MK211057
J14	Gelechiidae	*Carpatolechia yangyangensis*	Mt. Gyeryongsan, Hakbong-ri, Banpo-myeon, Gongju-si, Chungcheongnam-do, Korea	36.3573002	(36° 21' 26.28" N)	127.2423253	S.R.Kim	120724	MK211056
J15	Gelechiidae	*Carpatolechia yangyangensis*	Mt. Gyeryongsan, Hakbong-ri, Banpo-myeon, Gongju-si, Chungcheongnam-do, Korea	36.3573002	(36° 21' 26.28" N)	127.2423253	S.R.Kim	120724	MK211055
Y215	Gelechiidae	*Carpatolechia yangyangensis*	Sogwang-ri, Geumgangsong-myeon, Uljin-gun, Gyeongsangbuk-do, Korea	37.0157781	(37° 0' 56.80" N)	129.2136744	Nam, Kim, Kim, Park	160706	MK211053
Y225	Gelechiidae	*Carpatolechia yangyangensis*	Sogwang-ri, Geumgangsong-myeon, Uljin-gun, Gyeongsangbuk-do, Korea	37.0157781	(37° 0' 56.80" N)	129.2136744	Nam, Kim, Kim, Park	160706	MK211052
Y136	Stathmopodidae	*Casmara apronoma*	Bugok lake, Bugok-ri, Gangrim-myeon, Hoengseong-gun, Gangwon-do, Korea	37.3288309	(37° 19' 43.79" N)	128.0659713	S.W. Park	130806	MK211050
Y137	Stathmopodidae	*Casmara apronoma*	Icheon-ri, Sangbuk-myeon, Ulju-gun, Ulsan-si, Gyeongsangnam-do, Korea	35.5453457	(35° 32' 43.24" N)	129.0250682	S.W. Park	140807	MK211049
Y135	Stathmopodidae	*Casmara apronoma*	Mt. Ilwol, Galsan-ri, Jaesan-myeon, Bonghwa-gun, Gyeongsangbuk-do, Korea	36.788278	(36° 47' 17.80" N)	129.0636394	S.W.Park	140729	MK211051
S286	Gelechiidae	*Chorivalva grandialata*	Yongdae recreation forest, Yongdae-ri, Buk-myeon, Inje-gun, Gangwon-do, Korea	38.2360777	(38° 14' 9.88" N)	128.3449028	Y.R. Lee	130731	MK211048
S297	Gelechiidae	*Chorivalva* sp2	Chusan-ri, Okryong-myeon, Gwangyang-si, Jeollanam-do, Korea	35.0467525	(35° 2' 48.31" N)	127.589623	Y.R. Lee	160618	MK211044
S298	Gelechiidae	*Chorivalva* sp2	Chusan-ri, Okryong-myeon, Gwangyang-si, Jeollanam-do, Korea	35.0467525	(35° 2' 48.31" N)	127.589623	Y.R. Lee	160618	MK211043
S191	Gelechiidae	*Chorivalva* sp2	Mt. Gyeryongsan, Hakbong-ri, Banpo-myeon, Gongju-si, Chungcheongnam-do, Korea	36.353291	(36° 21' 11.85" N)	127.2559724	S.R.Kim	120724	MK211045
J11	Gelechiidae	*Chorivalva* sp3	Mt. Gyeryongsan, Hakbong-ri, Banpo-myeon, Gongju-si, Chungcheongnam-do, Korea	36.353291	(36° 21' 11.85" N)	127.2559724	S.R.Kim	120724	MK211042
S188	Gelechiidae	*Chorivalva unisaccula*	Mt. Gyeryongsan, Hakbong-ri, Banpo-myeon, Gongju-si, Chungcheongnam-do, Korea	36.353291	(36° 21' 11.85" N)	127.2559724	S.R.Kim	120724	MK211047
S197	Gelechiidae	*Chorivalva unisaccula*	Mt. Palgong, Dongsan-ri, Bugye-myeon, Gunwi-gun, Gyeongsangbuk-do, Korea	35.9726393	(35° 58' 21.50" N)	128.6636718	Park, Shin, Kim, Nam	140701	MK211046
S386	Coleophoridae	*Coleophora chenopodii*	Hwadong-salt Farm-Opposite, Nampo-ri, Beakryeong-myeon, Ongjin-gun, Incheon-si, Gyeonggi-do, Korea	37.9269189	(37° 55' 36.91" N)	124.69937	Park, Shin, Nam	150907	MK211041
S216	Coleophoridae	*Coleophora silenella*	Hwadong-salt Farm-Opposite, Nampo-ri, Beakryeong-myeon, Ongjin-gun, Incheon-si, Gyeonggi-do, Korea	37.9269189	(37° 55' 36.91" N)	124.69937	Park, Shin, Nam	150907	MK211040
Y219	Coleophoridae	*Coleophora silenella*	Hwadong-salt Farm-Opposite, Nampo-ri, Beakryeong-myeon, Ongjin-gun, Incheon-si, Gyeonggi-do, Korea	37.9269189	(37° 55' 36.91" N)	124.69937	Park, Shin, Nam	150907	MK211039
S387	Coleophoridae	*Coleophora* sp1	Mt. Gumi, Gajeong-ri, Hyeongok-myeon, Gyeongju-si, Gyeongsangbuk-do, Korea	35.9006093	(35° 54' 2.19" N)	129.1480005	Park, Shin, Kim, Nam	160616	MK211038
S392	Coleophoridae	*Coleophora sternipennella*	Mt. Jukyeop, Komo-ri, Soheul-eup, Pocheon-si, Gyeonggi-do, Korea	37.7931837	(37° 47' 35.46" N)	127.1699635	B.W.Lee	130827	MK211037
S393	Coleophoridae	*Coleophora sternipennella*	Mt. Jukyeop, Komo-ri, Soheul-eup, Pocheon-si, Gyeonggi-do, Korea	37.7931837	(37° 47' 35.46" N)	127.1699635	B.W.Lee	130827	MK211036
Y188	Coleophoridae	*Coleophora therinella*	Duwoong-wetland, Sindu-ri, Wonbuk-myeon, Taean-gun, Chungcheongnam-do, Korea	36.835705	(36° 50' 8.54" N)	126.1956258	Park, Shin, Kim, Nam	150824	MK211029
S213	Coleophoridae	*Coleophora therinella*	Hwadong-salt Farm-Opposite, Nampo-ri, Beakryeong-myeon, Ongjin-gun, Incheon-si, Gyeonggi-do, Korea	37.9269189	(37° 55' 36.91" N)	124.69937	Park, Shin, Nam	150907	MK211035
S214	Coleophoridae	*Coleophora therinella*	Hwadong-salt Farm-Opposite, Nampo-ri, Beakryeong-myeon, Ongjin-gun, Incheon-si, Gyeonggi-do, Korea	37.9269189	(37° 55' 36.91" N)	124.69937	Park, Shin, Nam	150907	MK211034
S215	Coleophoridae	*Coleophora therinella*	Hwadong-salt Farm-Opposite, Nampo-ri, Beakryeong-myeon, Ongjin-gun, Incheon-si, Gyeonggi-do, Korea	37.9269189	(37° 55' 36.91" N)	124.69937	Park, Shin, Nam	150907	MK211033
S217	Coleophoridae	*Coleophora therinella*	Hwadong-salt Farm-Opposite, Nampo-ri, Beakryeong-myeon, Ongjin-gun, Incheon-si, Gyeonggi-do, Korea	37.9269189	(37° 55' 36.91" N)	124.69937	Park, Shin, Nam	150907	MK211032
S385	Coleophoridae	*Coleophora therinella*	Hwadong-salt Farm-Opposite, Nampo-ri, Beakryeong-myeon, Ongjin-gun, Incheon-si, Gyeonggi-do, Korea	37.9269189	(37° 55' 36.91" N)	124.69937	Park, Shin, Nam	150907	MK211030
Y220	Coleophoridae	*Coleophora therinella*	Hwadong-salt Farm-Opposite, Nampo-ri, Beakryeong-myeon, Ongjin-gun, Incheon-si, Gyeonggi-do, Korea	37.9269189	(37° 55' 36.91" N)	124.69937	Park, Shin, Nam	150907	MK211028
Y222	Coleophoridae	*Coleophora therinella*	Hwadong-salt Farm-Opposite, Nampo-ri, Beakryeong-myeon, Ongjin-gun, Incheon-si, Gyeonggi-do, Korea	37.9269189	(37° 55' 36.91" N)	124.69937	Park, Shin, Nam	150907	MK211027
S219	Coleophoridae	*Coleophora therinella*	Trail g, Jincheon-ri, Beakryeong-myeon, Ongjin-gun, Incheon-si, Gyeonggi-do, Korea	37.9522354	(37° 57' 8.05" N)	124.718596	Park, Shin, Nam	150908	MK211031
S423	Coleophoridae	*Coleophora trientella*	Duwoong-wetland, Sindu-ri, Wonbuk-myeon, Taean-gun, Chungcheongnam-do, Korea	36.835705	(36° 50' 8.54" N)	126.1956258	Park, Shin, Nam, Kim	150824	MK211026
S424	Coleophoridae	*Coleophora trientella*	Duwoong-wetland, Sindu-ri, Wonbuk-myeon, Taean-gun, Chungcheongnam-do, Korea	36.835705	(36° 50' 8.54" N)	126.1956258	Park, Shin, Nam, Kim	150824	MK211025
S425	Coleophoridae	*Coleophora trientella*	Duwoong-wetland, Sindu-ri, Wonbuk-myeon, Taean-gun, Chungcheongnam-do, Korea	36.835705	(36° 50' 8.54" N)	126.1956258	Park, Shin, Nam, Kim	150824	MK211024
S426	Coleophoridae	*Coleophora trientella*	Duwoong-wetland, Sindu-ri, Wonbuk-myeon, Taean-gun, Chungcheongnam-do, Korea	36.835705	(36° 50' 8.54" N)	126.1956258	Park, Shin, Nam, Kim	150824	MK211023
S389	Coleophoridae	*Coleophora versurella*	Mt. Danseok, Hwacheon-ri, Geoncheon-eup, Gyeongju-si, Gyeongsangbuk-do, Korea	35.788845	(35° 47' 19.84" N)	129.114107	Park, Shin, Kim, Nam	160615	MK211022
S395	Coleophoridae	*Coleophora versurella*	Yonggang-dong, Gyeongju-si, Gyeongsangbuk-do, Korea	35.8697361	(35° 52' 11.05" N)	129.2367455	Park, Shin, Nam, Kim	160613	MK211021
Y221	Coleophoridae	Coleophoridae sp1	Hwadong-salt Farm-Opposite, Nampo-ri, Beakryeong-myeon, Ongjin-gun, Incheon-si, Gyeonggi-do, Korea	37.9269189	(37° 55' 36.91" N)	124.69937	Park, Shin, Nam	150907	MK211020
S315	Gelechiidae	*Concubina* sp1	Korea National Arboretum, Jikdong-ri, Soheul-eup, Pocheon-si, Gyeonggi-do, Korea	37.7568443	(37° 45' 24.64" N)	127.1678772	S.J.Roh, Y.M.Shin	170720	MK211019
S317	Cosmopterigidae	Cosmopterigidae sp1	Beopheung-ri, Suju-myeon, Yeongwol-gun, Gangwon-do, Korea	37.3659601	(37° 21' 57.46" N)	128.2737367	Shon *et al*.	170727	MK211017
S344	Cosmopterigidae	Cosmopterigidae sp1	Beopheung-ri, Suju-myeon, Yeongwol-gun, Gangwon-do, Korea	37.3659601	(37° 21' 57.46" N)	128.2737367	Shon *et al*.	170727	MK211016
S182	Cosmopterigidae	Cosmopterigidae sp1	Jungsan-ri, Daedeok-myeon, Gimcheon-si, Gyeongsangbuk-do, Korea	35.9358889	(35° 56' 9.20" N)	127.9907773	S.R.Kim	120723	MK211018
S391	Cosmopterigidae	Cosmopterigidae sp2	Mt. Toham, Hwangyong-dong, Gyeongju-si, Gyeongsangbuk-do, Korea	35.8232501	(35° 49' 23.70" N)	129.3685615	Park, Shin, Nam, Kim	160901	MK211013
S218	Cosmopterigidae	Cosmopterigidae sp2	Trail B, Jincheon-ri, Beakryeong-myeon, Ongjin-gun, Incheon-si, Gyeonggi-do, Korea	37.9749722	(37° 58' 29.90" N)	124.7043481	Park, Shin, Nam	150909	MK211014
Y189	Cosmopterigidae	Cosmopterigidae sp2	Trail B, Jincheon-ri, Beakryeong-myeon, Ongjin-gun, Incheon-si, Gyeonggi-do, Korea	37.9749722	(37° 58' 29.90" N)	124.7043481	Park, Shin, Nam	150909	MK211012
Y191	Cosmopterigidae	Cosmopterigidae sp2	Trail B, Jincheon-ri, Beakryeong-myeon, Ongjin-gun, Incheon-si, Gyeonggi-do, Korea	37.9749722	(37° 58' 29.90" N)	124.7043481	Park, Shin, Nam	150909	MK211011
Y190	Cosmopterigidae	Cosmopterigidae sp3	Trail B, Jincheon-ri, Beakryeong-myeon, Ongjin-gun, Incheon-si, Gyeonggi-do, Korea	37.9749722	(37° 58' 29.90" N)	124.7043481	Park, Shin, Nam	150909	MK211010
S351	Cosmopterigidae	Cosmopterigidae sp4	Beopheung-ri, Suju-myeon, Yeongwol-gun, Gangwon-do, Korea	37.3659601	(37° 21' 57.46" N)	128.2737367	Shon *et al*.	170727	MK211015
S342	Cosmopterigidae	*Cosmopterix crassicervicella*	Beopheung-ri, Suju-myeon, Yeongwol-gun, Gangwon-do, Korea	37.3659601	(37° 21' 57.46" N)	128.2737367	Shon *et al*.	170727	MK211009
S343	Cosmopterigidae	*Cosmopterix crassicervicella*	Beopheung-ri, Suju-myeon, Yeongwol-gun, Gangwon-do, Korea	37.3659601	(37° 21' 57.46" N)	128.2737367	Shon *et al*.	170727	MK211008
S318	Cosmopterigidae	*Cosmopterix scribaieolla japonica*	Yonggang-dong, Gyeongju-si, Gyeongsangbuk-do, Korea	35.8697361	(35° 52' 11.05" N)	129.2367455	Park, Shin, Nam, Kim	160613	MK211007
Y242	Cosmopterigidae	*Cosmopterix* sp1	Mureung-ri, Daejeong-eup, Seogwipo-si, Is. Jeju, Korea	33.278215	(33° 16' 41.57" N)	126.25147	Park, Kim, Lee	170521	MK211006

S419	Cosmopterigidae	*Cosmopterix* sp2	Sanggye-dong, Nowon-gu, Seoul-si, Korea	37.6780046	(37° 40' 40.82" N)	127.0783244	Kim, Shin, Kim, Kim	170608	MK211005
S227	Depressariidae	*Cryptolechia* sp1	National D.M.Z Arboretum, Mandae-ri, Haean-myeon, Yanggu-gun, Gangwon-do, Korea	38.2538655	(38° 15' 13.92" N)	128.1122858	S.M.Oh	130726	MK210996
Y168	Depressariidae	*Cryptolechia* sp1	Yongdae recreation forest, Yongdae-ri, Buk-myeon, Inje-gun, Gangwon-do, Korea	38.2360777	(38° 14' 9.88" N)	128.3449028	Lim, Lim, Oh, Go	130731	MK210995
Y169	Depressariidae	*Cryptolechia* sp1	Yongdae recreation forest, Yongdae-ri, Buk-myeon, Inje-gun, Gangwon-do, Korea	38.2360777	(38° 14' 9.88" N)	128.3449028	Lim, Lim, Oh, Go	130731	MK210994
J30	Depressariidae	*Cryptolechia* sp1	Yongdae recreation forest, Yongdae-ri, Buk-myeon, Inje-gun, Gangwon-do, Korea	38.2360777	(38° 14' 9.88" N)	128.3449028	Y.R. Lee	130731	MK211004
J32	Depressariidae	*Cryptolechia* sp1	Yongdae recreation forest, Yongdae-ri, Buk-myeon, Inje-gun, Gangwon-do, Korea	38.2360777	(38° 14' 9.88" N)	128.3449028	Y.R. Lee	130731	MK211003
J33	Depressariidae	*Cryptolechia* sp1	Yongdae recreation forest, Yongdae-ri, Buk-myeon, Inje-gun, Gangwon-do, Korea	38.2360777	(38° 14' 9.88" N)	128.3449028	Y.R. Lee	130731	MK211002
J34	Depressariidae	*Cryptolechia* sp1	Yongdae recreation forest, Yongdae-ri, Buk-myeon, Inje-gun, Gangwon-do, Korea	38.2360777	(38° 14' 9.88" N)	128.3449028	Y.R. Lee	130731	MK211001
J35	Depressariidae	*Cryptolechia* sp1	Yongdae recreation forest, Yongdae-ri, Buk-myeon, Inje-gun, Gangwon-do, Korea	38.2360777	(38° 14' 9.88" N)	128.3449028	Y.R. Lee	130731	MK211000
J36	Depressariidae	*Cryptolechia* sp1	Yongdae recreation forest, Yongdae-ri, Buk-myeon, Inje-gun, Gangwon-do, Korea	38.2360777	(38° 14' 9.88" N)	128.3449028	Y.R. Lee	130731	MK210999
J37	Depressariidae	*Cryptolechia* sp1	Yongdae recreation forest, Yongdae-ri, Buk-myeon, Inje-gun, Gangwon-do, Korea	38.2360777	(38° 14' 9.88" N)	128.3449028	Y.R. Lee	130731	MK210998
J38	Depressariidae	*Cryptolechia* sp1	Yongdae recreation forest, Yongdae-ri, Buk-myeon, Inje-gun, Gangwon-do, Korea	38.2360777	(38° 14' 9.88" N)	128.3449028	Y.R. Lee	130731	MK210997
S181	Depressariidae	*Cryptolechia* sp2	Jungsan-ri, Daedeok-myeon, Gimcheon-si, Gyeongsangbuk-do, Korea	35.9358889	(35° 56' 9.20" N)	127.9907773	S.R.Kim	120723	MK210993
S43	Depressariidae	*Cryptolechia* sp2	Mt. Sokri, Samga-ri, Sokriisan-myeon, Boeun-gun, Chungcheongbuk-do, Korea	36.4893203	(36° 29' 21.55" N)	127.8593163	S.R.Kim	110725	MK210992
J26	Gelechiidae	*Deltophora korbi*	Gulup-ri, Seo-myeon, Hongcheon-gun, Gangwon-do, Korea	37.6224027	(37° 37' 20.65" N)	127.6838362	Y.R. Lee	130702	MK210991
S193	Gelechiidae	*Dendrophilia heotaphronoma*	Mt. Gyeryongsan, Hakbong-ri, Banpo-myeon, Gongju-si, Chungcheongnam-do, Korea	36.353291	(36° 21' 11.85" N)	127.2559724	S.R.Kim	120724	MK210990
S194	Gelechiidae	*Dendrophilia heotaphronoma*	Mt. Gyeryongsan, Hakbong-ri, Banpo-myeon, Gongju-si, Chungcheongnam-do, Korea	36.353291	(36° 21' 11.85" N)	127.2559724	S.R.Kim	120724	MK210989
Y157	Depressariidae	*Depressaria irregularis*	Seokpo-ri, Seokpo-myeon, Bonghwa-gun, Gyeongsangbuk-do, Korea	37.0521487	(37° 3' 7.74" N)	129.0861315	Nam, Kim, Kim, Park	160704	MK210988
Y158	Depressariidae	*Depressaria irregularis*	Seokpo-ri, Seokpo-myeon, Bonghwa-gun, Gyeongsangbuk-do, Korea	37.0521487	(37° 3' 7.74" N)	129.0861315	Nam, Kim, Kim, Park	160704	MK210987
Y159	Depressariidae	*Depressaria irregularis*	Seokpo-ri, Seokpo-myeon, Bonghwa-gun, Gyeongsangbuk-do, Korea	37.0521487	(37° 3' 7.74" N)	129.0861315	Nam, Kim, Kim, Park	160704	MK210986
Y67	Oecophoridae	*Deuterogonia pudonia*	Chusan-ri, Okryong-myeon, Gwangyang-si, Jeollanam-do, Korea	35.0467525	(35° 2' 48.31" N)	127.589623	Y.R. Lee	160618	MK210982
Y66	Oecophoridae	*Deuterogonia pudonia*	Mt. Jungmi, Shinbok-ri, Okcheon-myeon, Yangpyeong-gun, Gyeonggi-do, Korea	37.5807967	(37° 34' 50.87" N)	127.4579292	S.R.Kim	110628	MK210983
Y173	Oecophoridae	*Deuterogonia pudonia*	Seungeon-ri, Anmyeong-eup, Taean-gun, Chungcheongnam-do, Korea	36.4999659	(36° 29' 59.88" N)	126.3621608	Park, Shin, Kim, Nam	150826	MK210984
S209	Oecophoridae	*Deuterogonia pudonia*	Yuklim lake, Jikdong-ri, Soheul-eup, Pocheon-si, Gyeonggi-do, Korea	37.748548	(37° 44' 54.77" N)	127.1651502	Park, Nan, Shin, Kim, Son	150528	MK210985
J28	Gelechiidae	*Dichomeris heriguronis*	Chusan-ri, Okryong-myeon, Gwangyang-si, Jeollanam-do, Korea	35.0467525	(35° 2' 48.31" N)	127.589623	Y.R. Lee	160618	MK210981
Y140	Gelechiidae	*Dichomeris heriguronis*	Korea National Arboretum, Jikdong-ri, Soheul-eup, Pocheon-si, Gyeonggi-do, Korea	37.7520771	(37° 45' 7.48" N)	127.158586	Lee, Park, Lim, Lim, Kim, (	130613	MK210979
J29	Gelechiidae	*Dichomeris heriguronis*	Mt. Taehwa, Sangrim-ri, Docheok-myeon, Gwangju-si, Gyeonggi-do, Korea	37.3057197	(37° 18' 20.59" N)	127.3028244	S.R.Kim	130615	MK210980
S357	Gelechiidae	*Dichomeris* sp1	Sanggye-dong, Nowon-gu, Seoul-si, Korea	37.6780046	(37° 40' 40.82" N)	127.0783244	Kim, Shin, Kim, Kim	170608	MK210977
S421	Gelechiidae	*Dichomeris* sp1	Sanggye-dong, Nowon-gu, Seoul-si, Korea	37.6780046	(37° 40' 40.82" N)	127.0783244	Kim, Shin, Kim, Kim	170608	MK210975
S422	Gelechiidae	*Dichomeris* sp1	Sanggye-dong, Nowon-gu, Seoul-si, Korea	37.6780046	(37° 40' 40.82" N)	127.0783244	Kim, Shin, Kim, Kim	170608	MK210974
Y209	Gelechiidae	*Dichomeris* sp1	Yongdae recreation forest, Yongdae-ri, Buk-myeon, Inje-gun, Gangwon-do, Korea	38.2360777	(38° 14' 9.88" N)	128.3449028	Y.R. Lee	130731	MK210973
S373	Gelechiidae	*Dichomeris* sp1	Yuklim lake, Jikdong-ri, Soheul-eup, Pocheon-si, Gyeonggi-do, Korea	37.748548	(37° 44' 54.77" N)	127.1651502	Lee, Roh, Shin, Shon, Choi	170706	MK210976
S329	Gelechiidae	*Dichomeris* sp2	Mt. Toham, Hwangyong-dong, Gyeongju-si, Gyeongsangbuk-do, Korea	35.8232501	(35° 49' 23.70" N)	129.3685615	Park, Shin, Nam, Kim	160901	MK210972
S328	Gelechiidae	*Dichomeris* sp3	Mt. Geumo, Yeulim-ri, Dolsan-eup, Yeosu-gun, Jeollanam-do, Korea	34.5921879	(34° 35' 31.88" N)	127.8015618	Lim, Choi, Lee, Roh	170612	MK210978
Y187	Gelechiidae	*Encolapta tegulifera*	Korea National Arboretum, Jikdong-ri, Soheul-eup, Pocheon-si, Gyeonggi-do, Korea	37.7520771	(37° 45' 7.48" N)	127.158586	Lee, Park, Lim, Lim, Kim, (	130613	MK210969
S199	Gelechiidae	*Encolapta tegulifera*	Mt. Palgong, Dongsan-ri, Bugye-myeon, Gunwi-gun, Gyeongsangbuk-do, Korea	35.9726393	(35° 58' 21.50" N)	128.6636718	Park, Shin, Kim, Nam	140701	MK210971
S256	Gelechiidae	*Encolapta tegulifera*	Mt. Taehwa, Sangrim-ri, Docheok-myeon, Gwangju-si, Gyeonggi-do, Korea	37.3057197	(37° 18' 20.59" N)	127.3028244	S.R.Kim	130615	MK210970
Y106	Depressariidae	*Eutorna leonidi*	Yonggang-dong, Gyeongju-si, Gyeongsangbuk-do, Korea	35.8726052	(35° 52' 21.38" N)	129.2343208	Park, Shin, Nam, Kim	160730	MK210968
Y107	Depressariidae	*Eutorna leonidi*	Yonggang-dong, Gyeongju-si, Gyeongsangbuk-do, Korea	35.8726052	(35° 52' 21.38" N)	129.2343208	Park, Shin, Nam, Kim	160730	MK210967
Y82	Gelechiidae	*Evippe albidoesella*	Chusan-ri, Okryong-myeon, Gwangyang-si, Jeollanam-do, Korea	35.0491591	(35° 2' 56.97" N)	127.59797	Y.R. Lee	130726	MK210964
Y83	Gelechiidae	*Evippe albidoesella*	Chusan-ri, Okryong-myeon, Gwangyang-si, Jeollanam-do, Korea	35.0491591	(35° 2' 56.97" N)	127.59797	Y.R. Lee	130726	MK210963
Y93	Gelechiidae	*Evippe albidoesella*	Chusan-ri, Okryong-myeon, Gwangyang-si, Jeollanam-do, Korea	35.0491591	(35° 2' 56.97" N)	127.59797	Y.R. Lee	130726	MK210962
Y102	Gelechiidae	*Evippe albidoesella*	Chusan-ri, Okryong-myeon, Gwangyang-si, Jeollanam-do, Korea	35.0467525	(35° 2' 48.31" N)	127.589623	Y.R. Lee	160618	MK210966
Y134	Gelechiidae	*Evippe albidoesella*	Osan-ri, Gosan-myeon, Wanju-gun, Jeollabuk-do, Korea	35.9586532	(35° 57' 31.15" N)	127.2368809	S.R. Kim	120607	MK210965
S179	Gelechiidae	*Faristenia jumbongae*	Jungsan-ri, Daedeok-myeon, Gimcheon-si, Gyeongsangbuk-do, Korea	35.9358889	(35° 56' 9.20" N)	127.9907773	S.R.Kim	120723	MK210961
S186	Gelechiidae	*Faristenia jumbongae*	Jungsan-ri, Daedeok-myeon, Gimcheon-si, Gyeongsangbuk-do, Korea	35.9358889	(35° 56' 9.20" N)	127.9907773	S.R.Kim	120723	MK210960
S200	Gelechiidae	*Faristenia jumbongae*	Jungsan-ri, Daedeok-myeon, Gimcheon-si, Gyeongsangbuk-do, Korea	35.9358889	(35° 56' 9.20" N)	127.9907773	S.R.Kim	120723	MK210959
Y235	Gelechiidae	*Faristenia jumbongae*	Korea National Arboretum, Jikdong-ri, Soheul-eup, Pocheon-si, Gyeonggi-do, Korea	37.7520771	(37° 45' 7.48" N)	127.158586	Lee, Park, Lim, Lim, Kim, (	130613	MK210956
S294	Gelechiidae	*Faristenia jumbongae*	Mt. Taehwa, Sangrim-ri, Docheok-myeon, Gwangju-si, Gyeonggi-do, Korea	37.3057197	(37° 18' 20.59" N)	127.3028244	S.R.Kim	130615	MK210958
S295	Gelechiidae	*Faristenia jumbongae*	Mt. Taehwa, Sangrim-ri, Docheok-myeon, Gwangju-si, Gyeonggi-do, Korea	37.3057197	(37° 18' 20.59" N)	127.3028244	S.R.Kim	130615	MK210957
J5	Gelechiidae	*Faristenia nemoriella*	Mt. Taehwa, Sangrim-ri, Docheok-myeon, Gwangju-si, Gyeonggi-do, Korea	37.3057197	(37° 18' 20.59" N)	127.3028244	S.R.Kim	130615	MK210953
J44	Gelechiidae	*Faristenia nemoriella*	Yuklim lake, Jikdong-ri, Soheul-eup, Pocheon-si, Gyeonggi-do, Korea	37.748548	(37° 44' 54.77" N)	127.1651502	Park, Nan, Shin, Kim, Son	150528	MK210955
J45	Gelechiidae	*Faristenia nemoriella*	Yuklim lake, Jikdong-ri, Soheul-eup, Pocheon-si, Gyeonggi-do, Korea	37.748548	(37° 44' 54.77" N)	127.1651502	Park, Nan, Shin, Kim, Son	150528	MK210954
S310	Gelechiidae	*Faristenia* sp1	Korea National Arboretum, Jikdong-ri, Soheul-eup, Pocheon-si, Gyeonggi-do, Korea	37.7568443	(37° 45' 24.64" N)	127.1678772	S.J.Roh, Y.M.Shin	170807	MK210952
S379	Gelechiidae	*Faristenia* sp1	Korea National Arboretum, Jikdong-ri, Soheul-eup, Pocheon-si, Gyeonggi-do, Korea	37.7568443	(37° 45' 24.64" N)	127.1678772	S.J.Roh, Y.M.Shin	170807	MK210951
S380	Gelechiidae	*Faristenia* sp1	Sanggye-dong, Nowon-gu, Seoul-si, Korea	37.6780046	(37° 40' 40.82" N)	127.0783244	Kim, Lee, Shin, Roh, Kim	170611	MK210950
Y165	Gelechiidae	Gelechiidae sp1	Korea National Arboretum, Jikdong-ri, Soheul-eup, Pocheon-si, Gyeonggi-do, Korea	37.7520771	(37° 45' 7.48" N)	127.158586	Lee, Park, Lim, Lim, Kim, (	130613	MK210944
Y180	Gelechiidae	Gelechiidae sp2	Korea National Arboretum, Jikdong-ri, Soheul-eup, Pocheon-si, Gyeonggi-do, Korea	37.7520771	(37° 45' 7.48" N)	127.158586	Lee, Park, Lim, Kim, Oh	130613	MK210943
S299	Gelechiidae	Gelechiidae sp3	Chusan-ri, Okryong-myeon, Gwangyang-si, Jeollanam-do, Korea	35.0467525	(35° 2' 48.31" N)	127.589623	Y.R. Lee	160618	MK210942
S301	Gelechiidae	Gelechiidae sp3	Gulup-ri, Seo-myeon, Hongcheon-gun, Gangwon-do, Korea	37.6224027	(37° 37' 20.65" N)	127.6838362	Y.R. Lee	130702	MK210941
Y181	Gelechiidae	Gelechiidae sp3	Korea National Arboretum, Jikdong-ri, Soheul-eup, Pocheon-si, Gyeonggi-do, Korea	37.7520771	(37° 45' 7.48" N)	127.158586	Lee, Park, Lim, Kim, Oh	130613	MK210940
Y182	Gelechiidae	Gelechiidae sp3	Korea National Arboretum, Jikdong-ri, Soheul-eup, Pocheon-si, Gyeonggi-do, Korea	37.7520771	(37° 45' 7.48" N)	127.158586	Lee, Park, Lim, Kim, Oh	130613	MK210939
Y183	Gelechiidae	Gelechiidae sp4	Korea National Arboretum, Jikdong-ri, Soheul-eup, Pocheon-si, Gyeonggi-do, Korea	37.7520771	(37° 45' 7.48" N)	127.158586	Lee, Park, Lim, Kim, Oh	130613	MK210938
Y236	Gelechiidae	Gelechiidae sp5	Seokpo-ri, Seokpo-myeon, Bonghwa-gun, Gyeongsangbuk-do, Korea	37.0521487	(37° 3' 7.74" N)	129.0861315	Nam, Kim, Kim, Park	160704	MK210937
S378	Gelechiidae	Gelechiidae sp6	Sanggye-dong, Nowon-gu, Seoul-si, Korea	37.6780046	(37° 40' 40.82" N)	127.0783244	Kim, Shin, Kim, Kim	170608	MK210947
S382	Gelechiidae	Gelechiidae sp7	Sanggye-dong, Nowon-gu, Seoul-si, Korea	37.6780046	(37° 40' 40.82" N)	127.0783244	Kim, Lee, Shin, Roh, Kim	170611	MK210946
S415	Gelechiidae	Gelechiidae sp8	Sanggye-dong, Nowon-gu, Seoul-si, Korea	37.6780046	(37° 40' 40.82" N)	127.0783244	Kim, Lee, Shin, Roh, Kim	170611	MK210945
J1	Lecithoceridae	*Halolaguna sublaxata*	Yongdae recreation forest, Yongdae-ri, Buk-myeon, Inje-gun, Gangwon-do, Korea	38.2360777	(38° 14' 9.88" N)	128.3449028	Y.R. Lee	130631	MK210936
J2	Lecithoceridae	*Halolaguna sublaxata*	Yongdae recreation forest, Yongdae-ri, Buk-myeon, Inje-gun, Gangwon-do, Korea	38.2360777	(38° 14' 9.88" N)	128.3449028	Y.R. Lee	130631	MK210935
S222	Gelechiidae	*Helcystogramma macroscopa*	Trail g, Jincheon-ri, Beakryeong-myeon, Ongjin-gun, Incheon-si, Gyeonggi-do, Korea	37.9522354	(37° 57' 8.05" N)	124.718596	Park, Shin, Kim	150513	MK210934
S308	Gelechiidae	*Helcystogramma perelegans*	Korea National Arboretum, Jikdong-ri, Soheul-eup, Pocheon-si, Gyeonggi-do, Korea	37.7568443	(37° 45' 24.64" N)	127.1678772	S.J.Roh, Y.M.Shin	170807	MK210933

S347	Gelechiidae	*Helcystogramma* sp1	Mamyeong-ri, Naechon-myeon, Pocheon-si, Gyeonggi-do, Korea	37.7832695	(37° 46' 59.77" N)	127.195408	E.W.Lee	130727	MK210931
S309	Gelechiidae	*Helcystogramma* sp2	Korea National Arboretum, Jikdong-ri, Soheul-eup, Pocheon-si, Gyeonggi-do, Korea	37.7568443	(37° 45' 24.64" N)	127.1678772	S.J.Roh, Y.M.Shin	170807	MK210932
S137	Stathmopodidae	*Hieromantis kurokoi*	Gulup-ri, Seo-myeon, Hongcheon-gun, Gangwon-do, Korea	37.6224027	(37° 37' 20.65" N)	127.6838362	Y.R. Lee	130702	MK210929
S409	Stathmopodidae	*Hieromantis kurokoi*	Hwadong-salt Farm-Opposite, Nampo-ri, Beakryeong-myeon, Ongjin-gun, Incheon-si, Gyeonggi-do, Korea	37.9269189	(37° 55' 36.91" N)	124.69937	Park, Shin, Nam	150907	MK210928
S136	Stathmopodidae	*Hieromantis kurokoi*	Naehyeon-ri, Seo-myeon, Yangyang-gun, Gangwon-do, Korea	38.0019129	(38° 0' 6.89" N)	128.6038909	Y.R. Lee	130813	MK210930
Y192	Stathmopodidae	*Hieromantis kurokoi*	Trail B, Jincheon-ri, Beakryeong-myeon, Ongjin-gun, Incheon-si, Gyeonggi-do, Korea	37.9749722	(37° 58' 29.90" N)	124.7043481	Park, Shin, Nam	150909	MK210927
S189	Gelechiidae	*Hypatima excellentella*	Mt. Gyeryongsan, Hakbong-ri, Banpo-myeon, Gongju-si, Chungcheongnam-do, Korea	36.353291	(36° 21' 11.85" N)	127.2559724	S.R.Kim	120724	MK210925
J6	Gelechiidae	*Hypatima excellentella*	Yongdae recreation forest, Yongdae-ri, Buk-myeon, Inje-gun, Gangwon-do, Korea	38.2360777	(38° 14' 9.88" N)	128.3449028	Y.R. Lee	130731	MK210926
Y114	Cosmopterigidae	*Labdia antennella*	Chusan-ri, Okryong-myeon, Gwangyang-si, Jeollanam-do, Korea	35.0491591	(35° 2' 56.97" N)	127.59797	Y.R. Lee	130726	MK210924
Y118	Cosmopterigidae	*Labdia antennella*	Chusan-ri, Okryong-myeon, Gwangyang-si, Jeollanam-do, Korea	35.0491591	(35° 2' 56.97" N)	127.59797	Y.R. Lee	130726	MK210923
Y119	Cosmopterigidae	*Labdia antennella*	Chusan-ri, Okryong-myeon, Gwangyang-si, Jeollanam-do, Korea	35.0491591	(35° 2' 56.97" N)	127.59797	Y.R. Lee	130726	MK210922
Y120	Cosmopterigidae	*Labdia antennella*	Chusan-ri, Okryong-myeon, Gwangyang-si, Jeollanam-do, Korea	35.0491591	(35° 2' 56.97" N)	127.59797	Y.R. Lee	130726	MK210921
Y121	Cosmopterigidae	*Labdia antennella*	Chusan-ri, Okryong-myeon, Gwangyang-si, Jeollanam-do, Korea	35.0491591	(35° 2' 56.97" N)	127.59797	Y.R. Lee	130726	MK210920
Y122	Cosmopterigidae	*Labdia antennella*	Chusan-ri, Okryong-myeon, Gwangyang-si, Jeollanam-do, Korea	35.0491591	(35° 2' 56.97" N)	127.59797	Y.R. Lee	130726	MK210919
Y123	Cosmopterigidae	*Labdia antennella*	Chusan-ri, Okryong-myeon, Gwangyang-si, Jeollanam-do, Korea	35.0491591	(35° 2' 56.97" N)	127.59797	Y.R. Lee	130726	MK210918
Y124	Cosmopterigidae	*Labdia antennella*	Chusan-ri, Okryong-myeon, Gwangyang-si, Jeollanam-do, Korea	35.0491591	(35° 2' 56.97" N)	127.59797	Y.R. Lee	130726	MK210917
Y125	Cosmopterigidae	*Labdia antennella*	Chusan-ri, Okryong-myeon, Gwangyang-si, Jeollanam-do, Korea	35.0491591	(35° 2' 56.97" N)	127.59797	Y.R. Lee	130726	MK210916
Y115	Cosmopterigidae	*Labdia niphosticta*	Chusan-ri, Okryong-myeon, Gwangyang-si, Jeollanam-do, Korea	35.0491591	(35° 2' 56.97" N)	127.59797	Y.R. Lee	130726	MK210911
Y117	Cosmopterigidae	*Labdia niphosticta*	Chusan-ri, Okryong-myeon, Gwangyang-si, Jeollanam-do, Korea	35.0491591	(35° 2' 56.97" N)	127.59797	Y.R. Lee	130726	MK210910
J55	Cosmopterigidae	*Labdia niphosticta*	Jungsan-ri, Daedeok-myeon, Gimcheon-si, Gyeongsangbuk-do, Korea	35.9358889	(35° 56' 9.20" N)	127.9907773	S.R.Kim	120723	MK210915
S348	Cosmopterigidae	*Labdia niphosticta*	Korea National Arboretum, Jikdong-ri, Soheul-eup, Pocheon-si, Gyeonggi-do, Korea	37.7568443	(37° 45' 24.64" N)	127.1678772	Shon *et al*.	170727	MK210914
S349	Cosmopterigidae	*Labdia niphosticta*	Korea National Arboretum, Jikdong-ri, Soheul-eup, Pocheon-si, Gyeonggi-do, Korea	37.7568443	(37° 45' 24.64" N)	127.1678772	Lim *et al*.	170731	MK210913
S350	Cosmopterigidae	*Labdia niphosticta*	Korea National Arboretum, Jikdong-ri, Soheul-eup, Pocheon-si, Gyeonggi-do, Korea	37.7568443	(37° 45' 24.64" N)	127.1678772	Lim *et al*.	170731	MK210912
Y240	Cosmopterigidae	*Labdia semicoccinea*	Beopheung-ri, Suju-myeon, Yeongwol-gun, Gangwon-do, Korea	37.3659601	(37° 21' 57.46" N)	128.2737367	S.J.Roh, Y.M.Shin	170727	MK210908
S319	Cosmopterigidae	*Labdia semicoccinea*	Beopheung-ri, Suju-myeon, Yeongwol-gun, Gangwon-do, Korea	37.3659601	(37° 21' 57.46" N)	128.2737367	S.J.Roh, Y.M.Shin	170727	MK210909
S555	Cosmopterigidae	*Labdia* sp1	Chusan-ri, Okryong-myeon, Gwangyang-si, Jeollanam-do, Korea	35.0491591	(35° 2' 56.97" N)	127.59797	Y.R. Lee	130726	MK210907
Y141	Lecithoceridae	*Lecithocera thiodora*	Gonam-ri, Gonam-myeon, Taean-gun, Chungcheongnam-do, Korea	36.4225034	(36° 25' 21.01" N)	126.4033907	Park, Shin, Kim	150609	MK210906
Y167	Lecithoceridae	*Lecithocera thiodora*	Seungeon-ri, Anmyeong-eup, Taean-gun, Chungcheongnam-do, Korea	36.4999659	(36° 29' 59.88" N)	126.3621608	Park, Shin, Kim, Nam	150826	MK210905
S239	Lecithoceridae	Lecithoceridae sp1	Mt. Daeso, Songhyeon-ri, Sowon-myeon, Taean-gun, Chungcheongnam-do, Korea	36.7731411	(36° 46' 23.31" N)	126.1794459	Park, Shin, Kim	150610	MK210904
S372	Lecithoceridae	Lecithoceridae sp1	Mt. Daeso, Songhyeon-ri, Sowon-myeon, Taean-gun, Chungcheongnam-do, Korea	36.7731411	(36° 46' 23.31" N)	126.1794459	Park, Shin, Kim	150610	MK210903
S400	Lecithoceridae	Lecithoceridae sp1	Mt. Daeso, Songhyeon-ri, Sowon-myeon, Taean-gun, Chungcheongnam-do, Korea	36.7731411	(36° 46' 23.31" N)	126.1794459	Park, Shin, Kim	150610	MK210902
S399	Lecithoceridae	Lecithoceridae sp2	Gonam-ri, Gonam-myeon, Taean-gun, Chungcheongnam-do, Korea	36.4225034	(36° 25' 21.01" N)	126.4033907	Park, Shin, Kim	150609	MK210899
S221	Lecithoceridae	Lecithoceridae sp2	Mt. Daeso, Songhyeon-ri, Sowon-myeon, Taean-gun, Chungcheongnam-do, Korea	36.7731411	(36° 46' 23.31" N)	126.1794459	Park, Shin, Kim	150610	MK210901
S401	Lecithoceridae	Lecithoceridae sp2	Mt. Daeso, Songhyeon-ri, Sowon-myeon, Taean-gun, Chungcheongnam-do, Korea	36.7731411	(36° 46' 23.31" N)	126.1794459	Park, Shin, Kim	150610	MK210898
S402	Lecithoceridae	Lecithoceridae sp2	Mt. Daeso, Songhyeon-ri, Sowon-myeon, Taean-gun, Chungcheongnam-do, Korea	36.7731411	(36° 46' 23.31" N)	126.1794459	Park, Shin, Kim	150610	MK210897
S403	Lecithoceridae	Lecithoceridae sp2	Mt. Daeso, Songhyeon-ri, Sowon-myeon, Taean-gun, Chungcheongnam-do, Korea	36.7731411	(36° 46' 23.31" N)	126.1794459	Park, Shin, Kim	150610	MK210896
S374	Lecithoceridae	Lecithoceridae sp2	Mt. Geumo, Yeulim-ri, Dolsan-eup, Yeosu-gun, Jeollanam-do, Korea	34.5921879	(34° 35' 31.88" N)	127.8015618	Lim, Choi, Lee, Roh	170612	MK210900
S225	Lecithoceridae	Lecithoceridae sp3	Trail g, Jincheon-ri, Beakryeong-myeon, Ongjin-gun, Incheon-si, Gyeonggi-do, Korea	37.9522354	(37° 57' 8.05" N)	124.718596	Park, Shin, Kim	150707	MK210895
Y193	Depressariidae	*Letogenes festalis*	National D.M.Z Arboretum, Mandae-ri, Haean-myeon, Yanggu-gun, Gangwon-do, Korea	38.2538655	(38° 15' 13.92" N)	128.1122858	S.M.Oh	130726	MK210893
S23	Depressariidae	*Letogenes festalis*	Wonpung-ri, Yeonpung-myeon, Goesan-gun, Chungcheongbuk-do, Korea	36.8003718	(36° 48' 1.34" N)	128.0487888	S.R.Kim	110630	MK210894
J3	Cosmopterigidae	*Limaeciaa phragmitella*	Chusan-ri, Okryong-myeon, Gwangyang-si, Jeollanam-do, Korea	35.0467525	(35° 2' 48.31" N)	127.589623	Y.R. Lee	160618	MK210892
S206	Lecithoceridae	*Martyringa ussuriella*	Seokpo-ri, Seokpo-myeon, Bonghwa-gun, Gyeongsangbuk-do, Korea	37.0521487	(37° 3' 7.74" N)	129.0861315	Nam, Kim, Kim, Park	160704	MK210891
S207	Lecithoceridae	*Martyringa ussuriella*	Seokpo-ri, Seokpo-myeon, Bonghwa-gun, Gyeongsangbuk-do, Korea	37.0521487	(37° 3' 7.74" N)	129.0861315	Nam, Kim, Kim, Park	160704	MK210890
Y34	Xyloryctidae	*Meleonoma fustiformis*	Gamsan-ri, Sannae-myeon, Gyeongju-si, Gyeongsangbuk-do, Korea	35.8022478	(35° 48' 8.09" N)	129.0448524	Park, Shin, Kim	140821	MK210882
Y26	Xyloryctidae	*Meleonoma fustiformis*	Harye-ri, Namwon-eup, Seogwipo-si, Is. Jeju, Korea	33.3252568	(33° 19' 30.92" N)	126.6020681	Park, Kim, Lee	140619	MK210885
Y28	Xyloryctidae	*Meleonoma fustiformis*	Harye-ri, Namwon-eup, Seogwipo-si, Is. Jeju, Korea	33.3252568	(33° 19' 30.92" N)	126.6020681	Park, Kim, Lee	140619	MK210883
Y170	Xyloryctidae	*Meleonoma fustiformis*	Janggok-ri, Gonam-myeon, Taean-gun, Chungcheongnam-do, Korea	36.4424137	(36° 26' 32.69" N)	126.3875925	Park, Shin, Kim	150826	MK210888
Y27	Xyloryctidae	*Meleonoma fustiformis*	Sanghyo-dong, Seogwipo-si, Is. Jeju, Korea	33.3012848	(33° 18' 4.63" N)	126.5816049	Park, Kim, Lee	140619	MK210884
J31	Xyloryctidae	*Meleonoma fustiformis*	Sinrye-ri, Namwon-eup, Seogwipo-si, Is. Jeju, Korea	33.3180711	(33° 19' 5.06" N)	126.6294507	Park, Kim, Lee	140619	MK210889
Y24	Xyloryctidae	*Meleonoma fustiformis*	Sinrye-ri, Namwon-eup, Seogwipo-si, Is. Jeju, Korea	33.3180711	(33° 19' 5.06" N)	126.6294507	Park, Kim, Lee	140619	MK210887
Y25	Xyloryctidae	*Meleonoma fustiformis*	Sinrye-ri, Namwon-eup, Seogwipo-si, Is. Jeju, Korea	33.3180711	(33° 19' 5.06" N)	126.6294507	Park, Kim, Lee	140619	MK210886
J18	Xyloryctidae	*Meleonoma malacobyrsa*	Chusan-ri, Okryong-myeon, Gwangyang-si, Jeollanam-do, Korea	35.0491591	(35° 2' 56.97" N)	127.59797	Y.R. Lee	130726	MK210809
J16	Xyloryctidae	*Meleonoma malacobyrsa*	Jungsan-ri, Daedeok-myeon, Gimcheon-si, Gyeongsangbuk-do, Korea	35.9358889	(35° 56' 9.20" N)	127.9907773	S.R.Kim	120723	MK210810
S154	Xyloryctidae	*Meleonoma malacobyrsa*	National D.M.Z Arboretum, Mandae-ri, Haean-myeon, Yanggu-gun, Gangwon-do, Korea	38.2538655	(38° 15' 13.92" N)	128.1122858	S.M.Oh	130726	MK210805
S155	Xyloryctidae	*Meleonoma malacobyrsa*	National D.M.Z Arboretum, Mandae-ri, Haean-myeon, Yanggu-gun, Gangwon-do, Korea	38.2538655	(38° 15' 13.92" N)	128.1122858	S.M.Oh	130726	MK210804
S156	Xyloryctidae	*Meleonoma malacobyrsa*	National D.M.Z Arboretum, Mandae-ri, Haean-myeon, Yanggu-gun, Gangwon-do, Korea	38.2538655	(38° 15' 13.92" N)	128.1122858	S.M.Oh	130726	MK210803
S359	Xyloryctidae	*Meleonoma torophanes*	Korea National Arboretum, Jikdong-ri, Soheul-eup, Pocheon-si, Gyeonggi-do, Korea	37.7568443	(37° 45' 24.64" N)	127.1678772	S.J.Roh, Y.M.Shin	170627	MK210881
Y104	Xyloryctidae	*Meleonoma torophanes*	Mt. Jeombong, Jindong-ri, Girin-myeon, Inje-gun, Gangwon-do, Korea	38.0027202	(38° 0' 9.79" N)	128.4817196	S.W. Park	130628	MK210880
Y105	Xyloryctidae	*Meleonoma torophanes*	Seokpo-ri, Seokpo-myeon, Bonghwa-gun, Gyeongsangbuk-do, Korea	37.0521487	(37° 3' 7.74" N)	129.0861315	Nam, Kim, Kim, Park	160704	MK210879
S128	Gelechiidae	*Mesophleps albilinella*	Chusan-ri, Okryong-myeon, Gwangyang-si, Jeollanam-do, Korea	35.0491591	(35° 2' 56.97" N)	127.59797	Y.R. Lee	130726	MK210877
S129	Gelechiidae	*Mesophleps albilinella*	Chusan-ri, Okryong-myeon, Gwangyang-si, Jeollanam-do, Korea	35.0491591	(35° 2' 56.97" N)	127.59797	Y.R. Lee	130726	MK210876
S130	Gelechiidae	*Mesophleps albilinella*	Chusan-ri, Okryong-myeon, Gwangyang-si, Jeollanam-do, Korea	35.0491591	(35° 2' 56.97" N)	127.59797	Y.R. Lee	130726	MK210875
S132	Gelechiidae	*Mesophleps albilinella*	Jungsan-ri, Daedeok-myeon, Gimcheon-si, Gyeongsangbuk-do, Korea	35.9358889	(35° 56' 9.20" N)	127.9907773	S.R.Kim	120723	MK210873
S131	Gelechiidae	*Mesophleps albilinella*	Mt. Gyeryongsan, Hakbong-ri, Banpo-myeon, Gongju-si, Chungcheongnam-do, Korea	36.3573002	(36° 21' 26.28" N)	127.2423253	S.R.Kim	120724	MK210874
S127	Gelechiidae	*Mesophleps albilinella*	Yongdae recreation forest, Yongdae-ri, Buk-myeon, Inje-gun, Gangwon-do, Korea	38.2360777	(38° 14' 9.88" N)	128.3449028	Y.R. Lee	130731	MK210878
S353	Gelechiidae	*Metzneria* sp1	Yonggang-dong, Gyeongju-si, Gyeongsangbuk-do, Korea	35.8697361	(35° 52' 11.05" N)	129.2367455	Park, Shin, Nam, Kim	160613	MK210872
J4	Gelechiidae	*Monochroa cytisella*	Chusan-ri, Okryong-myeon, Gwangyang-si, Jeollanam-do, Korea	35.0467525	(35° 2' 48.31" N)	127.589623	Y.R. Lee	160618	MK210871
J52	Gelechiidae	*Monochroa* sp1	Mt. Baekhwa, Sangok-ri, Taean-eup, Taean-gun, Chungcheongnam-do, Korea	36.7737059	(36° 46' 25.34" N)	126.3226203	Park, Shin, Kim	150611	MK210870
J56	Gelechiidae	*Monochroa* sp1	Osan-ri, Gosan-myeon, Wanju-gun, Jeollabuk-do, Korea	35.9586532	(35° 57' 31.15" N)	127.2368809	S.R. Kim	120607	MK210869
J57	Gelechiidae	*Monochroa* sp1	Osan-ri, Gosan-myeon, Wanju-gun, Jeollabuk-do, Korea	35.9586532	(35° 57' 31.15" N)	127.2368809	S.R. Kim	120607	MK210868
J58	Gelechiidae	*Monochroa* sp1	Osan-ri, Gosan-myeon, Wanju-gun, Jeollabuk-do, Korea	35.9586532	(35° 57' 31.15" N)	127.2368809	S.R. Kim	120607	MK210867

S183	Gelechiidae	*Monochroa* sp2	Jungsan-ri, Daedeok-myeon, Gimcheon-si, Gyeongsangbuk-do, Korea	35.9358889	(35° 56' 9.20" N)	127.9907773	S.R.Kim	120723	MK210866
S238	Blastobasidae	*Neoblastobasis biceratala*	Chusan-ri, Okryong-myeon, Gwangyang-si, Jeollanam-do, Korea	35.0491591	(35° 2' 56.97" N)	127.59797	Y.R. Lee	130726	MK210865
S33	Blastobasidae	*Neoblastobasis biceratala*	Hakbong-ri, Banpo-myeon, Gongju-si, Chungcheongnam-do, Korea	36.353291	(36° 21' 11.85" N)	127.2559724	S.R.Kim	120724	MK210860
S35	Blastobasidae	*Neoblastobasis biceratala*	Hakbong-ri, Banpo-myeon, Gongju-si, Chungcheongnam-do, Korea	36.353291	(36° 21' 11.85" N)	127.2559724	S.R.Kim	120724	MK210858
S27	Blastobasidae	*Neoblastobasis biceratala*	Mt. Jungmi, Shinbok-ri, Okcheon-myeon, Yangpyeong-gun, Gyeonggi-do, Korea	37.5807967	(37° 34' 50.87" N)	127.4579292	S.R.Kim	110628	MK210863
S31	Blastobasidae	*Neoblastobasis biceratala*	Mt. Jungmi, Shinbok-ri, Okcheon-myeon, Yangpyeong-gun, Gyeonggi-do, Korea	37.5807967	(37° 34' 50.87" N)	127.4579292	S.R.Kim	110901	MK210862
S32	Blastobasidae	*Neoblastobasis biceratala*	Mt. Jungmi, Shinbok-ri, Okcheon-myeon, Yangpyeong-gun, Gyeonggi-do, Korea	37.5807967	(37° 34' 50.87" N)	127.4579292	S.R.Kim	110901	MK210861
S37	Blastobasidae	*Neoblastobasis biceratala*	Mt. Jungmi, Shinbok-ri, Okcheon-myeon, Yangpyeong-gun, Gyeonggi-do, Korea	37.5807967	(37° 34' 50.87" N)	127.4579292	S.R.Kim	110901	MK210857
S333	Blastobasidae	*Neoblastobasis biceratala*	Mt. Nam, Bae-dong, Gyeongju-si, Gyeongsangbuk-do, Korea	35.8075323	(35° 48' 27.12" N)	129.2181751	Park, Shin, Kim, Nam	160614	MK210859
Y206	Blastobasidae	*Neoblastobasis biceratala*	National D.M.Z Arboretum, Mandae-ri, Haean-myeon, Yanggu-gun, Gangwon-do, Korea	38.2538655	(38° 15' 13.92" N)	128.1122858	S.M.Oh	130726	MK210856
Y212	Blastobasidae	*Neoblastobasis biceratala*	Yongdae recreation forest, Yongdae-ri, Buk-myeon, Inje-gun, Gangwon-do, Korea	38.2360777	(38° 14' 9.88" N)	128.3449028	Lim, Lim, Oh, Go	130731	MK210855
Y216	Blastobasidae	*Neoblastobasis biceratala*	Yongdae recreation forest, Yongdae-ri, Buk-myeon, Inje-gun, Gangwon-do, Korea	38.2360777	(38° 14' 9.88" N)	128.3449028	Lim, Lim, Oh, Go	130731	MK210854
Y217	Blastobasidae	*Neoblastobasis biceratala*	Yongdae recreation forest, Yongdae-ri, Buk-myeon, Inje-gun, Gangwon-do, Korea	38.2360777	(38° 14' 9.88" N)	128.3449028	Lim, Lim, Oh, Go	130731	MK210853
S267	Blastobasidae	*Neoblastobasis biceratala*	Yongdae recreation forest, Yongdae-ri, Buk-myeon, Inje-gun, Gangwon-do, Korea	38.2360777	(38° 14' 9.88" N)	128.3449028	Y.R. Lee	130731	MK210864
s134	Blastobasidae	*Neoblastobasis longicornutela*	Chusan-ri, Okryong-myeon, Gwangyang-si, Jeollanam-do, Korea	35.0467525	(35° 2' 48.31" N)	127.589623	Y.R. Lee	160618	MK210849
J53	Blastobasidae	*Neoblastobasis longicornutela*	Mt. Daeso, Songhyeon-ri, Sowon-myeon, Taean-gun, Chungcheongnam-do, Korea	36.7731411	(36° 46' 23.31" N)	126.1794459	Park, Shin, Kim	150610	MK210851
J54	Blastobasidae	*Neoblastobasis longicornutela*	Mt. Daeso, Songhyeon-ri, Sowon-myeon, Taean-gun, Chungcheongnam-do, Korea	36.7731411	(36° 46' 23.31" N)	126.1794459	Park, Shin, Kim	150610	MK210850
S335	Blastobasidae	*Neoblastobasis longicornutela*	Mt. Geumo, Yeulim-ri, Dolsan-eup, Yeosu-gun, Jeollanam-do, Korea	34.5921879	(34° 35' 31.88" N)	127.8015618	Lim, Choi, Lee, Roh	170612	MK210848
J46	Blastobasidae	*Neoblastobasis longicornutela*	Yuklim lake, Jikdong-ri, Soheul-eup, Pocheon-si, Gyeonggi-do, Korea	37.748548	(37° 44' 54.77" N)	127.1651502	Park, Nan, Shin, Kim, Son	150528	MK210852
S135	Blastobasidae	*Neoblastobasis spiniharpella*	Chusan-ri, Okryong-myeon, Gwangyang-si, Jeollanam-do, Korea	35.0467525	(35° 2' 48.31" N)	127.589623	Y.R. Lee	160618	MK210847
S143	Blastobasidae	*Neoblastobasis spiniharpella*	Chusan-ri, Okryong-myeon, Gwangyang-si, Jeollanam-do, Korea	35.0467525	(35° 2' 48.31" N)	127.589623	Y.R. Lee	160618	MK210846
S336	Blastobasidae	*Neoblastobasis spiniharpella*	Geumbong-ri, Dolsan-eup, Yeosu-gun, Jeollanam-do, Korea	34.6431937	(34° 38' 35.50" N)	127.7493644	Lim, Choi, Lee, Roh	170612	MK210844
S334	Blastobasidae	*Neoblastobasis spiniharpella*	Mt. Toham, Hwangyong-dong, Gyeongju-si, Gyeongsangbuk-do, Korea	35.8154726	(35° 48' 55.70" N)	129.3557298	Park, Shin, Nam, Kim	160614	MK210845
S30	Blastobasidae	*Neoblastobasis sprotundalis*	Mt. Sokri, Samga-ri, Sokriisan-myeon, Boeun-gun, Chungcheongbuk-do, Korea	36.4893203	(36° 29' 21.55" N)	127.8593163	S.R.Kim	110725	MK210843
Y203	Blastobasidae	*Neoblastobasis sprotundalis*	Yuklim lake, Jikdong-ri, Soheul-eup, Pocheon-si, Gyeonggi-do, Korea	37.748548	(37° 44' 54.77" N)	127.1651502	Park, Shin, Nam	150716	MK210842
Y91	Gelechiidae	*Nuntia* sp1	Chusan-ri, Okryong-myeon, Gwangyang-si, Jeollanam-do, Korea	35.0491591	(35° 2' 56.97" N)	127.59797	Y.R. Lee	130726	MK210840
Y90	Gelechiidae	*Nuntia* sp1	Yongdae recreation forest, Yongdae-ri, Buk-myeon, Inje-gun, Gangwon-do, Korea	38.2360777	(38° 14' 9.88" N)	128.3449028	Y.R. Lee	130731	MK210841
S313	Oecophoridae	Oecophoridae sp1	Korea National Arboretum, Jikdong-ri, Soheul-eup, Pocheon-si, Gyeonggi-do, Korea	37.7568443	(37° 45' 24.64" N)	127.1678772	S.J.Roh, Y.M.Shin	170720	MK210839
S314	Oecophoridae	Oecophoridae sp1	Korea National Arboretum, Jikdong-ri, Soheul-eup, Pocheon-si, Gyeonggi-do, Korea	37.7568443	(37° 45' 24.64" N)	127.1678772	S.J.Roh, Y.M.Shin	170720	MK210838
S360	Oecophoridae	Oecophoridae sp2	Beopheung-ri, Suju-myeon, Yeongwol-gun, Gangwon-do, Korea	37.3659601	(37° 21' 57.46" N)	128.2737367	Shon *et al*.	170727	MK210837
J8	Cosmopterigidae	*Pancalica* sp1	Daehyeon-dong, Seodaemun-gu, Seoul-si, Korea	37.5648686	(37° 33' 53.53" N)	126.9491035	Y.R. Lee	130513	MK210836
J9	Cosmopterigidae	*Pancalica* sp1	Sinlim-dong, Gwanak-gu, Seoul-si, Korea	37.4479135	(37° 26' 52.49" N)	126.9525906	Y.R. Lee	130512	MK210835
S187	Gelechiidae	*Parachronistis jiriensis*	Mt. Gyeryongsan, Hakbong-ri, Banpo-myeon, Gongju-si, Chungcheongnam-do, Korea	36.353291	(36° 21' 11.85" N)	127.2559724	S.R.Kim	120724	MK210834
S412	Gelechiidae	*Parachronistis sellaris*	Korea National Arboretum, Jikdong-ri, Soheul-eup, Pocheon-si, Gyeonggi-do, Korea	37.7506858	(37° 45' 2.47" N)	127.1678128	Lim, Roh, Lee, Choi, Shin	170530	MK210833
S417	Gelechiidae	*Parachronistis sellaris*	Mt. Gumi, Gajeong-ri, Hyeongok-myeon, Gyeongju-si, Gyeongsangbuk-do, Korea	35.9006093	(35° 54' 2.19" N)	129.1480005	Park, Shin, Kim, Nam	160616	MK210832
Y226	Gelechiidae	*Parachronistis* sp	Mt. Daeso, Songhyeon-ri, Sowon-myeon, Taean-gun, Chungcheongnam-do, Korea	36.7731411	(36° 46' 23.31" N)	126.1794459	Park, Shin, Kim	150610	MK210831
Y228	Gelechiidae	*Parachronistis* sp	Mt. Daeso, Songhyeon-ri, Sowon-myeon, Taean-gun, Chungcheongnam-do, Korea	36.7731411	(36° 46' 23.31" N)	126.1794459	Park, Shin, Kim	150610	MK210830
Y231	Gelechiidae	*Parachronistis* sp	Mt. Daeso, Songhyeon-ri, Sowon-myeon, Taean-gun, Chungcheongnam-do, Korea	36.7731411	(36° 46' 23.31" N)	126.1794459	Park, Shin, Kim	150610	MK210829
Y232	Gelechiidae	*Parachronistis* sp	Mt. Daeso, Songhyeon-ri, Sowon-myeon, Taean-gun, Chungcheongnam-do, Korea	36.7731411	(36° 46' 23.31" N)	126.1794459	Park, Shin, Kim	150610	MK210828
S289	Gelechiidae	*Parastenolechia suriensis*	Chusan-ri, Okryong-myeon, Gwangyang-si, Jeollanam-do, Korea	35.0491591	(35° 2' 56.97" N)	127.59797	Y.R. Lee	130726	MK210824
S296	Gelechiidae	*Parastenolechia suriensis*	Chusan-ri, Okryong-myeon, Gwangyang-si, Jeollanam-do, Korea	35.0491591	(35° 2' 56.97" N)	127.59797	Y.R. Lee	130726	MK210823
S151	Gelechiidae	*Parastenolechia suriensis*	Chusan-ri, Okryong-myeon, Gwangyang-si, Jeollanam-do, Korea	35.0491591	(35° 2' 56.97" N)	127.59797	Y.R. Lee	130726	MK210827
S252	Gelechiidae	*Parastenolechia suriensis*	Gulup-ri, Seo-myeon, Hongcheon-gun, Gangwon-do, Korea	37.6224027	(37° 37' 20.65" N)	127.6838362	Y.R. Lee	130702	MK210825
S190	Gelechiidae	*Parastenolechia suriensis*	Mt. Gyeryongsan, Hakbong-ri, Banpo-myeon, Gongju-si, Chungcheongnam-do, Korea	36.353291	(36° 21' 11.85" N)	127.2559724	S.R.Kim	120724	MK210826
S185	Gelechiidae	*Parastenolechia albicupitella*	Jungsan-ri, Daedeok-myeon, Gimcheon-si, Gyeongsangbuk-do, Korea	35.9358889	(35° 56' 9.20" N)	127.9907773	S.R.Kim	120723	MK210820
S157	Gelechiidae	*Parastenolechia albicupitella*	Yuklim lake, Jikdong-ri, Soheul-eup, Pocheon-si, Gyeonggi-do, Korea	37.748548	(37° 44' 54.77" N)	127.1651502	Park, Shin, Nam	150716	MK210822
S158	Gelechiidae	*Parastenolechia albicupitella*	Yuklim lake, Jikdong-ri, Soheul-eup, Pocheon-si, Gyeonggi-do, Korea	37.748548	(37° 44' 54.77" N)	127.1651502	Park, Shin, Nam	150716	MK210821
S153	Gelechiidae	*Parastenolechia collucata*	Taeha-ri, Seo-myeon, Ulleung-gun, Gyeongsangbuk-do, Korea	37.5050062	(37° 30' 18.02" N)	130.8277999	Park, Shin, Nam	140807	MK210819
Y218	Gelechiidae	*Parastenolechia collucata*	Yuklim lake, Jikdong-ri, Soheul-eup, Pocheon-si, Gyeonggi-do, Korea	37.748548	(37° 44' 54.77" N)	127.1651502	Park, Nan, Shin, Kim, Son	150528	MK210818
J10	Gelechiidae	*Parastenolechia superba*	Chusan-ri, Okryong-myeon, Gwangyang-si, Jeollanam-do, Korea	35.0467525	(35° 2' 48.31" N)	127.589623	Y.R. Lee	160618	MK210817
Y237	Gelechiidae	*Parastenolechia superba*	Mt. Daeso, Songhyeon-ri, Sowon-myeon, Taean-gun, Chungcheongnam-do, Korea	36.7731411	(36° 46' 23.31" N)	126.1794459	Park, Shin, Kim	150610	MK210811
S292	Gelechiidae	*Parastenolechia superba*	Osan-ri, Gosan-myeon, Wanju-gun, Jeollabuk-do, Korea	35.9586532	(35° 57' 31.15" N)	127.2368809	S.R. Kim	120607	MK210815
S413	Gelechiidae	*Parastenolechia superba*	Sanggye-dong, Nowon-gu, Seoul-si, Korea	37.6780046	(37° 40' 40.82" N)	127.0783244	Kim, Lee, Shin, Roh, Kim	170611	MK210814
S159	Gelechiidae	*Parastenolechia superba*	Yuklim lake, Jikdong-ri, Soheul-eup, Pocheon-si, Gyeonggi-do, Korea	37.748548	(37° 44' 54.77" N)	127.1651502	Park, Nan, Shin, Kim, Son	150528	MK210816
Y178	Gelechiidae	*Parastenolechia superba*	Yuklim lake, Jikdong-ri, Soheul-eup, Pocheon-si, Gyeonggi-do, Korea	37.748548	(37° 44' 54.77" N)	127.1651502	Park, Nan, Shin, Kim, Son	150528	MK210813
Y179	Gelechiidae	*Parastenolechia superba*	Yuklim lake, Jikdong-ri, Soheul-eup, Pocheon-si, Gyeonggi-do, Korea	37.748548	(37° 44' 54.77" N)	127.1651502	Park, Nan, Shin, Kim, Son	150528	MK210812
J49	Xyloryctidae	*Periacma delegata*	Mt. Daeso, Songhyeon-ri, Sowon-myeon, Taean-gun, Chungcheongnam-do, Korea	36.7731411	(36° 46' 23.31" N)	126.1794459	Park, Shin, Kim	150610	MK210808
J50	Xyloryctidae	*Periacma delegata*	Mt. Daeso, Songhyeon-ri, Sowon-myeon, Taean-gun, Chungcheongnam-do, Korea	36.7731411	(36° 46' 23.31" N)	126.1794459	Park, Shin, Kim	150610	MK210807
J51	Xyloryctidae	*Periacma delegata*	Mt. Daeso, Songhyeon-ri, Sowon-myeon, Taean-gun, Chungcheongnam-do, Korea	36.7731411	(36° 46' 23.31" N)	126.1794459	Park, Shin, Kim	150610	MK210806
S312	Gelechiidae	*Pexicopia melitolicna*	Janggok-ri, Gonam-myeon, Taean-gun, Chungcheongnam-do, Korea	36.4424137	(36° 26' 32.69" N)	126.3875925	Park, Shin, Kim	150826	MK210802
S139	Gelechiidae	*Polyhymmo pontifera*	Chusan-ri, Okryong-myeon, Gwangyang-si, Jeollanam-do, Korea	35.0467525	(35° 2' 48.31" N)	127.589623	Y.R. Lee	160618	MK210801
S140	Gelechiidae	*Polyhymmo pontifera*	Chusan-ri, Okryong-myeon, Gwangyang-si, Jeollanam-do, Korea	35.0467525	(35° 2' 48.31" N)	127.589623	Y.R. Lee	160618	MK210800
S141	Gelechiidae	*Polyhymmo pontifera*	Chusan-ri, Okryong-myeon, Gwangyang-si, Jeollanam-do, Korea	35.0467525	(35° 2' 48.31" N)	127.589623	Y.R. Lee	160618	MK210799
S142	Gelechiidae	*Polyhymmo pontifera*	Chusan-ri, Okryong-myeon, Gwangyang-si, Jeollanam-do, Korea	35.0467525	(35° 2' 48.31" N)	127.589623	Y.R. Lee	160618	MK210798
S1	Oecophoridae	*Promalactis albipunctata*	Harye-ri, Namwon-eup, Seogwipo-si, Is. Jeju, Korea	33.3252568	(33° 19' 30.92" N)	126.6020681	Park, Kim, Lee	150810	MK210797
S341	Oecophoridae	*Promalactis albipunctata*	Korea National Arboretum, Jikdong-ri, Soheul-eup, Pocheon-si, Gyeonggi-do, Korea	37.7568443	(37° 45' 24.64" N)	127.1678772	S.J.Roh, Y.M.Shin	170807	MK210796
S144	Oecophoridae	*Promalactis atriplagata*	Mt. Jeombong, Jindong-ri, Girin-myeon, Inje-gun, Gangwon-do, Korea	38.0027202	(38° 0' 9.79" N)	128.4817196	J.O.Lim, K.M.Kim	110704	MK210795
S243	Oecophoridae	*Promalactis atriplagata*	Dong-myeon, Hongcheon-gun, Gangwon-do, Korea	37.6919315	(37° 41' 30.95" N)	127.9726097	S.R. Kim	170616	MK210794
S244	Oecophoridae	*Promalactis atriplagata*	Dong-myeon, Hongcheon-gun, Gangwon-do, Korea	37.6919315	(37° 41' 30.95" N)	127.9726097	S.R. Kim	170616	MK210793
Y143	Oecophoridae	*Promalactis atriplagata*	Mt. Taehwa, Sangrim-ri, Docheok-myeon, Gwangju-si, Gyeonggi-do, Korea	37.3057197	(37° 18' 20.59" N)	127.3028244	S.R.Kim	130615	MK210792
Y49	Oecophoridae	*Promalactis autoclina*	Byungnae-ri Daegwallyeong-myeon, Pyeongchang-gun, Gangwon-do, Korea	37.7174852	(37° 43' 2.95" N)	128.6275487	Y.R. Lee	130814	MK210791

Y51	Oecophoridae	*Promalactis autoclina*	Chusan-ri, Okryong-myeon, Gwangyang-si, Jeollanam-do, Korea	35.0491591	(35° 2' 56.97" N)	127.59797	Y.R. Lee	130726	MK210789
Y52	Oecophoridae	*Promalactis autoclina*	Chusan-ri, Okryong-myeon, Gwangyang-si, Jeollanam-do, Korea	35.0491591	(35° 2' 56.97" N)	127.59797	Y.R. Lee	130726	MK210788
Y55	Oecophoridae	*Promalactis autoclina*	Chusan-ri, Okryong-myeon, Gwangyang-si, Jeollanam-do, Korea	35.0491591	(35° 2' 56.97" N)	127.59797	Y.R. Lee	130726	MK210785
Y53	Oecophoridae	*Promalactis autoclina*	Chusan-ri, Okryong-myeon, Gwangyang-si, Jeollanam-do, Korea	35.0467525	(35° 2' 48.31" N)	127.589623	Y.R. Lee	160618	MK210787
Y54	Oecophoridae	*Promalactis autoclina*	Chusan-ri, Okryong-myeon, Gwangyang-si, Jeollanam-do, Korea	35.0467525	(35° 2' 48.31" N)	127.589623	Y.R. Lee	160618	MK210786
Y56	Oecophoridae	*Promalactis autoclina*	Mt. Seonwang, Naewol-ri, Bigeum-myeon, Sinan-gun, Jeollanam-do, Korea	34.7414992	(34° 44' 29.40" N)	125.9071218	S.Y.Park, J.S.Lim	110830	MK210784
Y59	Oecophoridae	*Promalactis autoclina*	Shinyuk-ri, Jodo-myeon, Jindo-gun, Jeollanam-do, Korea	34.2950549	(34° 17' 42.20" N)	126.0685675	S.Y.Park, J.S.Lim	110720	MK210783
Y50	Oecophoridae	*Promalactis autoclina*	Yongdae recreation forest, Yongdae-ri, Buk-myeon, Inje-gun, Gangwon-do, Korea	38.2328416	(38° 13' 58.23" N)	128.3599232	Y.R. Lee	130801	MK210790
S83	Oecophoridae	*Promalactis bitaenia*	Chusan-ri, Okryong-myeon, Gwangyang-si, Jeollanam-do, Korea	35.0467525	(35° 2' 48.31" N)	127.589623	Y.R. Lee	160618	MK210782
S84	Oecophoridae	*Promalactis bitaenia*	Chusan-ri, Okryong-myeon, Gwangyang-si, Jeollanam-do, Korea	35.0467525	(35° 2' 48.31" N)	127.589623	Y.R. Lee	160618	MK210781
S85	Oecophoridae	*Promalactis bitaenia*	Chusan-ri, Okryong-myeon, Gwangyang-si, Jeollanam-do, Korea	35.0467525	(35° 2' 48.31" N)	127.589623	Y.R. Lee	160618	MK210780
S86	Oecophoridae	*Promalactis bitaenia*	Chusan-ri, Okryong-myeon, Gwangyang-si, Jeollanam-do, Korea	35.0467525	(35° 2' 48.31" N)	127.589623	Y.R. Lee	160618	MK210779
S87	Oecophoridae	*Promalactis bitaenia*	Chusan-ri, Okryong-myeon, Gwangyang-si, Jeollanam-do, Korea	35.0467525	(35° 2' 48.31" N)	127.589623	Y.R. Lee	160618	MK210778
S88	Oecophoridae	*Promalactis bitaenia*	Chusan-ri, Okryong-myeon, Gwangyang-si, Jeollanam-do, Korea	35.0467525	(35° 2' 48.31" N)	127.589623	Y.R. Lee	160618	MK210777
S89	Oecophoridae	*Promalactis bitaenia*	Chusan-ri, Okryong-myeon, Gwangyang-si, Jeollanam-do, Korea	35.0467525	(35° 2' 48.31" N)	127.589623	Y.R. Lee	160618	MK210776
S90	Oecophoridae	*Promalactis bitaenia*	Gulup-ri, Seo-myeon, Hongcheon-gun, Gangwon-do, Korea	37.6224027	(37° 37' 20.65" N)	127.6838362	Y.R. Lee	130702	MK210775
S91	Oecophoridae	*Promalactis enopisema*	Chusan-ri, Okryong-myeon, Gwangyang-si, Jeollanam-do, Korea	35.0467525	(35° 2' 48.31" N)	127.589623	Y.R. Lee	160618	MK210774
S92	Oecophoridae	*Promalactis enopisema*	Chusan-ri, Okryong-myeon, Gwangyang-si, Jeollanam-do, Korea	35.0467525	(35° 2' 48.31" N)	127.589623	Y.R. Lee	160618	MK210773
S93	Oecophoridae	*Promalactis enopisema*	Chusan-ri, Okryong-myeon, Gwangyang-si, Jeollanam-do, Korea	35.0467525	(35° 2' 48.31" N)	127.589623	Y.R. Lee	160618	MK210772
S96	Oecophoridae	*Promalactis enopisema*	Chusan-ri, Okryong-myeon, Gwangyang-si, Jeollanam-do, Korea	35.0467525	(35° 2' 48.31" N)	127.589623	Y.R. Lee	160618	MK210770
S95	Oecophoridae	*Promalactis enopisema*	Gulup-ri, Seo-myeon, Hongcheon-gun, Gangwon-do, Korea	37.6224027	(37° 37' 20.65" N)	127.6838362	Y.R. Lee	130702	MK210771
S76	Oecophoridae	*Promalactis jezonica*	Yongdae recreation forest, Yongdae-ri, Buk-myeon, Inje-gun, Gangwon-do, Korea	38.2360777	(38° 14' 9.88" N)	128.3449028	Y.R. Lee	130731	MK210769
S77	Oecophoridae	*Promalactis jezonica*	Yongdae recreation forest, Yongdae-ri, Buk-myeon, Inje-gun, Gangwon-do, Korea	38.2360777	(38° 14' 9.88" N)	128.3449028	Y.R. Lee	130731	MK210768
S78	Oecophoridae	*Promalactis jezonica*	Yongdae recreation forest, Yongdae-ri, Buk-myeon, Inje-gun, Gangwon-do, Korea	38.2360777	(38° 14' 9.88" N)	128.3449028	Y.R. Lee	130731	MK210767
S79	Oecophoridae	*Promalactis jezonica*	Yongdae recreation forest, Yongdae-ri, Buk-myeon, Inje-gun, Gangwon-do, Korea	38.2360777	(38° 14' 9.88" N)	128.3449028	Y.R. Lee	130731	MK210766
S70	Oecophoridae	*Promalactis odaiensis*	Chusan-ri, Okryong-myeon, Gwangyang-si, Jeollanam-do, Korea	35.0467525	(35° 2' 48.31" N)	127.589623	Y.R. Lee	160618	MK210749
S74	Oecophoridae	*Promalactis odaiensis*	Chusan-ri, Okryong-myeon, Gwangyang-si, Jeollanam-do, Korea	35.0467525	(35° 2' 48.31" N)	127.589623	Y.R. Lee	160618	MK210745
S71	Oecophoridae	*Promalactis odaiensis*	Gulup-ri, Seo-myeon, Hongcheon-gun, Gangwon-do, Korea	37.6224027	(37° 37' 20.65" N)	127.6838362	Y.R. Lee	130702	MK210748
S72	Oecophoridae	*Promalactis odaiensis*	Gulup-ri, Seo-myeon, Hongcheon-gun, Gangwon-do, Korea	37.6224027	(37° 37' 20.65" N)	127.6838362	Y.R. Lee	130702	MK210747
S73	Oecophoridae	*Promalactis odaiensis*	Gulup-ri, Seo-myeon, Hongcheon-gun, Gangwon-do, Korea	37.6224027	(37° 37' 20.65" N)	127.6838362	Y.R. Lee	130702	MK210746
S69	Oecophoridae	*Promalactis odaiensis*	Jeongok-ri, Yeongyang-eup, Yeongyang-gun, Gyeongsangbuk-do, Korea	36.6330773	(36° 37' 59.08" N)	129.1292102	S.R.Kim	140624	MK210750
S49	Oecophoridae	*Promalactis odaiensis*	Yongdae recreation forest, Yongdae-ri, Buk-myeon, Inje-gun, Gangwon-do, Korea	38.2360777	(38° 14' 9.88" N)	128.3449028	Y.R. Lee	130731	MK210765
S50	Oecophoridae	*Promalactis odaiensis*	Yongdae recreation forest, Yongdae-ri, Buk-myeon, Inje-gun, Gangwon-do, Korea	38.2360777	(38° 14' 9.88" N)	128.3449028	Y.R. Lee	130731	MK210764
S51	Oecophoridae	*Promalactis odaiensis*	Yongdae recreation forest, Yongdae-ri, Buk-myeon, Inje-gun, Gangwon-do, Korea	38.2360777	(38° 14' 9.88" N)	128.3449028	Y.R. Lee	130731	MK210763
S52	Oecophoridae	*Promalactis odaiensis*	Yongdae recreation forest, Yongdae-ri, Buk-myeon, Inje-gun, Gangwon-do, Korea	38.2360777	(38° 14' 9.88" N)	128.3449028	Y.R. Lee	130731	MK210762
S54	Oecophoridae	*Promalactis odaiensis*	Yongdae recreation forest, Yongdae-ri, Buk-myeon, Inje-gun, Gangwon-do, Korea	38.2360777	(38° 14' 9.88" N)	128.3449028	Y.R. Lee	130731	MK210761
S58	Oecophoridae	*Promalactis odaiensis*	Yongdae recreation forest, Yongdae-ri, Buk-myeon, Inje-gun, Gangwon-do, Korea	38.2360777	(38° 14' 9.88" N)	128.3449028	Y.R. Lee	130731	MK210759
S59	Oecophoridae	*Promalactis odaiensis*	Yongdae recreation forest, Yongdae-ri, Buk-myeon, Inje-gun, Gangwon-do, Korea	38.2360777	(38° 14' 9.88" N)	128.3449028	Y.R. Lee	130731	MK210758
S60	Oecophoridae	*Promalactis odaiensis*	Yongdae recreation forest, Yongdae-ri, Buk-myeon, Inje-gun, Gangwon-do, Korea	38.2360777	(38° 14' 9.88" N)	128.3449028	Y.R. Lee	130731	MK210757
S61	Oecophoridae	*Promalactis odaiensis*	Yongdae recreation forest, Yongdae-ri, Buk-myeon, Inje-gun, Gangwon-do, Korea	38.2360777	(38° 14' 9.88" N)	128.3449028	Y.R. Lee	130731	MK210756
S62	Oecophoridae	*Promalactis odaiensis*	Yongdae recreation forest, Yongdae-ri, Buk-myeon, Inje-gun, Gangwon-do, Korea	38.2360777	(38° 14' 9.88" N)	128.3449028	Y.R. Lee	130731	MK210755
S64	Oecophoridae	*Promalactis odaiensis*	Yongdae recreation forest, Yongdae-ri, Buk-myeon, Inje-gun, Gangwon-do, Korea	38.2360777	(38° 14' 9.88" N)	128.3449028	Y.R. Lee	130731	MK210754
S65	Oecophoridae	*Promalactis odaiensis*	Yongdae recreation forest, Yongdae-ri, Buk-myeon, Inje-gun, Gangwon-do, Korea	38.2360777	(38° 14' 9.88" N)	128.3449028	Y.R. Lee	130731	MK210753
S66	Oecophoridae	*Promalactis odaiensis*	Yongdae recreation forest, Yongdae-ri, Buk-myeon, Inje-gun, Gangwon-do, Korea	38.2360777	(38° 14' 9.88" N)	128.3449028	Y.R. Lee	130731	MK210752
S67	Oecophoridae	*Promalactis odaiensis*	Yongdae recreation forest, Yongdae-ri, Buk-myeon, Inje-gun, Gangwon-do, Korea	38.2360777	(38° 14' 9.88" N)	128.3449028	Y.R. Lee	130731	MK210751
S55	Oecophoridae	*Promalactis odaiensis*	Yongdae recreation forest, Yongdae-ri, Buk-myeon, Inje-gun, Gangwon-do, Korea	38.2360777	(38° 14' 9.88" N)	128.3449028	Y.R. Lee	130731	MK210760
Y186	Oecophoridae	*Promalactis odaiensis*	Duwoong-wetland, Sindu-ri, Wonbuk-myeon, Taean-gun, Chungcheongnam-do, Korea	36.835705	(36° 50' 8.54" N)	126.1956258	Park, Shin, Kim, Nam	150824	MK210744
S94	Oecophoridae	*Promalactis paiki*	Gulup-ri, Seo-myeon, Hongcheon-gun, Gangwon-do, Korea	37.6224027	(37° 37' 20.65" N)	127.6838362	Y.R. Lee	130702	MK210743
S366	Oecophoridae	*Promalactis subsuzukiella*	Korea National Arboretum, Jikdong-ri, Soheul-eup, Pocheon-si, Gyeonggi-do, Korea	37.7568443	(37° 45' 24.64" N)	127.1678772	S.J.Roh, Y.M.Shin	170720	MK210742
S367	Oecophoridae	*Promalactis subsuzukiella*	Korea National Arboretum, Jikdong-ri, Soheul-eup, Pocheon-si, Gyeonggi-do, Korea	37.7568443	(37° 45' 24.64" N)	127.1678772	S.J.Roh, Y.M.Shin	170720	MK210741
S368	Oecophoridae	*Promalactis subsuzukiella*	Korea National Arboretum, Jikdong-ri, Soheul-eup, Pocheon-si, Gyeonggi-do, Korea	37.7568443	(37° 45' 24.64" N)	127.1678772	S.J.Roh, Y.M.Shin	170720	MK210740
Y184	Oecophoridae	*Promalactis subsuzukiella*	Yuklim lake, Jikdong-ri, Soheul-eup, Pocheon-si, Gyeonggi-do, Korea	37.748548	(37° 44' 54.77" N)	127.1651502	Park, Shin, Nam	150716	MK210739
S2	Oecophoridae	*Promalactis suzukiella*	Bito-ri, Seopo-myeon, Sacheon-gun, Gyeongsangnam-do, Korea	34.9718368	(34° 58' 18.61" N)	127.9739456	Y.M. Park	90623	MK210738
S4	Oecophoridae	*Promalactis suzukiella*	Bito-ri, Seopo-myeon, Sacheon-gun, Gyeongsangnam-do, Korea	34.9718368	(34° 58' 18.61" N)	127.9739456	Y.M. Park	90623	MK210737
Y68	Oecophoridae	*Promalactis suzukiella*	Chusan-ri, Okryong-myeon, Gwangyang-si, Jeollanam-do, Korea	35.0467525	(35° 2' 48.31" N)	127.589623	Y.R. Lee	160618	MK210736
Y73	Oecophoridae	*Promalactis suzukiella*	Mt. Sokri, Samga-ri, Sokriisan-myeon, Boeun-gun, Chungcheongbuk-do, Korea	36.4893203	(36° 29' 21.55" N)	127.8593163	S.R.Kim	110725	MK210732
Y69	Oecophoridae	*Promalactis suzukiella*	Yongdae recreation forest, Yongdae-ri, Buk-myeon, Inje-gun, Gangwon-do, Korea	38.2328416	(38° 13' 58.23" N)	128.3599232	Y.R. Lee	130801	MK210735
Y71	Oecophoridae	*Promalactis suzukiella*	Yongdae recreation forest, Yongdae-ri, Buk-myeon, Inje-gun, Gangwon-do, Korea	38.2328416	(38° 13' 58.23" N)	128.3599232	Y.R. Lee	130801	MK210733
Y70	Oecophoridae	*Promalactis suzukiella*	Yongdae recreation forest, Yongdae-ri, Buk-myeon, Inje-gun, Gangwon-do, Korea	38.2328416	(38° 13' 58.23" N)	128.3599232	Y.R. Lee	130814	MK210734
Y60	Oecophoridae	*Promalactis xiengiansis*	Chusan-ri, Okryong-myeon, Gwangyang-si, Jeollanam-do, Korea	35.0491591	(35° 2' 56.97" N)	127.59797	Y.R. Lee	130726	MK210731
Y61	Oecophoridae	*Promalactis xiengiansis*	Chusan-ri, Okryong-myeon, Gwangyang-si, Jeollanam-do, Korea	35.0491591	(35° 2' 56.97" N)	127.59797	Y.R. Lee	130726	MK210730
S26	Lypusidae	*Pseudatemelia* sp	Wonpung-ri, Yeonpung-myeon, Goesan-gun, Chungcheongbuk-do, Korea	36.8003718	(36° 48' 1.34" N)	128.0487888	S.R.Kim	110630	MK210729
J21	Oecophoridae	*Pseudodoxia achyphanes*	Changcheon-ri, Andeok-myeon, Seogwipo-si, Is. Jeju, Korea	33.2474936	(33° 14' 50.98" N)	126.3655928	Park, Kim, Lee	150723	MK210726
J19	Oecophoridae	*Pseudodoxia achyphanes*	Hannam-ri, Namwon-eup, Seogwipo-si, Is. Jeju, Korea	33.3318179	(33° 19' 54.54" N)	126.6805574	Park, Kim, Lee	140825	MK210728
S40	Oecophoridae	*Pseudodoxia achyphanes*	Jungsan-ri, Daedeok-myeon, Gimcheon-si, Gyeongsangbuk-do, Korea	35.9358889	(35° 56' 9.20" N)	127.9907773	S.R.Kim	120723	MK210721
J25	Oecophoridae	*Pseudodoxia achyphanes*	Napeup-ri, Aewol-eup, Jeju-si, Is. Jeju, Korea	33.4279367	(33° 25' 40.57" N)	126.3402467	Park, Kim, Lee	140724	MK210722
J20	Oecophoridae	*Pseudodoxia achyphanes*	Sanghyo-dong, Seogwipo-si, Is. Jeju, Korea	33.3080637	(33° 18' 29.03" N)	126.5700607	Park, Kim, Lee	140710	MK210727
J22	Oecophoridae	*Pseudodoxia achyphanes*	Sanghyo-dong, Seogwipo-si, Is. Jeju, Korea	33.3080637	(33° 18' 29.03" N)	126.5700607	Park, Kim, Lee	140710	MK210725
J23	Oecophoridae	*Pseudodoxia achyphanes*	Seonheul-ri, Chocheon-eup, Jeju-si, Is. Jeju, Korea	33.5192886	(33° 31' 9.44" N)	126.7042394	Park, Kim, Lee	140710	MK210724
J24	Oecophoridae	*Pseudodoxia achyphanes*	Sinrye-ri, Namwon-eup, Seogwipo-si, Is. Jeju, Korea	33.3354263	(33° 20' 7.53" N)	126.6491917	Park, Kim, Lee	140710	MK210723
S45	Gelechiidae	*Pseudotelphusa acrobrunella*	Byungnae-ri Daegwallyeong-myeon, Pyeongchang-gun, Gangwon-do, Korea	37.7174852	(37° 43' 2.95" N)	128.6275487	Y.R. Lee	130814	MK210718

S46	Gelechiidae	*Pseudotelphusa acrobrunella*	Byungnae-ri Daegwallyeong-myeon, Pyeongchang-gun, Gangwon-do, Korea	37.7174852	(37° 43' 2.95" N)	128.6275487	Y.R. Lee	130814	MK210717
S47	Gelechiidae	*Pseudotelphusa acrobrunella*	Byungnae-ri Daegwallyeong-myeon, Pyeongchang-gun, Gangwon-do, Korea	37.7174852	(37° 43' 2.95" N)	128.6275487	Y.R. Lee	130814	MK210715
Y108	Gelechiidae	*Pseudotelphusa acrobrunella*	Chusan-ri, Okryong-myeon, Gwangyang-si, Jeollanam-do, Korea	35.0491591	(35° 2' 56.97" N)	127.59797	Y.R. Lee	130726	MK210716
Y109	Gelechiidae	*Pseudotelphusa acrobrunella*	Chusan-ri, Okryong-myeon, Gwangyang-si, Jeollanam-do, Korea	35.0491591	(35° 2' 56.97" N)	127.59797	Y.R. Lee	130726	MK210714
S302	Gelechiidae	*Pseudotelphusa acrobrunella*	Chusan-ri, Okryong-myeon, Gwangyang-si, Jeollanam-do, Korea	35.0491591	(35° 2' 56.97" N)	127.59797	Y.R. Lee	130726	MK210720
S305	Gelechiidae	*Pseudotelphusa acrobrunella*	Chusan-ri, Okryong-myeon, Gwangyang-si, Jeollanam-do, Korea	35.0491591	(35° 2' 56.97" N)	127.59797	Y.R. Lee	130726	MK210719
J47	Gelechiidae	*Pseudotelphusa* sp2	Mt. Daeso, Songhyeon-ri, Sowon-myeon, Taean-gun, Chungcheongnam-do, Korea	36.7731411	(36° 46' 23.31" N)	126.1794459	Park, Shin, Kim	150610	MK210713
S211	Oecophoridae	*Schiffermuelleria zelleri*	Haesanryeong, Dongchon-ri, Hwacheon-eup, Hwacheon-gun, Gangwon-do, Korea	38.1896775	(38° 11' 22.84" N)	127.7998277	J.O.Lim, S.M.Oh	130604	MK210711
S212	Oecophoridae	*Schiffermuelleria zelleri*	Yuklim lake, Jikdong-ri, Soheul-eup, Pocheon-si, Gyeonggi-do, Korea	37.748548	(37° 44' 54.77" N)	127.1651502	Park, Lim, Lim	120521	MK210710
S210	Oecophoridae	*Schiffermuelleria zelleri*	Yuklim lake, Jikdong-ri, Soheul-eup, Pocheon-si, Gyeonggi-do, Korea	37.748548	(37° 44' 54.77" N)	127.1651502	Park, Nan, Shin, Kim, Son	150528	MK210712
Y194	Depressariidae	*Scythropiodes approximans*	Korea National Arboretum, Jikdong-ri, Soheul-eup, Pocheon-si, Gyeonggi-do, Korea	37.7520771	(37° 45' 7.48" N)	127.158586	Lee, Park, Lim, Kim, Oh	130613	MK210703
S24	Depressariidae	*Scythropiodes approximans*	Mt. Taehwa, Sangrim-ri, Docheok-myeon, Gwangju-si, Gyeonggi-do, Korea	37.3057197	(37° 18' 20.59" N)	127.3028244	S.R.Kim	130615	MK210704
S332	Depressariidae	*Scythropiodes approximans*	Yonggang-dong, Gyeongju-si, Gyeongsangbuk-do, Korea	35.8697361	(35° 52' 11.05" N)	129.2367455	Park, Shin, Nam, Kim	160613	MK210705
Y12	Depressariidae	*Scythropiodes issikii*	Sangchang-ri, Andeok-myeon, Seogwipo-si, Is. Jeju, Korea	33.2882178	(33° 17' 17.58" N)	126.3588619	Park, Kim, Lee	150618	MK210709
Y5	Depressariidae	*Scythropiodes issikii*	Yeonpyeong-ri, Udo-myeon, Jeju-si, Is. Jeju, Korea	33.4935619	(33° 29' 36.82" N)	126.9587687	Y.M. Park	100907	MK210708
Y7	Depressariidae	*Scythropiodes issikii*	Yeonpyeong-ri, Udo-myeon, Jeju-si, Is. Jeju, Korea	33.5078406	(33° 30' 28.23" N)	126.9635323	Park, Kim, Lee	150614	MK210707
Y8	Depressariidae	*Scythropiodes issikii*	Yeonpyeong-ri, Udo-myeon, Jeju-si, Is. Jeju, Korea	33.5078406	(33° 30' 28.23" N)	126.9635323	Park, Kim, Lee	150614	MK210706
Y33	Depressariidae	Scythropiodes sp1	Jungsan-ri, Daedeok-myeon, Gimcheon-si, Gyeongsangbuk-do, Korea	35.9358889	(35° 56' 9.20" N)	127.9907773	S.R.Kim	120723	MK210701
S311	Depressariidae	*Scythropiodes* sp1	Korea National Arboretum, Jikdong-ri, Soheul-eup, Pocheon-si, Gyeonggi-do, Korea	37.7568443	(37° 45' 24.64" N)	127.1678772	S.J.Roh, Y.M.Shin	170807	MK210702
S100	Stathmopodidae	*Stathmopoda auriferella*	Chusan-ri, Okryong-myeon, Gwangyang-si, Jeollanam-do, Korea	35.0467525	(35° 2' 48.31" N)	127.589623	Y.R. Lee	160618	MK210700
S101	Stathmopodidae	*Stathmopoda auriferella*	Chusan-ri, Okryong-myeon, Gwangyang-si, Jeollanam-do, Korea	35.0467525	(35° 2' 48.31" N)	127.589623	Y.R. Lee	160618	MK210699
S102	Stathmopodidae	*Stathmopoda auriferella*	Chusan-ri, Okryong-myeon, Gwangyang-si, Jeollanam-do, Korea	35.0467525	(35° 2' 48.31" N)	127.589623	Y.R. Lee	160618	MK210698
S103	Stathmopodidae	*Stathmopoda auriferella*	Chusan-ri, Okryong-myeon, Gwangyang-si, Jeollanam-do, Korea	35.0467525	(35° 2' 48.31" N)	127.589623	Y.R. Lee	160618	MK210697
S105	Stathmopodidae	*Stathmopoda auriferella*	Chusan-ri, Okryong-myeon, Gwangyang-si, Jeollanam-do, Korea	35.0467525	(35° 2' 48.31" N)	127.589623	Y.R. Lee	160618	MK210695
S97	Stathmopodidae	*Stathmopoda auriferella*	Chusan-ri, Okryong-myeon, Gwangyang-si, Jeollanam-do, Korea	35.0467525	(35° 2' 48.31" N)	127.589623	Y.R. Lee	160618	MK210694
S98	Stathmopodidae	*Stathmopoda auriferella*	Chusan-ri, Okryong-myeon, Gwangyang-si, Jeollanam-do, Korea	35.0467525	(35° 2' 48.31" N)	127.589623	Y.R. Lee	160618	MK210693
S99	Stathmopodidae	*Stathmopoda auriferella*	Chusan-ri, Okryong-myeon, Gwangyang-si, Jeollanam-do, Korea	35.0467525	(35° 2' 48.31" N)	127.589623	Y.R. Lee	160618	MK210692
Y128	Stathmopodidae	*Stathmopoda auriferella*	Chusan-ri, Okryong-myeon, Gwangyang-si, Jeollanam-do, Korea	35.0467525	(35° 2' 48.31" N)	127.589623	Y.R. Lee	160618	MK210691
Y129	Stathmopodidae	*Stathmopoda auriferella*	Chusan-ri, Okryong-myeon, Gwangyang-si, Jeollanam-do, Korea	35.0467525	(35° 2' 48.31" N)	127.589623	Y.R. Lee	160618	MK210690
S104	Stathmopodidae	*Stathmopoda auriferella*	Chusan-ri, Okryong-myeon, Gwangyang-si, Jeollanam-do, Korea	35.0467525	(35° 2' 48.31" N)	127.589623	Y.R. Lee	160618	MK210696
Y205	Stathmopodidae	*Stathmopoda auriferella*	Mt. Daeso, Songhyeon-ri, Sowon-myeon, Taean-gun, Chungcheongnam-do, Korea	36.7731411	(36° 46' 23.31" N)	126.1794459	Park, Shin, Kim	150610	MK210689
S404	Stathmopodidae	*Stathmopoda commoda*	Korea National Arboretum, Jikdong-ri, Soheul-eup, Pocheon-si, Gyeonggi-do, Korea	37.7506858	(37° 45' 2.47" N)	127.1678128	Lim, Roh, Lee, Choi, Shin	170530	MK210688
S406	Stathmopodidae	*Stathmopoda flavenscens*	Cheongpodae Beach, Woncheong-ri, Nam-myeon, Taean-gun, Chungcheongnam-do, Korea	36.6402712	(36° 38' 24.98" N)	126.3014771	Park, Shin, Kim	150827	MK210686
Y207	Stathmopodidae	*Stathmopoda flavenscens*	Cheongpodae Beach, Woncheong-ri, Nam-myeon, Taean-gun, Chungcheongnam-do, Korea	36.6402712	(36° 38' 24.98" N)	126.3014771	Park, Shin, Kim	150827	MK210684
S405	Stathmopodidae	*Stathmopoda flavenscens*	Janggok-ri, Gonam-myeon, Taean-gun, Chungcheongnam-do, Korea	36.4424137	(36° 26' 32.69" N)	126.3875925	Park, Shin, Kim	150826	MK210687
S407	Stathmopodidae	*Stathmopoda flavenscens*	Mt. Jukyeop, Komo-ri, Soheul-eup, Pocheon-si, Gyeonggi-do, Korea	37.7931837	(37° 47' 35.46" N)	127.1699635	B.W.Lee	130827	MK210685
S250	Stathmopodidae	*Stathmopoda flavenscens*	Naehyeon-ri, Seo-myeon, Yangyang-gun, Gangwon-do, Korea	38.0019129	(38° 0' 6.89" N)	128.6038909	Y.R. Lee	130813	MK210683
S316	Stathmopodidae	*Stathmopoda moriutiella*	Beopheung-ri, Suju-myeon, Yeongwol-gun, Gangwon-do, Korea	37.3659601	(37° 21' 57.46" N)	128.2737367	Shon *et al*.	170727	MK210680
S394	Stathmopodidae	*Stathmopoda moriutiella*	Seungeon-ri, Anmyeong-eup, Taean-gun, Chungcheongnam-do, Korea	36.4999659	(36° 29' 59.88" N)	126.3621608	Park, Shin, Kim, Nam	150826	MK210679
S48	Stathmopodidae	*Stathmopoda neohexatyla*	Byungnae-ri Daegwallyeong-myeon, Pyeongchang-gun, Gangwon-do, Korea	37.7174852	(37° 43' 2.95" N)	128.6275487	Y.R. Lee	130814	MK210671
S262	Stathmopodidae	*Stathmopoda neohexatyla*	Chusan-ri, Okryong-myeon, Gwangyang-si, Jeollanam-do, Korea	35.0491591	(35° 2' 56.97" N)	127.59797	Y.R. Lee	130726	MK210672
S106	Stathmopodidae	*Stathmopoda neohexatyla*	Chusan-ri, Okryong-myeon, Gwangyang-si, Jeollanam-do, Korea	35.0467525	(35° 2' 48.31" N)	127.589623	Y.R. Lee	160618	MK210678
S107	Stathmopodidae	*Stathmopoda neohexatyla*	Chusan-ri, Okryong-myeon, Gwangyang-si, Jeollanam-do, Korea	35.0467525	(35° 2' 48.31" N)	127.589623	Y.R. Lee	160618	MK210677
S108	Stathmopodidae	*Stathmopoda neohexatyla*	Chusan-ri, Okryong-myeon, Gwangyang-si, Jeollanam-do, Korea	35.0467525	(35° 2' 48.31" N)	127.589623	Y.R. Lee	160618	MK210676
S109	Stathmopodidae	*Stathmopoda neohexatyla*	Chusan-ri, Okryong-myeon, Gwangyang-si, Jeollanam-do, Korea	35.0467525	(35° 2' 48.31" N)	127.589623	Y.R. Lee	160618	MK210675
S110	Stathmopodidae	*Stathmopoda neohexatyla*	Chusan-ri, Okryong-myeon, Gwangyang-si, Jeollanam-do, Korea	35.0467525	(35° 2' 48.31" N)	127.589623	Y.R. Lee	160618	MK210674
S111	Stathmopodidae	*Stathmopoda neohexatyla*	Chusan-ri, Okryong-myeon, Gwangyang-si, Jeollanam-do, Korea	35.0467525	(35° 2' 48.31" N)	127.589623	Y.R. Lee	160618	MK210673
S364	Stathmopodidae	*Stathmopoda opticaspis*	Korea National Arboretum, Jikdong-ri, Soheul-eup, Pocheon-si, Gyeonggi-do, Korea	37.7568443	(37° 45' 24.64" N)	127.1678772	S.J.Roh, Y.M.Shin	170720	MK210668
S408	Stathmopodidae	*Stathmopoda opticaspis*	Yuklim lake, Jikdong-ri, Soheul-eup, Pocheon-si, Gyeonggi-do, Korea	37.748548	(37° 44' 54.77" N)	127.1651502	Park, Lim, Lim	120521	MK210667
S361	Stathmopodidae	*Stathmopoda opticaspis*	Yuklim lake, Jikdong-ri, Soheul-eup, Pocheon-si, Gyeonggi-do, Korea	37.748548	(37° 44' 54.77" N)	127.1651502	Park, Shin, Nam	150716	MK210670
S362	Stathmopodidae	*Stathmopoda opticaspis*	Yuklim lake, Jikdong-ri, Soheul-eup, Pocheon-si, Gyeonggi-do, Korea	37.748548	(37° 44' 54.77" N)	127.1651502	Park, Shin, Nam	150716	MK210669
S410	Stathmopodidae	*Stathmopoda opticaspis*	Yuklim lake, Jikdong-ri, Soheul-eup, Pocheon-si, Gyeonggi-do, Korea	37.748548	(37° 44' 54.77" N)	127.1651502	Park, Shin, Nam	150716	MK210666
S363	Stathmopodidae	*Stathmopoda persona*	Korea National Arboretum, Jikdong-ri, Soheul-eup, Pocheon-si, Gyeonggi-do, Korea	37.7568443	(37° 45' 24.64" N)	127.1678772	S.J.Roh, Y.M.Shin	170720	MK210665
S370	Stathmopodidae	*Stathmopoda* sp2	Naemiro-ri, Miro-myeon, Samcheok-si, Gangwon-do, Korea	37.4424244	(37° 26' 32.73" N)	129.0573185	M.S. Oh	170719	MK210664
S369	Stathmopodidae	*Stathmopoda stimulata*	Naemiro-ri, Miro-myeon, Samcheok-si, Gangwon-do, Korea	37.4424244	(37° 26' 32.73" N)	129.0573185	M.S. Oh	170719	MK210663
Y139	Stathmopodidae	*Stathmopoda stimulata*	National D.M.Z Arboretum, Mandae-ri, Haean-myeon, Yanggu-gun, Gangwon-do, Korea	38.2538655	(38° 15' 13.92" N)	128.1122858	S.M.Oh	130726	MK210662
S300	Gelechiidae	*Stenolechia notomochla*	Gulup-ri, Seo-myeon, Hongcheon-gun, Gangwon-do, Korea	37.6224027	(37° 37' 20.65" N)	127.6838362	Y.R. Lee	130702	MK210949
S307	Gelechiidae	*Stenolechia notomochla*	Gulup-ri, Seo-myeon, Hongcheon-gun, Gangwon-do, Korea	37.6224027	(37° 37' 20.65" N)	127.6838362	Y.R. Lee	130702	MK210948
S416	Gelechiidae	*Stenolechia notomochla*	Mt. Daeso, Songhyeon-ri, Sowon-myeon, Taean-gun, Chungcheongnam-do, Korea	36.7731411	(36° 46' 23.31" N)	126.1794459	Park, Shin, Kim	150610	MK210661
Y227	Gelechiidae	*Stenolechia notomochla*	Mt. Daeso, Songhyeon-ri, Sowon-myeon, Taean-gun, Chungcheongnam-do, Korea	36.7731411	(36° 46' 23.31" N)	126.1794459	Park, Shin, Kim	150610	MK210660
S287	Gelechiidae	*Teleiodes linearivalvata*	Chusan-ri, Okryong-myeon, Gwangyang-si, Jeollanam-do, Korea	35.0491591	(35° 2' 56.97" N)	127.59797	Y.R. Lee	130726	MK210659
S288	Gelechiidae	*Teleiodes linearivalvata*	Chusan-ri, Okryong-myeon, Gwangyang-si, Jeollanam-do, Korea	35.0491591	(35° 2' 56.97" N)	127.59797	Y.R. Lee	130726	MK210658
Y175	Gelechiidae	*Teleiodes linearivalvata*	Chusan-ri, Okryong-myeon, Gwangyang-si, Jeollanam-do, Korea	35.0491591	(35° 2' 56.97" N)	127.59797	Y.R. Lee	130726	MK210657
Y95	Gelechiidae	*Teleiodes pekduensis*	Chusan-ri, Okryong-myeon, Gwangyang-si, Jeollanam-do, Korea	35.0491591	(35° 2' 56.97" N)	127.59797	Y.R. Lee	130726	MK210646
Y101	Gelechiidae	*Teleiodes pekduensis*	Chusan-ri, Okryong-myeon, Gwangyang-si, Jeollanam-do, Korea	35.0491591	(35° 2' 56.97" N)	127.59797	Y.R. Lee	130726	MK210656
Y84	Gelechiidae	*Teleiodes pekduensis*	Chusan-ri, Okryong-myeon, Gwangyang-si, Jeollanam-do, Korea	35.0491591	(35° 2' 56.97" N)	127.59797	Y.R. Lee	130726	MK210653
Y85	Gelechiidae	*Teleiodes pekduensis*	Chusan-ri, Okryong-myeon, Gwangyang-si, Jeollanam-do, Korea	35.0491591	(35° 2' 56.97" N)	127.59797	Y.R. Lee	130726	MK210652
Y86	Gelechiidae	*Teleiodes pekduensis*	Chusan-ri, Okryong-myeon, Gwangyang-si, Jeollanam-do, Korea	35.0491591	(35° 2' 56.97" N)	127.59797	Y.R. Lee	130726	MK210651
Y96	Gelechiidae	*Teleiodes pekduensis*	Chusan-ri, Okryong-myeon, Gwangyang-si, Jeollanam-do, Korea	35.0491591	(35° 2' 56.97" N)	127.59797	Y.R. Lee	130726	MK210645
Y98	Gelechiidae	*Teleiodes pekduensis*	Chusan-ri, Okryong-myeon, Gwangyang-si, Jeollanam-do, Korea	35.0491591	(35° 2' 56.97" N)	127.59797	Y.R. Lee	130726	MK210644
Y99	Gelechiidae	*Teleiodes pekduensis*	Chusan-ri, Okryong-myeon, Gwangyang-si, Jeollanam-do, Korea	35.0491591	(35° 2' 56.97" N)	127.59797	Y.R. Lee	130726	MK210643

Y88	Gelechiidae	*Teleiodes pekduensis*	Chusan-ri, Okryong-myeon, Gwangyang-si, Jeollanam-do, Korea	35.0491591	(35° 2' 56.97" N)	127.59797	Y.R. Lee	130726	MK210650
Y89	Gelechiidae	*Teleiodes pekduensis*	Chusan-ri, Okryong-myeon, Gwangyang-si, Jeollanam-do, Korea	35.0491591	(35° 2' 56.97" N)	127.59797	Y.R. Lee	130726	MK210649
Y92	Gelechiidae	*Teleiodes pekduensis*	Chusan-ri, Okryong-myeon, Gwangyang-si, Jeollanam-do, Korea	35.0491591	(35° 2' 56.97" N)	127.59797	Y.R. Lee	130726	MK210648
Y94	Gelechiidae	*Teleiodes pekduensis*	Chusan-ri, Okryong-myeon, Gwangyang-si, Jeollanam-do, Korea	35.0491591	(35° 2' 56.97" N)	127.59797	Y.R. Lee	130726	MK210647
Y131	Gelechiidae	*Teleiodes pekduensis*	Jungsan-ri, Daedeok-myeon, Gimcheon-si, Gyeongsangbuk-do, Korea	35.9358889	(35° 56' 9.20" N)	127.9907773	S.R.Kim	120723	MK210655
Y176	Gelechiidae	*Teleiodes pekduensis*	Yuklim lake, Jikdong-ri, Soheul-eup, Pocheon-si, Gyeonggi-do, Korea	37.748548	(37° 44' 54.77" N)	127.1651502	Park, Nan, Shin, Kim, Son	150528	MK210654
S381	Gelechiidae	*Teleiopsis motleella*	Mt. Geumo, Yeulim-ri, Dolsan-eup, Yeosu-gun, Jeollanam-do, Korea	34.5921879	(34° 35' 31.88" N)	127.8015618	Lim, Choi, Lee, Roh	170612	MK210642
S383	Gelechiidae	*Teleiopsis motleella*	Mt. Geumo, Yeulim-ri, Dolsan-eup, Yeosu-gun, Jeollanam-do, Korea	34.5921879	(34° 35' 31.88" N)	127.8015618	Lim, Choi, Lee, Roh	170612	MK210641
S384	Gelechiidae	*Teleiopsis motleella*	Mt. Geumo, Yeulim-ri, Dolsan-eup, Yeosu-gun, Jeollanam-do, Korea	34.5921879	(34° 35' 31.88" N)	127.8015618	Lim, Choi, Lee, Roh	170612	MK210640
S320	Gelechiidae	*Thiotricha* sp	Mt. Geumo, Yeulim-ri, Dolsan-eup, Yeosu-gun, Jeollanam-do, Korea	34.5921879	(34° 35' 31.88" N)	127.8015618	Lim, Choi, Lee, Roh	170612	MK210639
S414	Gelechiidae	*Tornodoxa* sp	Mt. Toham, Hwangyong-dong, Gyeongju-si, Gyeongsangbuk-do, Korea	35.8154726	(35° 48' 55.70" N)	129.3557298	Park, Shin, Nam, Kim	160614	MK210638
S80	Depressariidae	*Tyrolimnas anthraconesa*	Chusan-ri, Okryong-myeon, Gwangyang-si, Jeollanam-do, Korea	35.0491591	(35° 2' 56.97" N)	127.59797	Y.R. Lee	130726	MK210637
S81	Depressariidae	*Tyrolimnas anthraconesa*	Chusan-ri, Okryong-myeon, Gwangyang-si, Jeollanam-do, Korea	35.0491591	(35° 2' 56.97" N)	127.59797	Y.R. Lee	130726	MK210636
S82	Depressariidae	*Tyrolimnas anthraconesa*	Chusan-ri, Okryong-myeon, Gwangyang-si, Jeollanam-do, Korea	35.0467525	(35° 2' 48.31" N)	127.589623	Y.R. Lee	160618	MK210635

Table S2. Morphological identification of Gelechioidea in Korea, employing recent classification (Kim et al., 2016; Heikkilä et al., 2015)

Higher taxa	Genus	Species	No. of individuals
Aeolanthinae	1	1	4
Autostichiidae	1	5	28
Batrachedridae	1	1	1
Blastobasidae	2	5	25
Coleophoridae	2	8	22
Cosmopterigidae	6	16	42
Depressariidae	6	13	41
Gelechiidae	28	59	152
Lecithoceridae	4	10	25
Lypusidae	1	1	1
Oecophoridae	5	16	86
Stathmopodidae	4	13	50
Xyloryctidae	3	6	32
**13**	**64**	**154**	**509**

Table S3. Results of the Automatic Barcode Gap Discovery (ABGD) analyses. X, relative gap width; Simple, p-distance; Jukes-Cantor substitution model (JC69); Kimura 2-parameter substitution model (K2P).

			Prior intraspecific distance (*P*)						
Model	X	Partition	0.0215	0.0129	0.0077	0.0046	0.0027	0.0016	0.0010
JC	1.5	initial	152	152	152	152	152	152	152
		Recursive	—	154	157	162	174	174	217
K2P	1.5	initial	152	152	152	152	152	152	152
		Recursive	—	154	157	162	172	172	217
Simple	1.5	initial	120	152	152	152	152	152	152
		Recursive	143	152	153	155	160	160	160

Table S4. Additional MOTUs from the multiple delimitation methods, ABGD, PTP and bPTP.

	species	MOTUs		
		ABGD	PTP	bPTP
Aeolanthinae	*Aeolanthes semiostrina*			3
Autostichiidae	*Autosticha pachystica*			2
Blastobasidae	*Neoblastobasis biceratala*	2	2	3
Coleophoridae	*Coleophora trientella*			2
	Coleophora therinella			3
Cosmopterigidae	*Antrachyntis* sp1			2
	Cosmopterix crassicervicella			2
	Cosmopterigidae sp1			3
Depressariidae	*Acria ceramitis*			2
	Agonopterix sp2			2
	Agonopterix vladimiri			2
Gelechiidae	*Aroga mesostrepta*		2	2
	Evippe albidoesella	2	2	2
	Hypatima excellentella			2
	Parachronistis sellaris			2
	Parasternolechia collucata			2
	Polyhymmo pontifera			2
	Pseudotelphusa acrobrunella			2
	Faristenia sp1			3
	Teleiodes linearivalvata			3
	Parastenolechia suriensis			4
	Faristenia jumbongae		2	4
	Mesophleps albilinella			4
	Parachronistis sp			4
	Teleiodes pekduensis			4
Lecithoceridae	*Lecithocera thiodora*			2
	Martyringa ussuriella			2
	Scythropiodes sp1			2
	Lecithoceridae sp1			3
Oecophoridae	*Schiffermuelleria zelleri*			2
	Promalactis atriplagata	2	2	2
	Pseudodoxia achyphanes			5
	Promalactis bitaenia			6
Stathmopodidae	*Atrijuglans hetaohei*		2	2
	Stathmopoda flavenscens			2
	Stathmopoda moriutiella			2
	Stathmopoda opticaspis			3

Figure S1. Maximum likelihood tree with 332 haplotypes of the studied 509 sequences.

[Haplotype: Sequences voucher No.]
[Hap_1: 1 S82]
[Hap_2: 1 S81]
[Hap_3: 1 S80]
[Hap_4: 1 S414]
[Hap_5: 1 S320]
[Hap_6: 1 S384]
[Hap_7: 2 S383 S381]
[Hap_8: 7 Y99 Y98 Y92 Y88 Y84 Y176 Y101]
[Hap_9: 1 Y96]
[Hap_10: 1 Y95]
[Hap_11: 2 Y94 Y89]
[Hap_12: 1 Y86]
[Hap_13: 1 Y85]
[Hap_14: 1 Y131]
[Hap_15: 3 Y175 S288 S287]
[Hap_16: 2 Y227 S416]
[Hap_17: 2 Y139 S369]
[Hap_18: 1 S370]
[Hap_19: 1 S363]
[Hap_20: 5 S410 S408 S364 S362 S361]
[Hap_21: 1 S48]
[Hap_22: 5 S262 S111 S109 S108 S107]
[Hap_23: 1 S110]
[Hap_24: 1 S106]
[Hap_25: 1 S394]
[Hap_26: 1 S316]
[Hap_27: 1 S245]
[Hap_28: 1 S152]
[Hap_29: 4 S250 Y207 S406 S405]
[Hap_30: 1 S407]
[Hap_31: 1 _S404]
[Hap_32: 8 Y205 Y128 S99 S98 S105 S103 S101 S100]
[Hap_33: 1 Y129]
[Hap_34: 1 S97]
[Hap_35: 1 S104]
[Hap_36: 1 S102]
[Hap_37: 1 Y33]
[Hap_38: 1 S311]
[Hap_39: 2 Y194 S24]
[Hap_40: 1 S332]
[Hap_41: 2 Y8 Y12]
[Hap_42: 1 Y7]
[Hap_43: 1 Y5]

[Hap_44:	2	S212 S210]
[Hap_45:	1	S211]
[Hap_46:	1	J47]
[Hap_47:	1	Y109]
[Hap_48:	1	S47]
[Hap_49:	1	Y108]
[Hap_50:	2	S46 S45]
[Hap_51:	1	S305]
[Hap_52:	1	S302]
[Hap_53:	5	S40 J23 J21 J20 J19]
[Hap_54:	3	J25 J24 J22]
[Hap_55:	1	S26]
[Hap_56:	1	Y61]
[Hap_57:	1	Y60]
[Hap_58:	1	Y73]
[Hap_59:	1	Y71]
[Hap_60:	1	Y70]
[Hap_61:	1	Y69]
[Hap_62:	1	Y68]
[Hap_63:	1	S4]
[Hap_64:	1	S2]
[Hap_65:	4	Y184 S368 S367 S366]
[Hap_66:	1	S94]
[Hap_67:	1	Y186]
[Hap_68:	1	S74]
[Hap_69:	1	S73]
[Hap_70:	1	S72]
[Hap_71:	1	S71]
[Hap_72:	1	S70]
[Hap_73:	1	S69]
[Hap_74:	7	S67 S66 S61 S58 S54 S51 S49]
[Hap_75:	3	S65 S64 P_odaeiensis_S59]
[Hap_76:	1	S62]
[Hap_77:	1	S60]
[Hap_78:	1	S55]
[Hap_79:	1	S52]
[Hap_80:	1	S50]
[Hap_81:	2	S79 S77]
[Hap_82:	1	S78]
[Hap_83:	1	S76]
[Hap_84:	1	S96]
[Hap_85:	1	S95]
[Hap_86:	3	S93 S92 S91]
[Hap_87:	1	S90]
[Hap_88:	5	S89 S88 S87 S86 S83]
[Hap_89:	1	S85]

[Hap_90: 1 S84]
[Hap_91: 1 Y59]
[Hap_92: 1 Y56]
[Hap_93: 3 Y55 Y51 Y50]
[Hap_94: 2 Y54 Y52]
[Hap_95: 1 Y53]
[Hap_96: 1 Y49]
[Hap_97: 2 Y143 S144]
[Hap_98: 2 S244 S243]
[Hap_99: 1 S341]
[Hap_100: 1 S1]
[Hap_101: 3 S142 S140 S139]
[Hap_102: 1 S141]
[Hap_103: 1 S312]
[Hap_104: 1 S156]
[Hap_105: 1 S155]
[Hap_106: 1 S154]
[Hap_107: 2 J51 J50]
[Hap_108: 1 J49]
[Hap_109: 1 J18]
[Hap_110: 1 J16]
[Hap_111: 2 Y237 Y179]
[Hap_112: 1 Y178]
[Hap_113: 2 S413 J10]
[Hap_114: 1 S292]
[Hap_115: 1 S159]
[Hap_116: 2 Y218 S153]
[Hap_117: 1 S185]
[Hap_118: 1 S158]
[Hap_119: 1 S157]
[Hap_120: 1 S296]
[Hap_121: 1 S289]
[Hap_122: 1 S252]
[Hap_123: 2 S190 S151]
[Hap_124: 4 Y232 Y231 Y228 Y226]
[Hap_125: 2 S417 S412]
[Hap_126: 1 S187]
[Hap_127: 1 J9]
[Hap_128: 1 J8]
[Hap_129: 1 S360]
[Hap_130: 1 S314]
[Hap_131: 1 S313]
[Hap_132: 1 Y91]
[Hap_133: 1 Y90]
[Hap_134: 1 Y203]
[Hap_135: 1 S30]

[Hap_136:	1	S336]
[Hap_137:	1	S334]
[Hap_138:	1	S143]
[Hap_139:	1	S135]
[Hap_140:	3	S335 J54 J46]
[Hap_141:	1	s134]
[Hap_142:	1	J53]
[Hap_143:	4	Y217 Y216 Y206 S267]
[Hap_144:	1	Y212]
[Hap_145:	5	S37 S333 S32 S31 S238]
[Hap_146:	1	S35]
[Hap_147:	1	S33]
[Hap_148:	1	S27]
[Hap_149:	1	S183]
[Hap_150:	3	J58 J57 J52]
[Hap_151:	1	J56]
[Hap_152:	1	J4]
[Hap_153:	1	S353]
[Hap_154:	4	S132 S131 S130 S128]
[Hap_155:	1	S129]
[Hap_156:	1	S127]
[Hap_157:	2	Y105 Y104]
[Hap_158:	1	S359]
[Hap_159:	1	Y34]
[Hap_160:	5	Y28 Y25 Y24 Y170 J31]
[Hap_161:	1	Y27]
[Hap_162:	1	Y26]
[Hap_163:	2	S207 S206]
[Hap_164:	1	J3]
[Hap_165:	2	Y193 S23]
[Hap_166:	1	S225]
[Hap_167:	5	S403 S402 S401 S399 S374]
[Hap_168:	1	S221]
[Hap_169:	2	S400 S239]
[Hap_170:	1	S372]
[Hap_171:	2	Y167 Y141]
[Hap_172:	1	S165]
[Hap_173:	1	Y240]
[Hap_174:	1	S319]
[Hap_175:	4	Y117 Y115 S349 J55]
[Hap_176:	1	S350]
[Hap_177:	1	S348]
[Hap_178:	3	Y125 Y123 Y122]
[Hap_179:	1	Y124]
[Hap_180:	1	Y121]
[Hap_181:	1	Y120]

[Hap_182:	1	Y119]
[Hap_183:	1	Y118]
[Hap_184:	1	Y114]
[Hap_185:	1	S189]
[Hap_186:	1	J6]
[Hap_187:	2	Y192 S409]
[Hap_188:	1	S137]
[Hap_189:	1	S136]
[Hap_190:	1	S347]
[Hap_191:	1	S309]
[Hap_192:	1	S308]
[Hap_193:	1	S222]
[Hap_194:	2	J2 J1]
[Hap_195:	1	Y236]
[Hap_196:	1	Y183]
[Hap_197:	1	Y182]
[Hap_198:	1	Y181]
[Hap_199:	1	S301]
[Hap_200:	1	S299]
[Hap_201:	1	Y180]
[Hap_202:	1	Y165]
[Hap_203:	1	S415]
[Hap_204:	1	S382]
[Hap_205:	1	S378]
[Hap_206:	1	S307]
[Hap_207:	1	S300]
[Hap_208:	3	S380 S379 S310]
[Hap_209:	1	J5]
[Hap_210:	1	J45]
[Hap_211:	1	J44]
[Hap_212:	3	Y235 S295 S179]
[Hap_213:	1	S294]
[Hap_214:	1	S200]
[Hap_215:	1	S186]
[Hap_216:	1	Y93]
[Hap_217:	3	Y83 Y82 Y102]
[Hap_218:	1	Y134]
[Hap_219:	2	Y107 Y106]
[Hap_220:	1	Y187]
[Hap_221:	1	S256]
[Hap_222:	1	S199]
[Hap_223:	1	S329]
[Hap_224:	3	Y209 S373 S357]
[Hap_225:	1	S422]
[Hap_226:	1	S421]
[Hap_227:	1	S328]

[Hap_228:	3	Y140 J29 J28]
[Hap_229:	2	Y67 S209]
[Hap_230:	1	Y66]
[Hap_231:	1	Y173]
[Hap_232:	1	Y159]
[Hap_233:	1	Y158]
[Hap_234:	1	Y157]
[Hap_235:	1	S194]
[Hap_236:	1	S193]
[Hap_237:	1	J26]
[Hap_238:	1	S43]
[Hap_239:	1	S181]
[Hap_240:	9	Y169 Y168 S227 J37 J36 J35 J34 J33 J30]
[Hap_241:	1	J38]
[Hap_242:	1	J32]
[Hap_243:	1	S419]
[Hap_244:	1	Y242]
[Hap_245:	1	S318]
[Hap_246:	2	S343 S342]
[Hap_247:	1	Y190]
[Hap_248:	1	Y191]
[Hap_249:	3	Y189 S391 _S218]
[Hap_250:	1	S351]
[Hap_251:	3	S344 S317 S182]
[Hap_252:	1	S315]
[Hap_253:	1	Y221]
[Hap_254:	1	S395]
[Hap_255:	1	S389]
[Hap_256:	1	S426]
[Hap_257:	1	S425]
[Hap_258:	2	S424 S423]
[Hap_259:	4	Y222 Y188 S385 S219]
[Hap_260:	1	Y220]
[Hap_261:	4	S217 S215 S214 S213]
[Hap_262:	1	S393]
[Hap_263:	1	S392]
[Hap_264:	1	S387]
[Hap_265:	1	Y219]
[Hap_266:	1	S216]
[Hap_267:	1	S386]
[Hap_268:	2	J11 S286]
[Hap_269:	1	S298]
[Hap_270:	1	S297]
[Hap_271:	1	S191]
[Hap_272:	2	S197 S188]
[Hap_273:	1	Y137]

[Hap_274:	1	Y136]
[Hap_275:	1	Y135]
[Hap_276:	4	Y225 Y215 J14 J12]
[Hap_277:	1	S375]
[Hap_278:	1	J15]
[Hap_279:	1	J13]
[Hap_280:	1	Y164]
[Hap_281:	1	Y163]
[Hap_282:	1	Y130]
[Hap_283:	1	Y112]
[Hap_284:	1	S390]
[Hap_285:	1	S192]
[Hap_286:	1	S184]
[Hap_287:	1	Y1]
[Hap_288:	4	Y233 Y230 Y229 J48]
[Hap_289:	1	Y21]
[Hap_290:	2	Y177 J41]
[Hap_291:	1	J39]
[Hap_292:	9	Y20 Y36 Y19 Y18 Y14 S398 S396 S356 Y16]
[Hap_293:	1	Y243]
[Hap_294:	1	Y17]
[Hap_295:	1	Y156]
[Hap_296:	1	Y155]
[Hap_297:	1	Y15]
[Hap_298:	1	Y13]
[Hap_299:	1	S397]
[Hap_300:	3	Y4 Y3 Y2]
[Hap_301:	1	S340]
[Hap_302:	2	S339 S338]
[Hap_303:	1	S337]
[Hap_304:	1	Y202]
[Hap_305:	1	S229]
[Hap_306:	3	Y199 Y198 Y201]
[Hap_307:	1	Y197]
[Hap_308:	1	Y196]
[Hap_309:	2	Y195 S418]
[Hap_310:	1	S224]
[Hap_311:	1	S411]
[Hap_312:	1	Y162]
[Hap_313:	4	S9 S7 S6 S11]
[Hap_314:	1	S8]
[Hap_315:	1	S10]
[Hap_316:	2	S327 S326]
[Hap_317:	1	S325]
[Hap_318:	2	Y161 Y160]
[Hap_319:	1	Y42]

[Hap_320:	1	S15]
[Hap_321:	1	S14]
[Hap_322:	1	S13]
[Hap_323:	5	Y45 Y38 S20 S19 S18]
[Hap_324:	1	Y44]
[Hap_325:	2	S358 S21]
[Hap_326:	1	S352]
[Hap_327:	4	Y151 Y150 Y149 Y148]
[Hap_328:	1	Y146]
[Hap_329:	1	Y138]
[Hap_330:	1	S324]
[Hap_331:	2	S323 S322]
[Hap_332:	1	S321]

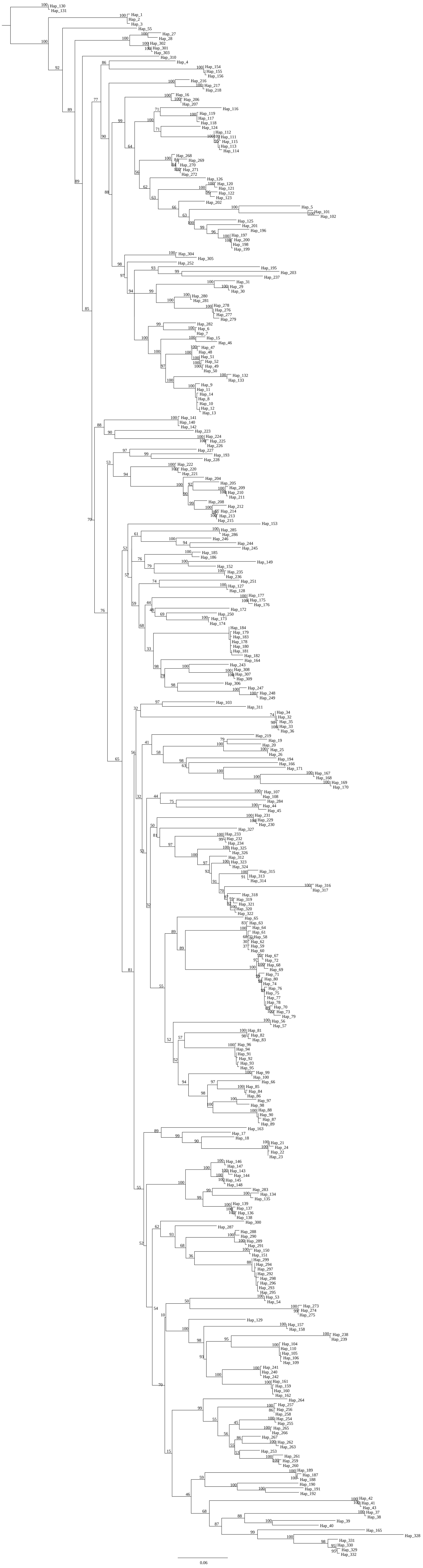
0.06

Figure S2. Maximum likelihood tree based on 509 sequences.
Numbers on the branches are bootstrap percentages (%) from the ML analysis.

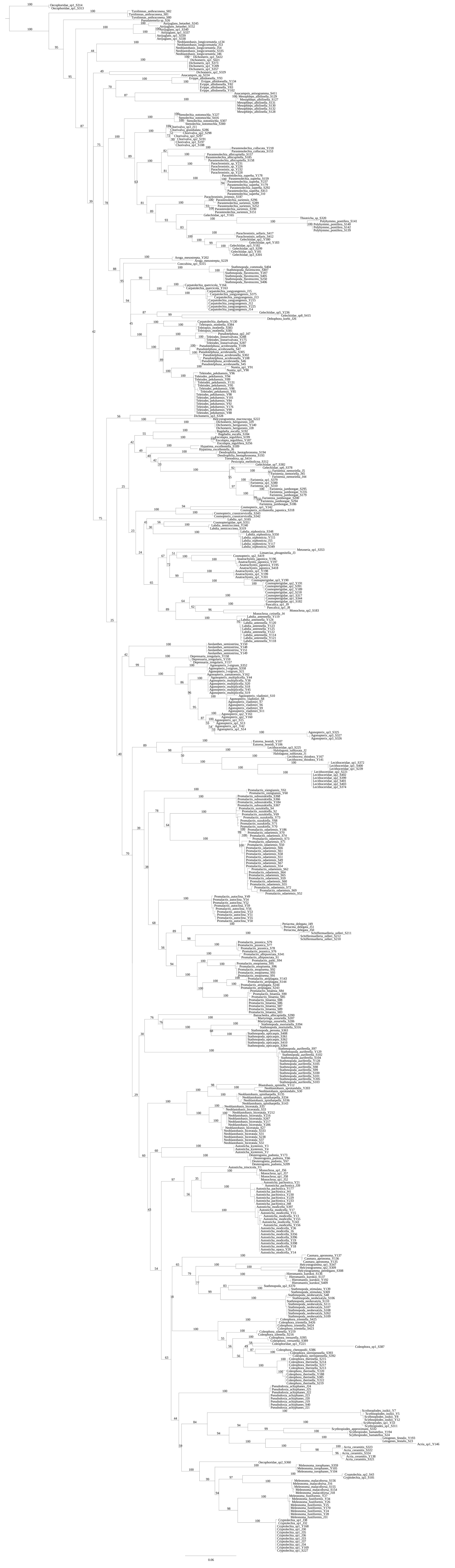

Supplementary legends

Table S1. A total of 509 sequences for 154 morphospecies were generated as novel data in the present study including GenBank Accession Numbers and GPS information.

Table S2. Morphological identification of Gelechioidea in Korea, employing recent classification (Kim et al., 2016; Heikkilä et al., 2015).

Table S3. Results of the Automatic Barcode Gap Discovery (ABGD) analyses. X, relative gap width; Simple, p-distance; Jukes-Cantor substitution model (JC69); Kimura 2-parameter substitution model (K2P).

Table S4. Additional MOTUs from the multiple delimitation methods, ABGD, PTP and bPTP.

Figure S1. Maximum likelihood tree with 332 haplotypes of the studied 509 sequences.

Figure S2. Maximum likelihood tree based on 509 sequences.
